# Getting Hold of the Tobamovirus Particle—Why and How? Purification Routes over Time and a New Customizable Approach †

**DOI:** 10.3390/v16060884

**Published:** 2024-05-30

**Authors:** Tim Wendlandt, Beate Britz, Tatjana Kleinow, Katharina Hipp, Fabian J. Eber, Christina Wege

**Affiliations:** 1 Institute of Biomaterials and Biomolecular Systems, Molecular and Synthetic Plant Virology, University of Stuttgart, Pfaffenwaldring 57, 70569 Stuttgart, Germany; tim.wendlandt@bio.uni-stuttgart.de (T.W.); beate.britz@mpa.uni-stuttgart.de (B.B.); tatjana.kleinow@bio.uni-stuttgart.de (T.K.); 2 Electron Microscopy Facility, Max Planck Institute for Biology Tübingen, Max-Planck-Ring 5, 72076 Tübingen, Germany; katharina.hipp@tuebingen.mpg.de; 3 Department of Mechanical and Process Engineering, Offenburg University of Applied Sciences, Badstr. 24, 77652 Offenburg, Germany; fabian.eber@hs-offenburg.de

**Keywords:** tobacco mosaic virus (TMV), turnip vein clearing virus (TVCV), plant virus, history, purification, isolation, precipitation, ultracentrifugation, density gradients, iodixanol

## Abstract

This article develops a multi-perspective view on motivations and methods for tobamovirus purification through the ages and presents a novel, efficient, easy-to-use approach that can be well-adapted to different species of native and functionalized virions. We survey the various driving forces prompting researchers to enrich tobamoviruses, from the search for the causative agents of mosaic diseases in plants to their increasing recognition as versatile nanocarriers in biomedical and engineering applications. The best practices and rarely applied options for the serial processing steps required for successful isolation of tobamoviruses are then reviewed. Adaptations for distinct particle species, pitfalls, and ‘forgotten’ or underrepresented technologies are considered as well. The article is topped off with our own development of a method for virion preparation, rooted in historical protocols. It combines selective re-solubilization of polyethylene glycol (PEG) virion raw precipitates with density step gradient centrifugation in biocompatible iodixanol formulations, yielding ready-to-use particle suspensions. This newly established protocol and some considerations for perhaps worthwhile further developments could serve as putative stepping stones towards preparation procedures appropriate for routine practical uses of these multivalent soft-matter nanorods.

## 1. Introduction

How did early researchers achieve the purification of virions from ‘tobacco mosaic-diseased’ plants until a fundamental understanding of the helical tobamovirus particle structure was reached? Have the initial protocols experienced a continuous ‘evolution’ and optimization throughout more than 125 years of tobamovirus research, or have new technologies and scientific questions driven major changes in purification strategies? Which approaches and combinations thereof have ever been tested for the different serial processing steps, from leaf tissue homogenization up to the formulation of the final virion preparations? And last but not least: Is there still room for further improvements with regard to current scientific challenges and emerging practical uses of the robust, multivalent nucleoprotein particles?

These questions have arisen from our involvement in both teaching and research and have prompted us to work out a sketch map on tobamovirus purification strategies applied over time, in conjunction with a case study demonstrating the high potential of ‘old’, but topically refreshed methods for an efficient, customized isolation of engineered viruses with properties deviating from the parental ones. The principle of polyethylene glycol (PEG) raw precipitate re-solubilization in inverse PEG concentration gradients [1,2] has recently been taken up by our team for the fast and gentle enrichment of a tobacco vein clearing virus (TVCV) variant with *Staphylococcus aureus* protein A-domains fused to all of its coat protein subunits (CPs; TVCV_PA_ [3]). On this basis, we have now implemented an advantageous, simplified procedure using iodixanol step gradients for selective virion banding, yielding stable, ready-to-use particle suspensions, as described below (Section 4). This article section might encourage further efforts and process developments that profit from historic achievements.

Our initial TVCV_PA_ purification via the almost forgotten inverse PEG solubility-concentration gradients had been proposed and supported by Holger Jeske as a scientific mentor, collaborator, and friend. He also suggested its further streamlining and devoted extensive lab work and many hours at the transmission electron microscope (TEM) himself to the development of preparative and analytical techniques for novel types of tobacco mosaic virus (TMV)-based assemblies, e.g., [4,5,6,7,8,9,10,11,12,13,14,15,16,17,18,19,20,21,22]. Well-known and highly active primarily in geminivirus research, Holger continuously raised awareness for the rarely opened treasure trove of early, but surprisingly sophisticated (bio-)chemical, physical, and molecular methods in their historical contexts. He also appreciated and participated gladly in the establishment of our research focus on novel uses and in vitro derivatives of tobamovirus particles as a spin-off from his department in Stuttgart, for which we dedicate this article to his memory.

### 1.1. What Can (and Cannot) Be Expected from This Article?

**Figure 1:**
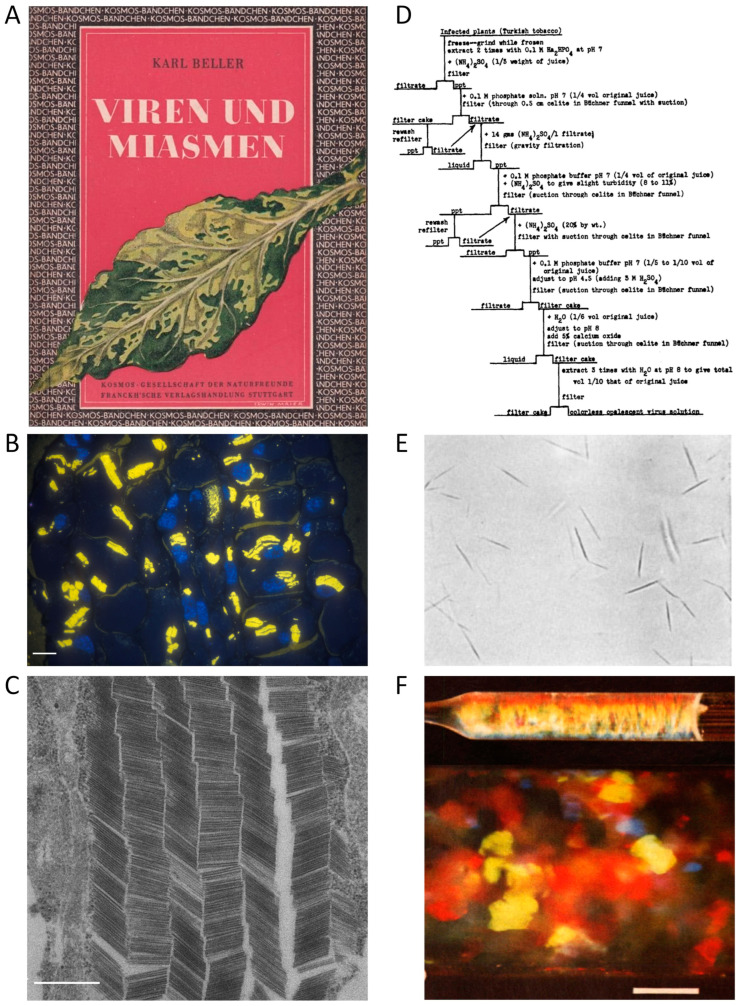
Arrangement of TMV virions into highly ordered aggregates [syn. (para)crystals, liquid crystals] in infected plant cells and after purification in vitro. (**A**) Cover page of a popular scientific booklet on ‘Viruses and Miasmata’, published in 1949 in Stuttgart, Germany: contemporary drawing of *N. tabacum* leaf showing symptoms of the tobacco mosaic disease. Copyright 2024 by Franckh-Kosmos Verlags-GmbH & Co. KG, Stuttgart, Germany on behalf of Franckh’sche Verlagshandlung, W. Keller & Co., Stuttgart. (**B**,**C**) Aggregates of TMV virions arranged in layered rows in infected plant cells, visualized by fluorescence microscopy and electron microscopy (EM), respectively. (**B**) TMV was detected by immunofluorescence (yellow) on sections prepared for EM (scale bar 10 µm, cell nuclei are stained blue with DAPI). (**C**) Transmission EM (TEM) of ultrathin sections showing the regular arrays of TMV particles in leaf cells (scale bar 500 nm). Images in (**B**,**C**) are kindly provided by Heinz Schwarz, MPI for Biology Tübingen, Tübingen, Germany. (**D**) Overview of a classical protocol used by Stanley (1936) for TMV virion isolation via a series of salt and acid precipitations. Reproduced from Steere, 1959 [23], with minor changes. Copyright 2024 by Copyright Clearance Center, Inc., Danvers, U.S. on behalf of Elsevier Science & Technology Journals, Amsterdam, Netherlands. (**E**) Photomicrograph of crystalline aggregates formed in vitro from purified TMV (strain acuba) particles at 393× magnification. Reproduced from Stanley (1937) [24] with minor changes. This work is made available under the terms of the Creative Commons Attribution-NonCommercial 4.0 Unported License, https://creativecommons.org/licenses/by/4.0/, accessed on 7 March 2024. (**F**) Iridescent TMV gels with different micro- and macrocrystalline structural phases (nematic and smectic liquid crystals) formed in vitro from purified virions [25]. Diffraction of visible light by iridescent gel (**upper panel**) and by gel domains of randomly oriented macrocrystals with different diffraction colors (**lower panel**); scale bar 0.5 mm. Reproduced from Kreibig and Wetter 1980 [25] with slight modifications. This work is made available under the terms of the Creative Commons Attribution-NonCommercial 4.0 Unported License, https://creativecommons.org/licenses/by/4.0/, accessed on 7 March 2024.

This article provides an overview of methods for isolating tobamoviruses from their hosts, which started with the earliest experiments on tobacco showing striking leaf variegation and spot or ‘mosaic’ symptoms (Figure 1A). These initial studies from the 1880s onwards attempted to separate the disease-inducing agent (detectable easily by modern microscopy now: Figure 1B,C) from plant sap, but were unsuccessful until the 1930s. Notwithstanding, the era of ‘fishing in increasingly clearer water’ facilitated the development of viable virion purification methods. These methods are described with regard to technical and conceptual advances, and the first understanding of viruses as a novel class of biological entities (Section 1.2). The subsequent Section 1.3 covers the period from the first precipitation of infectious TMV paracrystals (Figure 1D,E) to the present. It surveys the history of two decades, when different laboratories hunted for the ‘compound’ called TMV and eventually understood its helical organization from CP subunits protecting an RNA. The RNA turned out to be the genetic blueprint of the virus. Despite these fundamental conceptual achievements on the self-encoding but host-dependent tobamovirus structure, methods of TMV purification did not change substantially during this period, as briefly described. However, they enabled the growth of further research areas on tobamoviruses, which, after the mid-1950s, broadened quickly and spread around the world (as the viruses did themselves). It is impossible to review all these scientific developments, so we highlight only a few major upcoming fields of work that have promoted novel techniques and refinements of established virion isolation routes. Conversely, TMV has also been an ideal ‘guinea pig’ for testing and implementing novel purification procedures in different contexts. In the last thirty years, new applications of tobamoviruses in biomedicine and nanotechnology have increased the demand for purified particles. The corresponding recent areas of research and development (R&D) are described in greater detail before we summarize the portfolio of now widely used virion isolation methods to conclude the historic overview in Section 1.3. The last introductory paragraphs (Section 1.4) will outline the biological and physicochemical particle properties, as well as the diversity of distinct tobamoviruses that has arisen through the ages, and provide the keys to appropriate purification strategies.

Section 2 dives more deeply into the principles and application histories of major operations to access and store the desired whitish-opalescent colloidal tobamovirus sols. As purification protocols combine different procedures serially, a rough overview of relevant process chains is given in Section 3, which also hints at potential pitfalls, a few ‘neglected’ and some peculiar TMV isolation approaches. Our case study on a beneficial, new combination of uncommon purification steps involving PEG precipitate re-solubilization in iodixanol-based density step gradients effectively adaptable to specific virion properties is presented in Section 4. It might add some more value to ‘forgotten’ methods as sources for new procedures, as introduced above. Finally, Section 5 looks ahead and suggests potential directions in tobamovirus nanoparticle production, which, however, will face a trade-off between promising application prospects and current regulatory issues.

Originally, we set out to obtain a reliable survey of purification methods applied in different scientific contexts, labs, and time periods. The more we read, though, the more gaps in our reviewing approach became obvious. Hence, despite a broad literature search, we will have missed relevant information. Nevertheless, more than 10,000 entries (out of a total of about ≈250,000 on tobacco mosaic [virus] available in English or German in the Google Scholar database up to February 2024) mentioning virion preparation by different wording and existing collections of our teams were the source for more than 600 handpicked articles as the main starting material. These covered 30 to 150 publications per decade from the 1930s onward. These publications were sifted for tobamovirus purification methods and complemented by articles on tobamoviruses other than TMV according to references in the International Committee on Taxonomy of Viruses (ICTV) database. In addition, hard copies available for selected non-digitalized historic papers, and methodological reports and reviews were also accessed. This *modus operandi* could, however, not avoid bias due to the non-accessibility of various articles through our institutions, publications in languages other than English or German, missing or wrongly referenced descriptions of purification procedures, and, unexpectedly, many studies even on wildtype TMV were not found via our search strings because the virus name was not explicitly included in the title, abstract, or keywords. Our work has, however, carved out a multifaceted overview of the history of tobamovirus purification, which we hope will inspire many new ideas and debates—as it already did in the authors’ team.

### 1.2. The Cryptic ‘Poisonous’ Agent Causing Tobacco Mosaic Disease: Early ‘Pre-Purification’ Experiments towards Virus Separation

Experiments to isolate the agent causing ‘tobacco mosaic disease’ in the Netherlands may be regarded as the key to the whole discipline of virology. After Adolf E. Mayer had discovered the transmissibility of the disease to previously healthy plants and speculated about its origin in the 1880s (as reviewed in detail by [26]), attempts to separate the responsible agent from plant sap by Chamberland filter candles developed for water sterilization [27] were carried out in different labs. As the porous porcelain failed to remove infectivity and since both cultivation and diffusion tests on agar plates did not indicate any ‘corpuscular’, bacterial pathogen, Martinus W. Beijerinck claimed the existence of a ‘contagium vivum fluidum’ inducing the ‘tobacco spot disease’, in 1898 [28]. This was ground-breaking because, despite his misconception of a ‘living fluid’, Beijerinck proposed a completely new type of infectious ‘virus’ (i.e., ‘poisonous’ agent) that passed through filtration pores small enough to retain microbes. His foresight of viruses as a novel class of biological agents was in clear contrast to earlier [29] and also subsequent conclusions of the Russian researcher Dmitri I. Ivanowski, from similar filtration tests, who was convinced of a bacterial origin of the disease, as reviewed, e.g., in [26,30,31,32]. Hence, Beijerinck’s study is now widely recognized as the foundation of virology, e.g., [33,34,35,36,37], the more so, as it also stated ‘that propagation results only when the virus is connected with the living and growing protoplasm of the host-plant’ [28]. Ivanowski is given credit for the first documented conclusion of an unusually small infectious or toxic entity responsible for the symptoms of tobacco, reported already in 1892 [31,33,36]. However, his strong belief in a microbial origin, strengthened by agar diffusion tests of infectious plant sap in comparison to similarly mobile solid ink particles, kept him on the wrong track for at least another decade [26]. In 1903, he published a series of agar cultivation/plant inoculation tests and microscopic images from his Ph.D. (dissertation) work, which led him to conclude that colonies of short rod-shaped bacteria growing in filaments represented the mosaic-inducing ‘contagium’ [38]. Notwithstanding, he concomitantly discovered and showed inclusion bodies in the plasma of infected leaf cells that exhibited transverse striation after acidic staining (compare with Figure 1C). Hence, he was very close to the real pathogenic agent but did not grasp its scientific novelty [31]. An understanding of viruses as biochemical, but non-living entities was proposed only three years later, in 1906, by the German geneticist Erwin Baur [39], on the basis of his and earlier grafting experiments with ornamental, mosaic-diseased Abutilon plants (outlined in [40]). He hypothesized transmissible ‘viruses’ to be substances produced and accumulated by the affected plant itself—a modern description still valid.

Approaches to isolate the infectious agent from leaves by filtration, cultivation on nutrient media, and passage further in plants have thus played central roles already in the earliest investigations on TMV and its relatives. To obtain information on virus structure and composition, plant sap, homogenates, and filtrates, as well as soil from pots containing diseased plants, were analyzed by microscopy and underwent various treatments (e.g., heat, acid, glycerol, ethanol, or formalin), before they were tested for residual infectivity [28,29,41,42]. Such experiments comprised the majority of studies on tobacco and increasing numbers of other plant ‘mosaic diseases’ during the first three decades of the 20th century, once termed ‘the no man’s land before molecular biology got off the ground’, by Lute Bos [26]. However, in those years, progress in separation technologies suggested other methods for accessing pathogens that passed bacterial filters (called ‘filterable’ at that time, i.e., not being retained). Such agents were also found to be responsible for animal diseases, including foot-and-mouth disease, rabies, and pox. As early as 1907, gel filtration in media based on gelatin or cellulose nitrate (collodium) was proposed as an ‘ultrafiltration’ method with high promise for fractionating those pathogens [43]. However, protocols for a reproducible preparation of tobamoviruses sufficiently pure for molecular and biochemical work and for structural studies were implemented only from the 1930s onward. Before this, the well-known local lesion assay allowing the quantification of infectious TMV [33,44] and the neutralizing anti-TMV antisera raised in rabbits [33,45] were further fundamental discoveries that contributed to a better understanding of the virus before its purification.

### 1.3. Tobamovirus Purification over Time: How and Why

#### 1.3.1. From Crystal-like Needles to ‘Ribonucleoprotein’ Helices

Right time, right person, right place—and the right portion of luck: In the early 1930s, the chemist Wendell M. Stanley accepted the challenge to extract the TMV compound at the Rockefeller Institute for Medical Research in Princeton, New Jersey—a scientific environment with experience on enzyme purification and crystallization, as reviewed in more detail elsewhere [26,33,36]. Stanley worked on this in parallel with other researchers in a number of laboratories in the U.S., England, and Australia, some of them very close to purifying TMV. Carl George Vinson in the U.S. had already obtained infectious crystals [46,47]. However, Stanley was the first to publish a specific procedure giving rise to needles of a ‘crystalline protein’ (Figure 1E) with the ‘properties of tobacco mosaic virus’, ≈30 µm in length [48,49]. These were obtained initially from an ammonium sulfate-precipitate of juice from diseased Turkish tobacco plants, following repeated suspension and re-precipitation, including the use of lead subacetate and diatom silica (Celite) for the removal of plant components, and finally the addition of acidified ammonium sulfate. Stanley tested a number of similar protocols (with the flowchart of one such method from 1936 shown in Figure 1D), yielding essentially the same type of needles. These could be ‘re-crystallized’ 15 times without loss of activity and were reproduced in several hundred batches within the next three years, with comparable outcomes and ‘identical physical, chemical, biological and serological properties’ [50]. Although Stanley believed in the infectious nature of a protein alone, he was honored for his achievement and awarded the Nobel Prize for Chemistry in 1946. Shortly after Stanley’s publication, his colleague Ralph Wyckoff achieved an even faster purification of the virus at the same institute. Based on the pioneering development of analytical ultracentrifuges more than a decade earlier by Theodor Svedberg in Sweden [51], Wyckoff and co-workers had constructed a simpler air-driven prototype [52]. In accordance with results obtained in Sweden shortly before [53], Wyckoff developed a TMV isolation procedure mainly via differential centrifugation [54].

In parallel and only a few months after Stanley’s major report on purified TMV ‘protein’, Frederick C. Bawden, Norman W. Pirie, and others at the Rothamsted Experimental Station in England identified RNA as the second main constituent of TMV particles, which, however, initially was not accepted by Stanley [33,55,56,57]. The horserace and dispute over the initial isolation and biochemical characterization of TMV would be worth a much more detailed description not feasible in the course of this review.

TMV had also been the subject of many biophysical experiments before the Second World War, with an important impact on the development of cutting-edge analytical technologies and instruments. Its rod-like, charged structure was deduced from double refraction observed by Takahashi and Rawlins for virus-containing plant sap already in 1932 [50], from X-ray studies on the fibrous precipitates with their ‘apparent crystallinity’ [56]—better referred to as ‘paracrystallinity’ [58], and later also by quantitative optical double refraction measurements for virus suspensions in flow [59] or electric fields [60]. Capillary viscosimeters revealed orientation-dependent mechanical properties of the anisotropic TMV colloids in a shear flow [61,62], with their liquid-crystalline phase behavior characterized in much detail soon thereafter [63], as was their interaction with different types of colloids such as gold particles [64]. These routes of tobamovirus research are still contemporary in novel contexts, e.g., [20,65,66,67,68]. Figure 1F illustrates the captivating appearance of TMV gels with crystalline status [25]. In most of these early studies, TMV was either enriched via salt-based precipitation as the major separation step, similar to the procedure of Stanley [49], and/or by differential centrifugation. Ultracentrifuges had soon been acquired by many labs worldwide, so both the more laborious high-speed as well as the easier-to-handle low-speed centrifugation have been combined with biochemical methodologies [60,69,70,71].

The availability of new preparative equipment and expertise on plant virus purification occurred in the same period when three different groups in Berlin constructed distinct types of analytically useful electron microscopes, after the initial invention of such an instrument with, however, only 16-fold magnification achieved in 1932 by Ernst Ruska and Max Knoll at the Technical University of Berlin (as reviewed in detail [72]). After several bacteria and three types of orthopoxviruses, TMV and potato virus X (PVX) became the first plant viruses studied initially in a pre-serial TEMat 10–15 nm resolution [73] and with 7 nm resolution in a commercially distributed instrument shortly thereafter [72]. The first TEM visualization confirmed the rod-like shape of TMV and indicated dimensions of ‘around 300 respective 150 × 15 mµ’ [i.e., nm] of the ‘molecules of the TM-virus’ [73]. Since then, tobamoviruses have been among the viral objects most intensely used for EM analyses, as their robustness, accurate diameters, early use in immunological studies, and high availability did not only ensure fruitful studies on the virus particle and its self-assembly, but also suggested TMV as an excellent model object for the development of novel preparative and analytical EM methodologies [32,72,74].

After the first electron-optical inspection of TMV, however, it required almost two further decades of analytical and conceptual progress before a consistent understanding of the supramolecular particle structure was established in the late 1950s. The initial and fundamentally new assumption of TMV as helical assembly was published in 1954 by James Watson, who had considered data for the virus structure from earlier (namely Bernal and Fankuchen’s data from Birkbeck/London [63], see below) and his own X-ray diffraction patterns obtained in Cambridge with methodological guidance by Francis Crick [75]. Watson, however, could not yet localize the RNA inside and hypothesized its longitudinal insertion in the center of the virions [75]. Building on the growing body of work in several institutes, X-ray scattering experiments by the British physical chemist Rosalind Franklin and the American biophysicist Donald Caspar performed in London, England, eventually led to a model of TMV as a ribonucleoprotein helix with a correctly integrated RNA. Franklin and Caspar described the position of the RNA between the CP subunits at a radius of 40 nm in two consecutive papers in *Nature* in 1956, as reviewed elaborately [33,75]. The actual number of 49 CPs per three helical turns was reported about a year later by R. Franklin, Aaron Klug, and Kenneth C. Holmes [76] in the course of a Ciba Foundation meeting on viruses in London, which brought together members of all four leading laboratories working on fundamental virus research in Western countries after the Second World War: Berkeley (U.S.), Cambridge and Birkbeck/London (U.K.), and Tübingen (Germany) [75].

After her precise description of TMV virions together with Klug and Holmes, Franklin died only a year later without being recognized broadly [75]. She and her co-workers’ scientific route towards the TMV structure had been prepared by cutting-edge data, conceptual ideas, and purified tobamovirus particles from the labs participating in the Ciba Foundation meeting. In Birkbeck, Bernal and Fankuchen had already discussed initial evidence for ‘repeat units’ within the elongated particles in 1941 [63], although TMV was typically regarded as a single protein molecule at that time. The TMV preparations applied in their crystallography/X-ray work were purified by their British colleagues Bawden and Pirie from pre-clarified plant sap via an extensive series of repetitive precipitations, including treatments with alcohol, HCl and NaOH, ammonium sulfate, and NaCl. The resulting ‘nearly colorless and slightly opalescent’ preparations contained 1 to 2 g of TMV from every liter of plant sap [56]. Complementary early evidence for TMV consisting of different subunits came from Germany, where research on viruses was done originally at the Kaiser-Wilhelm-Institute in Berlin-Dahlem by Adolf Butenandt, Alfred Kühn, and Fritz von Wettstein under the department heads Gerhard Schramm and Georg Melchers working on TMV from 1938 [33,77]. In 1943, during World War II, this work was relocated to Tübingen in southern Germany and became the Max Planck Institute (MPI) of Virus Research in 1950, where fundamental molecular discoveries on tobamoviruses were achieved mainly from the mid-1950s to the 1980s, before the institute was re-oriented towards developmental biology (as German plant virology had settled at other places with the teams of Karl-Wolfgang Mundry and, from 1993 on, Holger Jeske, nearby in Stuttgart—the scientific origin of most of the authors of this article). In 1943, Schramm described the disassembly of TMV into smaller building blocks under alkaline conditions and published their re-assembly four years later [33]. He purified TMV (if specified) mainly via ultracentrifugation [78]. Due to his arrangement with the Nazi regime and the multifaceted relations between those German virologists who continued their work during the Third Reich and the governmental authorities, as well as with international colleagues (reviewed thoroughly and rich in nuances [33,79]), the recognition and impact of Schramm’s and his co-workers’ extensive experiments in the 1940s (published mostly in German) remained very limited.

This changed only slowly in the 1950s, during the difficult return of German researchers into international scientific communities, which profited from influential emigrated colleagues [79]. Largely in parallel to the X-ray diffraction studies eventually revealing the correct helical arrangement of the TMV building blocks, Schramm and his collaborator Alfred Gierer, in close scientific exchange with Melchers and Hans Friedrich-Freksa (all directors/division leaders at the MPI in Tübingen in or after the 1950s), obtained various evidence for the RNA as genetic information encoding the virus. The path to this discovery was not straightforward. In 1956, Schramm and Gierer published experimental evidence that TMV RNA isolated from virions caused lesions on tobacco leaves [80,81], which is sometimes regarded as the most fundamental German contribution to tobamovirus research [79]. In a previous report on the infectivity of alkali-treated, partially uncoated TMV particles, Schramm et al. already proposed the decisive role of the RNA in 1955 in German [82]. This preceded a similar suggestion in 1956 [83] by their competitor, Heinz Fraenkel-Conrat, at the virology department at Berkeley, founded by Stanley. In his short letter devoid of original experimental data, Fraenkel-Conrat states that the RNA fraction released from TMV particles ‘is now regarded’ as responsible for the infectivity of such preparations—three weeks before the submission of two detailed scientific articles by the Germans [80,81]. Subsequently, Fraenkel-Conrat and colleagues succeeded in assembling hybrid TMV particles in vitro that trans-encapsidated the RNA of TMV from distinct strains and caused the plant symptoms associated with the source of RNA rather than CP. However, in the initial reports on this finding, they qualified their previous interpretation of RNA as the relevant coding material [79]. In 1957, Fraenkel-Conrat, Singer, and Williams published an extensive confirmative study on the ‘infectivity of viral nucleic acid’ [84]. Both Fraenkel-Conrat and Schramm were jointly awarded the New York Academy of Sciences Lasker Prize in 1958. While Schramm’s work used virus particles enriched by ultracentrifugation, Fraenkel-Conrat did not specify their initial purification from plants but employed ultracentrifugation as an important separation technique in the course of their experiments [84].

Until the fundamental structure of TMV was resolved, virion isolation and analysis were not only brought forward and optimized in the ‘Western world’, but also in Russia, Japan, and other countries. The history of TMV in the ‘non-Western’ regions after Ivanowsky’s disagreement with Beijerinck, however, is not covered by most current review articles due to the more difficult accessibility of relevant articles and a lack of translated digital resources. Exemplary interesting work comprises the purification of distinct TMV variants in the first Japanese air-driven vacuum ultracentrifuge, including a detailed biophysical characterization of the particles in Tokyo as published in 1953 [85] and EM studies on TMV and other plant viruses using ammonium sulfate-precipitated materials in Hokkaido two years later [86]. In Russia, experiments on TMV liquid crystals were reported in 1941, with, however, the details of virus purification not simply accessible [87], as is also the case for precipitation tests with TMV-containing liquids published by the USSR Academy of Sciences in 1950 [88].

Understanding both composition and structure has been a primary motivation for purifying immense amounts of tobamoviruses up to the middle of the last century [89]. Already in parallel to these investigations, however, curiosity about the principles of function of such viruses, their obviously unique physicochemical properties, and the increasing awareness of their importance as pathogens have become further driving forces in ‘tobamovirology’. Hence, many different types of experiments soon required isolated virions, which has prompted researchers worldwide to identify purification protocols best suited for the intended purpose or the lab where the work was performed. The following section, therefore, will focus on only a few of them.

#### 1.3.2. Manifold Routes of Research Driving Technical Progress Thereafter

In parallel to the emergence of a consistent picture on structurally and functionally important particle properties in the late 1950s, the fascinating, widely recognized work on TMV inspired many researchers worldwide to tread novel paths in tobamovirus research (worked out thoroughly elsewhere, e.g., in [32,33,34,35,46,90]). This resulted in a plenitude of molecular investigations on the viral infection cycle, its genetically encoded interplay with plant components, and the identification of more and more tobamoviruses in various crops, ornamentals, and wild plants. Efforts to resolve the respective virion structures at increased resolution, and investigations of their behavior under different chemical and physical treatments, namely with regard to their assembly and disassembly, were directly associated with a continuous need for purified tobamovirus particles in laboratories worldwide. This soon entailed almost countless numbers of published studies, so only a few exemplary lines of research can be touched on here, all of which contributed to the diversification and improvement of virion isolation strategies (see Section 2). Technical progress as a complementary driving force has also affected purification protocols. The most recent increase in publications on TMV and further tobamoviruses is treated in a separate section: the use of viral nanotubes in nanotechnology and biomedical applications (see Section 1.3.3).

The purposes for which tobamovirus particles have been purified from infected plant material have varied over time. In the early days, until the advent of molecular biological methods, virions were mostly used to determine their family and species by various analytical procedures [91,92,93], and references in Table S1. Newly discovered tobamovirus species were distinguished from those already known by distinct names or ‘strain descriptors’, and further characterized if necessary. Analytical approaches were based on the particles’ serological reactivity, the amino acid composition of the CP, and infection experiments to reveal the host plant spectrum and symptom development in diagnostic hosts (syn. ‘indicator plants’, a concept introduced in 1931 [94]).

Tobamovirus virions have a strong immunogenic effect and are serologically different [92,93]. Therefore, purified particles were employed in the 1960s to generate specific antisera for diagnostic applications, which are widely used still today in various test formats for rapid monitoring of infections [91,95,96,97]. Many current standard protocols for the detection and identification of plant viruses, including tobamoviruses, published, e.g., by the European Plant Protection Organization (EPPO), describe antisera/antibody-based techniques such as enzyme-linked immunosorbent assay (ELISA) or immunoelectron microscopy (EPPO Standards—PM 7 Diagnostics: PM 7/125(1) ELISA tests for plant viruses; PM 7/126(1) Electron microscopy in diagnosis of plant viruses).

Isolated virions have been the subject of structural investigations from the early experimental stages, e.g., via X-ray crystallography, EM, light diffraction, light scattering, or flow birefringence analyses [25,63,98,99,100,101,102,103,104]. They have repeatedly served as model particles for scrutinizing novel technologies, which has, e.g., given rise to a 2.3 Å-resolved TMV structure upon testing the power of a direct electron detector in cryo-EM single-particle analysis [105]. Tobamovirus particles were also analyzed qualitatively by mass spectrometry, allowing the identification of variants with mutated CPs within a population, which would be missed by standard methods such as SDS-PAGE [106,107]. For structural examination, virion purification techniques often had to be adapted to the special requirements of the respective analytical methods (for more details and case studies, see Section 3.1).

Purified virions have also been the starting material for isolating their single-stranded (ss) genomic RNA molecules of sense polarity (+). The obtained viral RNA was initially characterized mainly by electrophoretic techniques, and with the development of high-performance molecular biology tools, viral RNA then served as a template for cloning, sequencing of full-length genomes, and the generation of plant-infectious constructs. Within recent decades, a rapidly increasing amount of sequence information has become the main source of identification of new species and for extensive phylogenetic analyses of tobamoviruses [97,108,109,110]. Virion isolation has become largely unnecessary for monitoring tobamovirus diversity and evolution due to the establishment of powerful and sensitive methods for direct amplification of cDNA from viral RNA by reverse transcription-polymerase chain reaction (RT-PCR). High-throughput next generation sequencing (NGS) methods have opened up alternative ways for the unambiguous identification of tobamoviruses at the species and quasi-species levels [95], and references in Table S1.

#### 1.3.3. Novel Applications, Increasing Demand: Tobamovirus Particles as Tools

Starting in the 1980s and accelerating substantially since the turn of the millennium, a conspicuous international renaissance of tobamovirus research has been observed: TMV and related taxa have been ‘refurbished’ in several virology research teams, and have also been established in labs not investigating plant viruses before. This is due to the rise of novel expression and nanotechnologies that make viral derivatives attractive tools and building blocks in materials and devices with highly diverse application prospects. These have boosted the demand for tobamovirus nanoparticles considerably and are thus outlined in greater detail before we look at the virion purification methods employed (in Section 1.3.4).

In 1989, T. Michael A. Wilson at the John Innes Institute (now Centre) in Norwich, U.K., reviewed plant virus-based ‘designer functions’ that had developed fast in the previous few years [111]. The pioneering work included repurposed tobamovirus-derived elements such as the TMV packaging signals applied for the protection of heterologous RNAs in pseudovirus particles, the viral RNA’s omega leader used as a translational enhancer, TMV sequences promising for pathogen-derived plant protection strategies, and the first study on the use of TMV CP fusion proteins as carriers for foreign epitopes; see [111] for original references. The foresighted work of Haynes et al. in Ontario, Canada, yielded the first self-assembling TMV-based polio vaccine from an *E. coli*-expressed CP fusion protein, inducing neutralizing antibodies in rats [112]. Since then, tobamovirus-derived particles have increasingly been recognized as robust but tailorable, sustainably produced, and biodegradable multivalent nanoscaffolds, with a multitude of biomedical and technical applications tested with highly promising prospects [113]. Technology-oriented research and developments include the use of viral nanoparticles (VNPs) and virus-like particles (VLPs) as biotemplates for inorganic and organic compounds and as carriers for biomolecules [11]. Thirty years of creative developments have given rise to an enormous variety of novel colloidal (particulate), layered, and volume materials, either active by themselves as hybrid structures or after integration into technical devices [114,115,116,117,118,119,120,121,122,123,124,125]. The following showcases only a small selection of the many exciting overviews and cutting-edge studies.

Biomedical uses of tobamovirus derivatives were evaluated for both human and veterinary treatments, as well as in phytopathology, with auspicious trials at the animal and laboratory testing levels [115,126,127]. They spanned the range from vaccines, adjuvants, and test antigens [128,129,130,131,132,133] to the intravital delivery of tobamoviral diagnostic imaging agents and therapeutics, with cutting-edge multitasking and theranostic approaches [134,135]. Inactivated tobacco mild green mosaic virus (TMGMV) showed agronomic promise for a root-directed supply of pesticides [136], with the plus that the replicating TMGMV was also approved as an herbicide against invasive plants in the U.S. due to its limited risk of spread and its natural occurrence [137]. This would make inefficient virus inactivation due to technical failures manageable. The primary advantages of tobamoviral (and other plant viral) delivery systems consist of their particle structure with cargo transport possible in the virion inner channel, on the outer surface of more than 2000 repetitively arranged, selectively addressable CP subunits [138], and in-between these subunits [139]. Various applications may also benefit from the intriguing options for shape design, enabled through in vitro assembly of tobamovirus derivatives [118], their high and further tailorable biocompatibility with an increasing understanding of tobamovirus pharmacology following medical administration [122,140], and many means of multifunctionalization, e.g., with cell-targeting molecules, tracer, and effector compounds. Therefore, tobamoviruses also hold great opportunities in scaffold-assisted tissue and organoid engineering technologies to generate implants and model structures for personalized treatments. The rod-like VNPs exert beneficial effects on cells cultivated on planar substrates and in 3D hydrogels and can guide the differentiation of progenitor cells in response to their contact with peptides presented on the viral backbones [141,142]. Recently, tobamoviral scaffolds were combined with other plant viral effectors into osteogenesis-promoting hydrogels that were able to direct the formation of mineralized bone tissue-like entities [143].

Beyond such uses with living organisms or cellular structures, tobamovirus particles have been employed as high-surface-area biotemplates and nanocarriers in various kinds of technical devices and composite materials, several of them with close-to-application status. Hydrated and especially dried TMV have shown a robustness that often surpassed all initial expectations [118,144,145], so that even electrospun polymer fibers with biofunctionalized TMV [146], nanostructured nickel-coated surfaces for efficient boiling heat transfer [147], or long-term durable biosensors with TMV adapter elements [148,149,150] have become available.

In tobamovirus-aided fabrication, virions initially served as richly available templates for the deposition of hard compounds, which yielded mineralized tubes around TMV cores, by 1999 [151,152]; nanowires inside the central channel a few years later [21]; and numerous types of functional composites with different inorganic and organic materials thereafter [153]. While native tobamoviruses were used originally, chemically and genetically modified virions soon enabled a better-controlled interaction or linkage with heterologous compounds, as well as their integration into technical devices [11,116,117,125,154,155,156,157,158]. Many of these profited from considerable surface enhancement and good steric accessibility of the nanorods equipped, e.g., with coatings of conductive or catalytically active metals, semi-conductors or alloys [18,22,159,160,161], chromophores [162], polymers [163], and even fullerenes [164] or metal-organic frameworks [165]. The resulting ‘smart materials’ and virus-assisted devices have been prepared for applications in fundamental research [166], energy storage and conversion [167,168], data memory [169], environmental remediation [170], as antireflective coatings [171], ferrofluids with increased magnetoviscosity [67], fibers for metal nanoparticle production, and many more, as given in exemplary review articles [172,173,174,175].

In combination with biomolecules such as peptides, antibodies, and enzymes, tobamoviruses have been applied not only in medical contexts, as outlined above, but have shown an equally promising track record in the fabrication of new types of biosensors, enzymatically active nanoparticles, and materials for bioaffinity enrichment or deposition [11,118,150]. Virions displaying genetically fused or chemically installed peptides on their outer CP surfaces were shown to either nucleate a selective mineralization of different materials [12,176], or direct self-ligation with complementary peptide tags, as demonstrated by the *Streptococcus pyogenes*-based SpyTag/SpyCatcher (ST/SC) system [177] for the immobilization of fungal enzymes on TMV [178]. Such a peptide-mediated coupling of larger proteins is one of a few biomolecular workarounds to overcome restrictions in the size and charge of genetic fusions to the TMV CP, which are known to abolish systemic infectivity and/or virus assembly for most amino acid chains longer than 23 residues. Longer fluorescent proteins or receptor-binding protein domains could be installed on a sub-set of the CPs by advanced expression constructs integrating either ‘ribosomal skip’ sequences into the TMV RNA or by peptide linkers counteracting steric and charge constraints, reviewed in more detail in [113,179,180]. Tobacco vein clearing virus (TVCV), a subgroup 3 tobamovirus (see below), accepted a C-terminal extension of its CP by 148 amino acids to directly display two IgG-binding domains of *S. aureus* protein A (PA) on all of the CP subunits [181]. The systemically infectious, immunoadsorbent particles could be equipped conveniently with a two-enzyme system by way of enzyme-recognizing antibody conjugates captured by the PA domain shell [3].

TMV and TVCV particles, with sensor enzymes installed by the above approach at high surface densities, or via chemical and bioaffinity linkage, have shown excellent functionality as adapter coatings in microtiter plate-based colorimetric assays and in different label-free electrochemical biosensor layouts. The exposure of different enzymes converting antibiotics, sugars, urea, or fermentation markers on tobamoviral carriers enhanced sensor performance and reusability considerably in comparison to sensors with conventionally deposited enzymes [149,150,182,183,184,185,186]. TMV stabilized the sensor characteristics up to a year of repetitive application without loss of sensitivity in the case of a field-effect sensor for penicillin [148]. Similarly beneficial effects of tobamo-VLP-scaffolded receptor layers were observed for impedimetric on-chip sensors [187] and in an optical microdisk resonator setup [155] for the label-free detection of antibodies via VLP-exposed peptides. The detection system could be transferred into capillary flow-loaded microfluidic devices [188], paving the way towards portable TMV-enhanced detectors. All-soft, liquid-permeable biosensors are a further layout recently enabled by TMV rods, which retained signal-generating sensor enzymes in an immersed porous hydrogel matrix [189]. Finally, the RNA-guided in vitro assembly of TMV-like particles offers unique access to uncommon nanorod-derived straight and kinked structures with controllable ‘arm’ lengths, as well as to nanorods with selectively addressable longitudinal subdomains, as reviewed in detail [118]. The latter may be generated in precise lengths by way of dynamic DNA-assisted nanotechnology [6], which allows, e.g., the display of well-defined biomolecule ensembles, and has a large potential for fundamental research [190].

#### 1.3.4. Few Protocols for Many Uses? State-of-the-Arts of ‘Standard’ Purification Methods

The huge number of rapidly developing tobamovirus applications led us to assume that only a few particle purification protocols have become standard during recent years, due to their ease, efficiency, speed, low requirements for instrumentation, or simply their good description in widely recognized studies. However, this guess has been confirmed only partially by a small set of ‘test drillings’ in technology-oriented publications from distinct areas of research and different countries: Virion precipitation from pre-cleared plant homogenates, often by way of PEG (Section 2.3), in combination with differential and, in some studies, density gradient or cushion centrifugation (Section 2.4), seems most frequently employed all over the world [138,142,166,168,176,180,191,192,193,194,195,196,197]. Nevertheless, a considerable variety of conditions applied in the serial processing steps are often found even for a single tobamovirus species, and different protocols are applied by individual research groups. This may be due to a specific need for narrow particle length distributions obtainable by, e.g., zonal centrifugation (i.e., separation according to sedimentation coefficients) or uses requiring superior removal or avoidance of certain contaminants (Section 2.4 and Section 3.1). Such might also account for the co-existence of several clearing treatments for the plant raw extract, ranging from organic solutes via heat to mineral adsorbants (see Section 2.2). In other cases, though, the choice of a specific protocol might simply reflect the ready-to-hand literature, the availability of a certain PEG type in the cupboard, or personal experience—in order not to change what is known to work (Materials and Methods sections typically do not contain too many arguments). Alternative strategies for the preparation of tobamovirus particles are not extinct either. For example, some researchers precipitate virions with ammonium sulfate [198,199,200], others refrain from any precipitation and enrich virions with different consecutive centrifugation steps. If a background of accompanying compounds from plants is tolerated or even preferred in the work, such protocols can be quite simple [201]. In the opposite situation, if highly pure virus is needed, e.g., for ultrastructural analyses [202], centrifugal enrichment may comprise an extensive series of differential sedimentation and resuspension steps, essentially according to [203]. In more recent work, high-resolution cryo-EM maps have also been obtained from PEG-precipitated TMV, but after its post-purification via repetitive differential centrifugation [104,204], to get rid of residual polymer left on the virus particles.

Hence, our conclusions on contemporary purification protocols for tobamovirus particles may be kept short: the use of PEG is most widespread so that G.V. Gooding, Jr., and T.T. Hebert from the North Carolina State University, USA [205], and S.N. Chapman from the James Hutton Research Institute, U.K. (formerly Scottish Crop Research Institute) [206] will remain well-known to the next generations of plant virologists (see Section 2.3). However, many variations on the theme exist, and considerably different virion isolation approaches are likely to survive.

### 1.4. Tobamovirus Properties and Variability—Essential Knowledge

Knowledge of the in-planta accumulation and biological diversity of tobamoviruses with deviating physical and biochemical properties is crucial for well-adapted and successful purification protocols, as described below.

The virions often form large para-crystalline inclusions of elongated or plate-like shape in infected plant cells, which are easily recognizable by light microscopy and reveal their densely packed particle arrangement under the electron microscope at high resolution (references in Table S1, Figure 1B,C and [207]). Virions per se show liquid-crystalline properties ([25] and references therein, [56,63,208]). In planta, they form liquid crystals of 2D smectic order (hexagonal crystals), whereas the crystalline phases obtained for purified virions in vitro are mostly nematic [98,208,209,210]. Kreibig and Wetter [25] investigated the crystalline properties of six different tobamovirus species by optical diffraction. They showed that purified virions from all tested species formed iridescent gels with different micro- and macrocrystalline structural phases in vitro (nematic and smectic liquid crystals, Figure 1F). Examination by optical diffraction indicated a crystalline multilayer structure similar to the immature crystal forms in living plant cells.

Tobamovirus particles have a diameter of approximately 18 nm; their predominant lengths are 300–312 nm, depending on the virus species [211,212,213,214]. Encapsidation of subgenomic RNAs results in shorter VLPs (32–40 nm), which typically make up only a small proportion of the virus population [215,216,217,218]. The straight, rod- (precisely: tube-)-shaped particles have a helical organization and are built from the viral genomic ssRNA strand and multiple copies of a single coat protein (CP) of 1.70–1.80 × 10^4^ Dalton (156–161 amino acids). The TMV particle, as an example, contains about 2130 CP subunits that are tightly packed into a rigid tube 300 nm long, with an inner and outer diameter of 4 nm and 18 nm, respectively. The helical virion structure contains 16 1/3 protein subunits/turn and a clockwise pitch of 2.3 nm [219].

Different, position-dependent types of CP–CP and CP–RNA interactions contribute to its high stability [104,202,220]. A (+) ssRNA genome of 6.3 to 6.5 kb length is ‘sandwiched’ in-between the CP helix at a radius of approximately 4 nm, with three nucleotides associated with each protein subunit [211,212,213,214,221]. This repetitive supramolecular arrangement is visualized clearly elsewhere, e.g., in [222,223], and described more concisely in our previous review [118]. The end of the tobamovirus particle containing the 3’-terminus of the genome is convex in shape, while the other end is concave [224]. The viral RNA constitutes ca. 5% of the particle weight and encodes at least three proteins: the 126-kDa replicase protein and its readthrough of 183-kDa, the 30-kDa movement protein (MP), and the CP. For TMV vulgare (i.e., strain U1), the full genome sequence was published in 1982 [225].

Particles of distinct tobamovirus species differ slightly in their properties. The virions have a buoyant density in CsCl of 1.307—1.325 g cm^−3^ and a sedimentation coefficient of 187 S to 212 S [211,212], and references in Table S1. The isoelectric point (pI) varies between 3.7 and 4.64, although the values determined experimentally may be slightly lowered by PEG remnants on the virions after PEG-mediated precipitation, i.e., from pH 3.7 to pH 3.38 in the case of pepper mild mottle virus [226]. Tobamoviruses have a thermal inactivation point (10 min) of 90 °C and survive in plant sap for many years; up to 50 years have been reported [227].

At present, the genus *Tobamovirus* is one of seven genera in the family *Virgaviridae* and comprises 37 accepted species plus two tentative species not yet classified [211]; https://ictv.global/report/chapter/virgaviridae/virgaviridae/tobamovirus. TMV represents the type member. Particles of at least 31 tobamovirus species have been purified from infected plant material (Table S1) by various methods, often based on PEG precipitation in combination with other techniques. Tobamoviruses can be classified into at least three subgroups based on the amino acid composition of their CPs, which correlates with serological specificity, CP amino acid primary structure, and the host from which the viruses were originally isolated [109,110,228,229]. Further differences between tobamovirus groups exist in the location of their origins of assembly (OAs) in the RNA and the separation or potential partial overlap of the open reading frames (ORFs) of the MP and the CP and/or the 183-kDa protein and the MP [110,211,221,230]. A classification of tobamoviruses on the basis of these optionally overlapping consecutive ORFs results in four subgroups [231].

Tobamoviruses have a broad host range that includes many crops and ornamental plants such as tobacco, tomato, cucumber, and orchids and cause major economic damage with, in some cases, no viable measures and regulations to contain the pathogens until the present. This is, for instance, currently the case for the tomato brown rugose fruit virus pandemic, with the virus detected initially in 2015 in Jordan now threatening tomato cultivation worldwide with no end in sight [97,221,232,233,234,235,236,237]. Another prominent example is the cucumber green mottle mosaic virus infesting different field-, tunnel-, or greenhouse-grown cucurbit crops in many countries after its rapid spread throughout the last decade, due to efficient transmission via commercially dispersed seed stocks discovered only recently and necessary adaptations of the international disease management standards still ongoing [233,238,239].

## 2. Techniques for Virion Enrichment and Storage, One by One

The extraordinary stability of tobamoviruses, exemplified above and detailed further below (in Section 2.6), is exploited to separate virus particles from plant components. Prior to purification, it is common practice to freeze infected plant material either at −20 °C or in liquid nitrogen [100,105,240,241]. The integrity of the virus particles is not obviously hampered by these procedures [242], which allow to separate harvest from purification temporally and/or spatially, at least for experiments not relying on highest-quality, evenly suspended virions (see Section 3.1 and [25]). Likewise, freshly infected plant tissues may be used as a starting material [243].

Initial strategies for virus purification from fresh or stored leaves, or plant sap in some cases, were developed in the 1930s (see Section 1.3.1). Since then, non-viral components from the plant homogenates are typically depleted in clarification steps, e.g., by adsorption, filtration, salt precipitation, heating, emulsification with chloroform and/or butanol, and sedimentation by centrifugation. Subsequent concentration and further purification of virions can be achieved by their precipitation, e.g., by salt or acid, and via various centrifugation techniques (see below and Table S1 with references therein). For higher degrees of purity, various techniques were combined serially and/or several cycles carried out repeatedly during the first decades of tobamovirus research. Alternatively, chromatography, e.g., in a matrix of controlled-pore glass beads, can be used to finalize the preparation (see Section 2.5, Table 1, Table S1). Since the introduction of PEG precipitation for bionanoparticle enrichment in the 1960s [205,244], PEG-based procedures have become the preferred standard methods for tobamovirus purification (see Section 1.3.4, Section 2.3 and Section 2.4.4). Their advantages are a high particle yield, the possibility to adjust precipitation conditions to specific virion properties with sufficient selectivity to remove significant amounts of plant contaminants, and relatively simple handling.

### 2.1. Grinding, Blending, and More: Plant Disruption

All purification strategies start with a homogenization step in order to disrupt plant tissue and cells and make the virus particles accessible. At this point, however, their precipitation into insoluble aggregates has to be prevented. If—primarily due to the release of vacuolar contents—the pH in the homogenate drops close to the viral pI (as an example, pI of 3.3 for TMV [245,246]), the virus particles might be lost due to aggregation. Therefore, homogenization is most commonly performed in sufficiently concentrated phosphate buffer at a pH of around 7. In their early work, Bawden and Pirie [247] developed an extraction protocol for TMV by freezing and thawing plant tissue in pure water, with the aggregation of TMV counteracted by a large liquid volume and repeated neutralization when necessary, as indicated by pH measurements.

For mechanical disruption of the plant material, different mincing and blender-type devices have been employed, including domestic meat mincers, Waring blenders, or smoothie makers [69,241,248]. The process may lead to a substantial production of frictional heat, so that cold disruption or intermitted mixing may be advantageous [243]. Grinding with a mortar and pestle [100] with the optional addition of an abrasive generates less heat but seems of lower importance for successful purification of tobamoviruses compared to virus members in other genera because of the nanotubes’ high stability. Notably, though, for natural TVCV and especially the protein A domain-fashioned TVCV_PA_, mechanical damage during the early purification steps was obvious upon use of standard TMV isolation protocols [181,248]. For this reason, we developed an initial version of a gentle PEG precipitate resolubilization method in inverse PEG-sucrose gradients [3], which is the starting point for our novel iodixanol-based method described below (Section 4). A systematic comparison of the particle sensitivities of distinct virus members of the Tobamovirus genus versus shear forces might be worth testing, as simple predictions from structural models seem not yet possible.

In any case, supplementation of homogenization buffers with protease inhibitors, commonly used in the biochemical purification of various molecules and supramolecular complexes from plants, is not a necessary standard for tobamovirus isolation. Even the mechanically more sensitive TVCV_PA_, which exhibits some CP degradation during purification and over time, did not profit from supplementation with the different protease inhibitors added to the extraction media [3,248]. This indicates that the mechanical sensitivity of native and especially engineered tobamovirus particles against either rupture or fragmentation of protruding heterologous CP portions may be an issue, but protease sensitivity usually is not.

### 2.2. Getting Rid of Plant Stuff: Clarification

Crude extracts can be used directly for the purification of tobamovirus particles by one of several methods (see Section 2.3, Section 2.4 and Section 2.5), but usually homogenates are first clarified by filtration through muslin, Miracloth, cheesecloth, gauze, or similar [240]. This step eliminates a major portion of larger plant components, e.g., unbroken tissue segments, leaf ribs, fibers, and cell wall fragments. As an alternative or in addition, filtration through a layer of adsorbents like Celite^®^ (a diatom silica preparation) or Bentonite (a less defined clay mineral primarily silica and a considerable amount of alumina) will further facilitate clearance of plant materials [249]. Charcoal may also be applied [23]. A more comprehensive view is presented in Section 2.5.2, as this clarification procedure can be regarded as a form of chromatography. Clarification of crude extracts is also achieved by a first low-speed centrifugation, which will be described later in detail (Section 2.4).

Since the structure of TMV particles is not affected by either freezing or heating up to 50 °C for about two hours [249] or about 90 °C for ten min in solution [250], these procedures can be used to precipitate plant components that may be further separated by a low-speed centrifugation step. K.-W. Mundry [243] described a method for TMV purification by a combination of repeated cycles of freezing and subsequent heat treatment at 60 °C, respectively. However, heat incubation alone at 50 to 55 °C can suffice to remove unwanted plant components from pre-filtered TMV-containing homogenates, with the heat-precipitated compounds sedimented by centrifugation thereafter [240,249].

A substantial amount of plant contaminants can be removed by incubation with n-butanol and/or chloroform, causing both precipitation of the unwanted material and extraction of chlorophyll into the organic phase simultaneously, while the virus particles will remain in the aqueous phase. This procedure is typically followed by centrifugation as well, to separate the precipitate and the organic phase from the TMV particles [205,206].

### 2.3. Precipitation of Virus Particles

Since the early days of tobamovirus purification, antichaotropic salts of the Hofmeister series, like ammonium sulfate, have been successfully used to precipitate tobamovirus particles from cleared extracts or homogenates [69,199,200,240]. If necessary, the salt-mediated precipitation is repeated to increase yield and purity. If TMV is already enriched, a slow increase in the ammonium sulfate concentration leads to the assembly of TMV into small paracrystalline needles of about 0.02 mm length [251] (Figure 1E).

An efficient and, to some extent, selective precipitation of virions can be achieved by the addition of the water-soluble polymer PEG (typically used with an average molecular weight of 6000 or 8000) in concentrations above a threshold specific for the respective virus type. Precipitation is promoted by the presence of at least 2% (*w*/*v*, final concentration) monovalent cations, usually NaCl. The method was introduced for plant virus purification by T.T. Hebert in 1963 [244] and optimized for TMV by R. Leberman [252] and by G.V. Gooding and Hebert [205] soon afterwards, with the detailed description of a slightly modified protocol by S. Chapman in 1998 [206]. As their depletion-induced precipitation depends on the shape, charge, and size of the particles (worked out briefly in [3]), conditions may be found that separate virions well from the majority of accessory plant compounds. Anisotropic, i.e., elongated virus particles aggregate and re-dissolve at lower PEG concentrations than spherical protein complexes, CP oligomers, or soluble proteins [253,254]. For tobamovirus purification, PEG precipitation has thus become a predominant method (see Section 1.3.4). In order to increase the yield of virus particles, repeated rounds of PEG precipitation may be performed.

A major advantage of purification protocols using precipitation steps for the enrichment of viruses is the dispensability of ultracentrifugation because the aggregated virions can be sedimented efficiently by low *g*-forces. This does not only reduce the necessary instrumentation and effort but also allows for the processing of larger amounts of virion-containing plant extracts. The structural integrity of TMV particles collected via PEG precipitation is demonstrated by a corresponding high-resolution structure determined by electron cryo-microscopy and image reconstruction [104].

### 2.4. Centrifugation

Centrifugation stands as a cornerstone technique to separate and concentrate tobamoviruses from the cellular debris of plant tissue. It capitalizes on differences in size, density, and shape to achieve purification and enrichment. Ultracentrifuges, capable of generating immensely high centrifugal forces ranging from 100,000× *g* to 1,000,000× *g,* are instrumental in removing sediment particles with sedimentation coefficients in the range of 10 to 10^5^ S. For instance, TMV, with a sedimentation coefficient of approximately *s* = 200 S or 200 × 10^−13^ s in aqueous solution [53,255], can sediment at a velocity of around 10 cm/h in a gravitational field of *RCF* = 140,000× *g* (for theory, see [256]).

**Figure 2:**
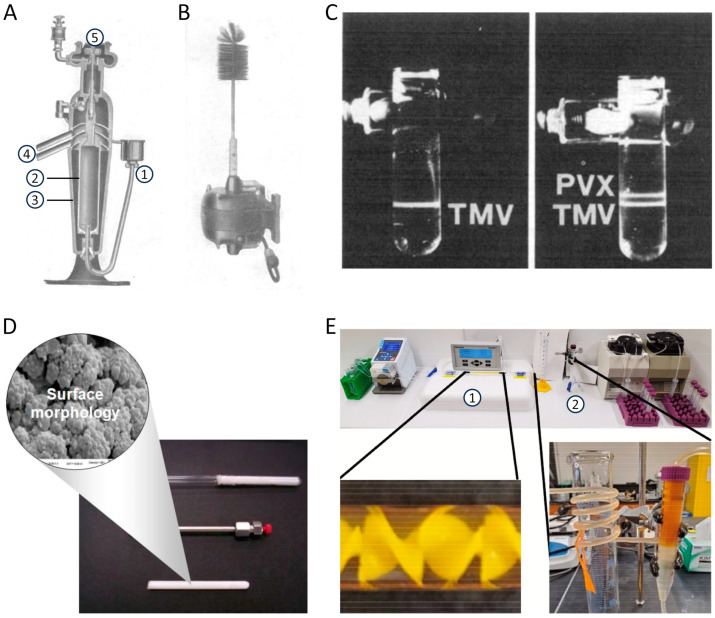
Laboratory instruments for virus purification. (**A**) Supercentrifuges were run in a continuous mode to pellet virus or virus precipitates. The rotor (2) within a chassis (3) is connected to a drive (5). Feed liquid (1) enters the rotor and separates into heavy and light fractions, which leave the centrifuge through separate outlets (4). (**B**) Glassware was cleaned, for example, with electrically driven test tube and flask washing machines. A and B were reproduced from Prucha and Tanner 1920 [286] with minor changes. Copyright 2024 by Copyright Clearance Center, Inc., Denvers, U.S. on behalf of the American Society for Microbiology Journals, Washington, D.C., U.S. (**C**) Later, viruses were separated by density gradient centrifugation, for example, with isopycnic banding in Nycodenz^®^ gradients. Reproduced from Gugerli et al., 1984 [280], with minor changes. Copyright 2024 by Copyright Clearance Center, Denvers, U.S. on behalf of Elsevier Science & Technology Journals, Amsterdam, Netherlands. (**D**) For the past 20 years, monolithic materials have been evaluated for virus purification by chromatography. In monolithic materials, viruses can easily access active sites on surfaces by convection, which allows increased flow rates. Reproduced from Arrua et al., 2009 [287], with minor changes. This work is made available under the terms of the Creative Commons Attribution-NonCommercial 3.0 Unported License, http://creativecommons.org/licenses/by/3.0/, accessed on 7 March 2024. (**E**) For the continuous purification of viruses, aqueous two-phase systems are currently being explored. A virus solution and both phases are combined and mixed in the first part of the instrument (marked with 1 and shown in the lower left inset). The two phases are then separated in a second part of the instrument (marked with 2 and shown in the lower right inset). Adapted from Turpeinen et al., 2021 [288] with minor changes. Copyright 2024 by Copyright Clearance Center, Denvers, U.S. on behalf of Elsevier Science & Technology Journals (Amsterdam, The Netherlands).

Ultracentrifugation finds application both analytically to determine the sedimentation coefficients of a particle and preparatively to separate the virus from cellular debris and/or to concentrate virus preparations. Preparative ultracentrifugation may utilize different sedimentation coefficients (‘differential sedimentation’), different densities (‘isopycnic density gradient centrifugation’), or a combination thereof (‘zonal density gradient centrifugation’) known to separate different types of particles. To scale up preparative ultracentrifugation, rotor designs and appropriate equipment enable continuous ultracentrifugation (Figure 2A,B) with or without density gradient material. 

#### 2.4.1. Differential Sedimentation

The initial documentation of tobamovirus preparation through differential sedimentation dates back to 1927 [257], marking a pivotal moment in virology. Notably, at that time, the true nature of TMV remained elusive. The sedimentation of the virus was only achieved after heat coagulation of the virus. Subsequent analytical determinations of sedimentation coefficients [53,255] paved the way for the widespread use of ultracentrifugation to purify and characterize the virus from plant sap [258,259,260,261,262,263]. Early protocols involved multiple high-speed centrifugations to pellet the virus, interspersed with low-speed centrifugations to separate the virus into the supernatant (‘differential centrifugation’). However, these early preparations often suffered from virion aggregation that could not be resolved. Improved buffer conditions for preparation (0.1 M phosphate) and storage (0.01 M phosphate) of the virions were subsequently elucidated [100,264]. Differential centrifugation was applied for the isolation of many tobamoviruses (e.g., [25]; Table S1). Aggregation during preparation for TEM analytics, however, remained a concern, challenging the perception of virus monodispersity in solution [265]. Tobamoviruses have also been purified by several adaptations of differential centrifugation, such as parallel chloroform extraction ([266], and Table S1) and high-speed centrifugation through sucrose cushions ([267,268]; Table S1), which present potential alternatives to labor-intensive density gradient centrifugation [269].

#### 2.4.2. Continuous Ultracentrifugation

Despite centrifugation traditionally being viewed as limited in terms of scalability, advancements in rotor design have facilitated a degree of scalability that allows for the preparation of larger quantities of viral particles. This approach was first documented in 1942 [89], when it enabled the preparation of up to 15 g of TMV within ten hours. Given the substantial yield achieved through this method, it warrants reconsideration for applications necessitating high quantities of virus. Furthermore, continuous ultracentrifugation has been adapted to gradient separations for human influenza B and herpes simplex virus [270]. However, to the best of the authors’ knowledge, the purification of tobamoviruses with continuous density gradient centrifugation has not been reported.

#### 2.4.3. Density Gradient Centrifugation

Density gradient centrifugation of plant viruses has been extensively reviewed [271]. In one variant, known as rate zonal gradient centrifugation, sucrose gradients typically ranging from 10 to 40% (corresponding to densities between 1.04 and 1.18 g/mL) are prepared in a centrifugation tube [272,273]. Solutions containing the virus, with a density of about 1.3 g/mL in the case of TMV, are layered on top of the gradient, and the virus particles are centrifuged into the gradient for 1–3 h, depending on centrifugal force. As the density of the gradient material is lower than that of the virus, the duration of centrifugation must be carefully controlled. Methodological nuances include variations in the average sucrose density, gradient steepness, and virus loading quantities [274,275]. Although the method was developed long ago, it remains prevalent in contemporary laboratories [107,191,276]. Notably, adjustments to the gradient may be necessary for variable particle lengths [277], as zonal gradient centrifugation depends not only on density but also on virus shape and size, enabling the separation of particles that have been disassembled to different degrees [278].

In contrast, isopycnic gradient centrifugation employs a density gradient medium matching the density of the virus particles. During centrifugation, the virus migrates until its density aligns with that of the medium, forming a distinct band (Figure 2C). While for rate-zonal gradient centrifugation, the gradient is typically pre-formed, during isopycnic gradient centrifugation, the gradient is formed during centrifugation, requiring several hours to reach equilibrium. TMV, for example, achieves equilibrium in gradients formed by 32% CsCl only after approximately 16 h [279]. Alternative alkali halides yielded comparable results to CsCl [279]. To address CsCl’s high ionic strength and potential for virus aggregation, non-ionic and non-chaotropic density gradient media such as Metrizamide and Nycodenz^®^ have been explored [280,281]. Both compounds share a tri-iodinated benzamido group and multiple hydroxyl groups as common structural elements [282,283]. Metrizamide facilitated shallower gradients and improved the separation of closely related tobamoviruses [281]. Nycodenz^®^ allowed the separation of potato virus X and TMV (Figure 2C) but poses challenges in its removal from the virus particle preparations after centrifugation, thus making it attractive mainly for viruses unstable in CsCl or Metrizamide [280].

Colloidal silica presents a compelling alternative to traditional density gradient methods, offering reduced run times and mitigated osmolarities [284]. Isodensity banding with colloidal silica can be achieved in less than 1 h, with viruses banding at much lower apparent densities compared to CsCl, owing to their exclusion of colloidal silica from the surrounding medium. This exclusion effect, contingent on size and form, enables separation based not only on density but also on the exclusion phenomenon. However, silica adherence to viral particles necessitates precipitation to remove silica from the virus after centrifugation, a challenge addressed by coating silica particles with polyvinylpyrrolidone. This innovation has led to the development of Percoll, a widely used gradient material for cell separation [285]. While there is currently no reported data on TMV purification using Percoll, its potential merits further exploration in virological research.

#### 2.4.4. Solubility Gradient Centrifugation

A refined isolation method combines precipitation with density gradient centrifugation, resulting in the resolubilization of a virus precipitate upon its migration along a gradient and the banding of the newly dispersed virions in a relatively narrow zone of the tube [1,2]. This method involves centrifugation of PEG-precipitated virus from plant raw homogenates through a reverse concentration gradient of PEG 6000 stabilized in a positive sucrose gradient. The solubility concentration, determining when virus particles resolubilize, relies on their surface/volume ratio and charge characteristics and is influenced by pH and ionic strength within the solubility gradient. This technique has been tested successfully for TMV and plant viruses from various genera [2]. Recently, it was adapted to gently isolate the tobamoviruses TVCV and its mutant TVCV_PA_ [3], resulting in a well-preserved particle length compared to that of virions obtained via methods involving PEG precipitation and differential centrifugation in later stages of the purification process, i.e., after several treatments exerting mechanical stress on the viral rods [181,248]; see also Section 2.1.

### 2.5. Chromatography

Chromatography is employed in two distinct modes for tobamovirus purification. In the flow-through mode, the virus traverses the chromatographic matrix while contaminants are retained. This mode is frequently applied in the initial stages of virus isolation to clarify plant saps. In the bind/elute mode, the virus is captured selectively under specific binding conditions by the column medium, while contaminants are not. Subsequent elution facilitates direct virus purification (Table 1).

#### 2.5.1. Flow-Through Chromatography

Initially, chromatography found application in the purification of tobamoviruses through its capacity to bind plant components to the chromatographic material, thereby clarifying the virus-containing extracts. For a comparison of flow-through chromatography with other clarification methods, please refer to Section 2.2. One of the earliest materials utilized for flow-through chromatography was Celite, derived from pulverized diatom shells and predominantly composed of amorphous silicon dioxide [251]. Other materials such as Bentonite, a complex aluminum silicate, charcoal, and DEAE-Cellulose were similarly employed [289,290]. These materials effectively remove plant components such as ribosomes, Rubisco, or phytoferritin from extracts containing the virus, thereby facilitating the purification of several tobamoviruses (Table S1 and [25,291]).

The use of paper chromatography marked an interesting development in TMV purification by flow-through chromatography, employing a simple yet effective principle where the virus could migrate through paper at pH values of 4.5 or higher, leaving behind plant components like chlorophyll at the application site [292]. In a notable modification to this technique, an electric field was applied during paper chromatography at pH 7, resulting in the separation of plant components and a distinct fraction containing the virus, demonstrating an early instance of electrophoretic mobility enhancing chromatographic separation [293].

Chromatography, which is typically effective for protein separation, faces limitations with tobamoviruses due to their size. Standard methods often use porous beads, but tobamoviruses’ larger size prevents efficient diffusion into these pores, reducing interaction with the stationary phase and complicating isolation efforts. Still, methods based on chromatography have been developed for either the clarification or purification of the virus.

Size exclusion chromatography, utilizing porous particles, differentiates molecules based on size. Small molecules and buffer components enter the pores and traverse the column slowly, whereas larger entities like viruses bypass these pores, enabling their faster movement. This method effectively acts as a ‘flow through’ mode for viruses. Advances in isolation techniques have introduced materials such as controlled pore glass [294] or microgranular Spheron gels [295] for efficient separation of TMV and other plant viruses through size exclusion column chromatography ([296,297]; Table S1).

#### 2.5.2. Bind/Elute Chromatography

Bind/elute chromatography is the main chromatographic method employed for virus purification (Table 1). Early chromatography media for tobamovirus enrichment included cellulose derivatives [298,299,300]. With the advent of monolithic columns [301,302], tobamoviruses found compatibility with supports fashioned as continuous homogeneous phases [301,303,304]. Unlike individual particles, monolithic columns (Figure 2D) feature large pores resembling channels throughout the entire length of the column, rendering the entire stationary surface accessible to tobamoviruses. Convective Interaction Media (CIM) monolithic materials with anion-exchanging capabilities have been particularly successful in tobamovirus studies (Table 1).

Though chromatography is probably one of the most important purification methods for bioproduction, it is still not commonly applied in routine labs, and no state-of-the-art chromatography material has been accepted. Density gradient ultracentrifugation is still the method of choice when high purity of the virus is required. However, promising materials such as monolithic columns are becoming more and more recognized.

**Table 1:** **Chromatographic media and conditions for the purification of tobamoviruses via the bind/elute mode.** Arrows indicate gradients, with starting conditions in the line above the arrow and final conditions on the right side of the arrow.

Type	Stationary Phase	Mobile Phase	Comment	Citation
Cation exchange	Carboxymethyl-cellulose	5 mM Citric acid pH 3 → 50 mM Na_2_HPO_4_/0.1 M NaCl pH 9.05	--	[298]
Cation exchange/ Ca^2+^ affinity	Hydroxyapatite	0.001 M Phosphate pH 6.8 → 0.3 M Phosphate pH 6.8	Used for the separation of partially and completely in vitro reconstituted particles	[305]
Anion exchange	Cellulose modified with epichlorhydrin and triethanolamine	0.01 M Phosphate pH 7 → 0.01 M Phosphate/1 M NaCl pH 7	Used for the separation of in vitro reconstituted particles	[299]
Anion exchange	DEAE-cellulose	0.01 M Tris-HCl pH 7.3 → 0.8 M Tris-HCl pH 7.3	--	[300]
Anion exchange	CIM—QA	1. 20 mM NaOAc pH 5.5 2. 20 mM NaOAc/1.5 M NaCl pH 5.5	Yields 70–90% (enzyme-linked immunosorbent assay) Tomato mosaic virus only	[303]
Anion exchange	CIM—QA + CIM—DEAE	Various; best performing with CIM QA: 1. 20 mM NaOAc pH 5.5 2. 20 mM NaOAc/0.45 M NaCl pH 5.5 3. 20 mM NaOAc/1.5 M NaCl pH 5.5	Highest yield with CIM—QA Up to 98% yield, on average 75% (enzyme-linked immunosorbent assay) Allows purification from clarified extract within one step; tomato mosaic virus only	[304]
Anion exchange	CIM—QA + CIM—DEAE	20 mM NaOAc pH 5.5 → 20 mM NaOAc/1 M NaCl pH 5.5	Separation of different viruses from their mixtures	[301]
Various	Chitin	1. 0.01 M Tris-HCl pH 6.8 2. Water → 0.5 M K_2_HPO_4_	--	[306]
Various	Methylated albumin on kieselgur (MAK)	0.05 M Phosphate/0.2 M NaCl → 0.05 M Phosphate/1 M NaCl	Separation of RNA, TMV CP, and TMV particles	[307]
Size exclusion	Spheron (hydroxyalkyl methacrylate gel)	0.05 M Tris-HCl pH 7.5	Various particle sizes and exclusion limits were tested Yield about 90% (method not specified)	[295]
Size exclusion	Controlled-pore glass	0.05 M Phosphate pH 7	Recovery close to 100% (spectrophotometry) Comparison with sucrose gradient centrifugation Recovery of centrifugation: 80% (spectrophotometry) Comparison of different viruses	[294]

### 2.6. Final Steps

#### 2.6.1. Ultrafiltration

Ultrafiltration has been utilized primarily to concentrate tobamoviruses and for buffer exchange, especially after the functionalization of the virus surface [107].

#### 2.6.2. Lyophilization and Storage of Virus Preparations

The stability of viruses during storage is crucial for their use in research and biotechnological applications. Tobamoviruses, particularly TMV, have been studied extensively for their robustness and persistence under a variety of storage conditions. This review delves into the seminal works that have contributed to the methodologies for storing tobamoviruses, emphasizing the evolution of liquid and dry storage techniques.

Historically, the liquid storage of tobamoviruses has revealed their extraordinary stability. A pivotal observation was that TMV could remain stable for up to 50 years in liquid formulations, a testament to its robust nature [227]. Recently, an over-40-year-old preparation stored in a cold room was analyzed and still contained predominantly 300 nm long TMV rods [197]. This longevity in liquid medium not only underscores the virions’ structural stability, but also their applicability in long-term studies without significant loss of infectivity, and in technical or biomedical uses demanding robust performance.

One study meticulously investigated the stability of TMV at different pH levels and ionic strengths at an elevated temperature of 68 °C [308]. This investigation revealed that a minimum pH of 6.8 (the lowest pH value tested), combined with a 0.2 M phosphate buffer (the highest concentration tested), conferred the highest stability to the virus. Another study showed that the ultracentrifugation properties and infectivity of TMV remained stable at 4 °C for up to 28 weeks, both in water and 0.02 M phosphate buffer at pH 7.5 [309]. In buffered solution at pH 4, the virus retained its infectivity for up to twelve years at 4 °C (the maximum duration tested) [310]. Alternatives to aqueous solutions were also investigated [311]. The circular dichroism (CD) spectrum of TMV stored at room temperature in ethylammonium mesylate, a protic ionic liquid, was stable for up to four months (the maximum reported), while it decreased within three weeks when stored in 10 mM phosphate buffer pH 7.2.

The transition to dry storage methods presented new avenues for preserving tobamoviruses, with techniques such as freezing and lyophilization (freeze-drying) being explored. No changes in ultracentrifugation properties were observed when the virus samples were frozen in water or 0.1 M sodium acetate pH 6 [242], indicating that freezing did not adversely affect the structural integrity of the virus. When frozen in phosphate buffer, slight changes in ultracentrifugation properties were noticed in one study, but without effects on infectivity [309]. These findings have been pivotal in establishing freezing as a viable method for the long-term storage of tobamoviruses.

The drying of viruses presents unique challenges, notably the risk of reduced infectivity. Drying TMV with P_2_O_5_ decreased its infectivity to 50% or less, regardless of whether the sample had been frozen before drying or whether the pH of the solution was neutral or acidic. However, drying with Na_2_SO_4_/Na_2_SO_4_·10 H_2_O or saturated ammonium sulfate retained the infectivity of TMV [56]. This indicates a certain level of stability during the drying process but also underscores a propensity for infectivity loss. Freeze-drying was explored as a method to retain the stability of the virus. When dried under very low pressures (<0.1 mm Hg, corresponding to 0.13 mbar), only a few particles broke [312]. However, significant particle breakage occurred at higher pressures or if the sample had thawed before water sublimation, suggesting that conditions during freeze-drying critically influence virus integrity. A systematic optimization of freeze-drying conditions for tobamoviruses identified specific temperature and pressure profiles as well as cryoprotectants that sustained up to 89% infectivity [313]. This work significantly advanced the understanding of freeze-drying as a viable method for the long-term storage of tobamoviruses, offering insights into the delicate balance required to maintain viral integrity during the lyophilization process.

The historical advancements in the storage of tobamoviruses, from liquid formulations to dry storage methods, have provided a foundation for current practices in virology research and biotechnology. The remarkable stability of tobamoviruses, particularly TMV, under various conditions has not only facilitated their use in scientific studies, but also highlighted the importance of specific storage conditions to preserve viral infectivity and integrity. As research continues, the insights gained from historical studies will undoubtedly contribute to further innovations in the storage and utilization of viruses for a range of applications.

## 3. Combined Recipes: Stirred, Shaken, and Poured

### 3.1. Adapting Purification Strategies to Specific Needs: Combined Protocols and a Few Pitfalls to Keep in Mind

Purification strategies generally have to be adapted to the needs and requirements of the intended follow-up experiments with the isolated virus particles. Some aspects to be considered, as well as an instructive example highlighting the relevance of optimized purification protocols, are summarized in this section.

The early methods used for the purification of tobamovirus particles were based primarily on precipitation and centrifugation and aimed at sample material for understanding the identity of the mosaic-inducing agent, for the identification of virus species, or for elucidating the underlying structures by X-ray diffraction (Section 1.3.1 and Section 1.3.2 and Table S1). Around thirty years later, PEG precipitation was established in virus purification procedures [205,206,244,252] including protocols for tobamoviruses, which have evolved into the standard methods due to several advantages (Section 2.3). They yield virus particles of sufficient quality for most subsequent applications, though not for every purpose. It should be borne in mind that the properties of the virions may be altered by PEG residues adhering to the surface, as indicated by shifted pI [226] and high-resolution EM (according to personal communication with experts). Additionally, it should be remembered that divalent cations such as Ca^2+^ and Mg^2+^ strongly favor end-to-end aggregation of virions, which might interfere with subsequent applications. This phenomenon can be avoided by adding EDTA and by replacing the divalent ions with Na^+^ in the first steps of the purification process [314,315,316].

PEG precipitation is often combined with other methods for multi-stage purification processes that are best suited for the intended downstream applications of the VNPs, such as antiserum production via immunization of rabbits (Table S1 and [317]). The combination of a PEG-based isolation protocol with zonal sucrose density gradient centrifugation, or centrifugation through a sucrose cushion, respectively, may be used to fractionate tobamoviral particles according to their aspect ratio in order to examine and narrow down their size distribution towards relatively uniform preparations, e.g., [217], particles treated according to Supplementary Reference [48] within Table S1, or [318]. Such fractionation according to the particle size (corresponding with its sedimentation coefficient, see Section 2.4.3) can also be achieved by successive centrifugation steps of the pre-cleared homogenate, including separation in a density gradient, to recover the original particle population without PEG precipitation and resuspension [267] (see Table S1). From nanorod fractions of different average lengths obtained through such a protocol, the encapsidated genomic and subgenomic tobamoviral RNAs were isolated and further characterized, i.e., according to their size, coding capacity as revealed by in vitro and in vivo translation, or localization of the OA sequence [217,267,318].

Avoidance of PEG precipitation has also been a rationale in even simpler purification protocols that aim at well-dispersed, high-quality virions. Under carefully adjusted pH and ionic strength conditions, preparations of near-uniform particles are also accessible by well-controlled differential centrifugation of cleared plant crude extract alone. In this case, full-length particles sedimented prior to the spin-down of shorter ones—which are removed with the supernatants—undergo a number of successive centrifugation cycles, each with extensive resolubilization of the pellets overnight and mechanical resuspension [100] and Table S1. The excellent dispersion and purity of such virion preparations have made them the first choice for certain subsequent applications, e.g., for biophysical and structural investigations [25].

The work of Kreibig and Wetter [25] is an informative example of how the selection of a specific method for particle purification and its precise implementation may determine the outcome of subsequent experiments. As described above (Section 1.4), the authors purified virions of six distinct tobamovirus species from infected plant material to study their assembly into different liquid-crystalline phases in vitro by optical diffraction. However, the successful generation of the starting materials, the iridescent gels formed by the virions, depended strikingly on the virus isolation protocol. Three different purification methods were tested comparatively, and only the one according to Boedetker and Simons [100] (see Table S1 for further details) proved reliably successful after the necessary numbers and sedimentation parameters of the differential centrifugation cycles had been optimized thoroughly. The particle purity required for the optimal formation of iridescent gels showing diffraction colors was examined in much detail. It was found that the highest purity was achieved through four cycles of centrifugation, whereas the ability of the virions to form the desired gels was optimal after three cycles. This indicated monodispersity—compromised by excessive repetitive resuspension—as an important factor for crystallization. In addition, the study found that the storage conditions of the plant material before virion isolation were also crucial for the success of the follow-up experiments. Only plant material frozen for short (overnight) periods led to a reliable applicability of the isolated particles in optical diffraction tests, whereas virions obtained from plant tissues after long-term storage (i.e., frozen for one to five years) yielded ‘poor results’ [25].

### 3.2. Methods Worth Being Explored in Future Work

While this review has explored various alternative strategies tested and implemented for tobamovirus purification, it is worth looking at a few innovative techniques that, to our knowledge, seem not to play considerable roles in the enrichment of TMV and related plant viral particles as yet.

Asymmetrical flow field-flow fractionation (AF4) is one such technology [319]. AF4 instruments employ a channel flow in combination with a cross flow, the latter directed towards a permeable ultrafiltration membrane with a molecular weight cut-off (MWCO) chosen according to the desired particles. Sample components with small diffusion coefficients and higher hydrodynamic diameters travel closer to the membrane along the channel flow, while those with higher diffusion coefficients and lower hydrodynamic diameters travel faster due to the parabolic shape of the laminar channel flow. This method, not requiring virus aggregation or interaction with a stationary phase, offers gentle purification leading to homogeneous particles. Although primarily applied analytically, preparative approaches are also described [320].

Aqueous two-phase systems (ATPS) represent another technique utilizing the separation of two aqueous systems into two phases for liquid-liquid extraction. The first experiments with TMV in aqueous two-phase systems were described [321], and continuous ATPS has enabled the purification of mammalian viral particles (Figure 2E) with high contaminant partitioning capacity [288]. Continuous purification methods are especially interesting because they allow facile scale-up.

Finally, despite the most frequent applications in the final purification steps involving discardable devices, membrane chromatography is increasingly valued for virus manufacturing due to its scalability and may also be used in different flow-through modes depending on the type of device [322]. Membrane chromatography, compared to traditional resin column chromatography, reduces pressure drop and mass transfer resistance [323,324]. This is similar to the advantages of the interconnected channels in monolithic columns, which—apart from uses in bind/elute chromatography (see Section 2.5.2)—may also be integrated into advanced radial flow devices for upscaling, thereby evading problems with the mechanical instability of disk-shaped monolithic CIM media or pressure increase in elongated columns [322]. An instructive comparison between monolithic, membrane, and conventional chromatography may be found online as an application guide [325].

Depending on the specific need, such new methods for tobamovirus purification to yield highly homogenous particle suspensions or allow scale-up, respectively, will be required to be tested and adapted. The pioneering fundamental research on and with tobamoviruses in many labs worldwide brought forth a fast, straightforward method development in the mid-20th century, but only a limited number of main strategies have been followed since then. PEG precipitation remains the most widely adopted method for TMV purification in contemporary laboratories, in combination with a number of centrifugation modes. These strategies were rooted in the favorable virion properties described above, which give rise to efficient separation strategies for rod-shaped nucleoprotein colloids from most plant components with relatively low effort in comparison to purification protocols for individual proteins or small biomolecule oligomers. Nonetheless, it should be worth testing the abovementioned non-established methods in combination with high throughput-compatible equipment, as processed and in vitro-assembled non-infectious tobamovirus-based particles are attracting increasing attention as biogenic multivalent nanomaterials for many potential uses. Upscaling opportunities would expand the application prospects further.

## 4. The Established and the New—Back to the Future? A Case Study

### 4.1. Motivation and Challenge

#### 4.1.1. Exploring New Approaches for Engineered Tobamoviruses

To assess the potential benefits of historical methodologies for novel approaches involving engineered tobamoviruses, we conducted a case study with TVCV_PA_. This specific TVCV mutant features an extensive polypeptide with two *S. aureus* PA-domains (E and D, 133 amino acids) displayed on every CP subunit [181]. The size of these extensions is significant enough to be observed via TEM as a granular seam surrounding TVCV_PA_ virions (see below). The PA-domains retain their IgG binding ability when fused to the CP’s C-terminus via a long flexible linker ([GGGGS]_3_) [181]). TVCV_PA_ thus represents a rod-shaped nanoparticle with a predicted length of 317 nm, exhibiting immunosorbent properties and amenable to facile production through molecular farming. Previous studies have demonstrated its capacity to bind more than 500 IgGs per virion [3], enabling multiple applications in biotechnology, including IgG capture in pull-down assays or magnetic bead separation [248], enzyme immobilization, and most likely in biosensors [3]. With regard to the wide potential applications of TVCV_PA_, we considered it an intriguing candidate for developing a new VNP isolation technique, particularly for challenging, potentially more sensitive tobamovirus mutants in future studies.

#### 4.1.2. Challenges in Isolating TVCV_PA_

Traditional isolation methods for TVCV_PA_ yielded inconsistent results in terms of particle dimensions [181,248]. Despite various modifications tested in order to enhance the scalability and quality of the preparations, challenges persisted in achieving concentrated particles of the anticipated lengths and storage stability.

To address these issues, we aimed to develop a novel isolation technique tailored to the unique characteristics of TVCV_PA_. Initially, TVCV_PA_ was isolated from mechanically pressed leaf juice using chloroform extraction, double PEG precipitation, and low-speed centrifugation [181]. This method yielded approximately 3 g TVCV_PA_ kg^−1^. *Nicotiana benthamiana* leaves with an average virion length of 200–220 nm, mirroring the length of the TVCV wildtype isolated by the same procedure. These observed average lengths of both virion types did not align with the anticipated ones of 317 nm and 296 nm, respectively.

Modified protocols revealed the susceptibility of both TVCV and TVCV_PA_ (abbreviated ‘TVCV_(PA)_’ in the following, if both are addressed) virion integrity to mechanical stress: Isolated particles displayed notably increased average lengths (239 nm) when leaves were ground frozen in a mortar, compared to virions released from the tissues in a blender (217 nm) [248]. An iteration of the double PEG precipitation, integrating butanol extraction and ultracentrifugation for the ultimate virion collection, resulted in equally high yields of TVCV_PA_ (2.5 g TVCV_PA_ kg^−1^ *N. benthamiana* leaves) of high purity, as evidenced by a minimal background in SDS-PAGE and a median virion length of approximately 270 nm [3]. However, this length still deviated from the predicted one. Recently, this inconsistency was overcome by using inverse PEG sucrose solubility gradients, as introduced by Clark in 1970 [1]. In contrast to protocols relying on PEG precipitation/sedimentation, PEG solubility gradient-based isolation of TVCV_(PA)_ avoids the pelleting of virions at the tubes’ bottom. Virions are precipitated directly in the plant’s raw homogenate and pass the gradient up to the region with the highest PEG concentration allowing selective resolubilization, where they accumulate in a largely pure band. The early formation of the particle precipitate in the course of the treatments, and its gentle resuspension, probably mitigate TVCV_(PA)_’s susceptibility to mechanically induced shortening. This resulted in TVCV_(PA)_ particles exhibiting a length distribution closer to their predicted dimensions (largest classes of rod lengths: TVCV 280–300 nm; TVCV_PA_ 300–320 nm), albeit with a slightly reduced yield (1–2 g TVCV_(PA)_ kg^−1^ *N. benthamiana* leaves) compared to PEG-precipitation/sedimentation methods [3].

While the inverse PEG-sucrose solubility gradients yielded TVCV_PA_ and TVCV preparations with elevated purity and integrity, a drawback was that virions were distributed along the gradients quite broadly. Consequently, the harvest of virus-containing gradient fractions produced relatively high sample volumes and rather dilute samples, necessitating gradient fractionation and the testing of each fraction for both purity and virus concentration. To improve the isolation of TVCV_(PA)_ in regard to these aspects, we departed from the PEG-solubility gradient approach and instead developed a method based on density barriers and precipitate re-solvation in an advantageous separation medium, as described below. The detailed description of the method can be found in the supplemental information.

### 4.2. Strategy

#### 4.2.1. Integration of Traditional and Contemporary Techniques

Drawing inspiration from Clark und Lister [2], a hand-drawn sketch from Holger Jeske, whom this article is dedicated to, as well as from animal VLP isolations from plants by Thuenemann et al. [326,327] and Dennis et al. [328], we integrated elements from the classical PEG-precipitation technique introduced by Hebert [244] with contemporary non-ionic density gradient ultracentrifugation. In the new method, an initial PEG precipitation facilitated the processing of extract volumes that were impractical for direct application onto phase systems, as in our earlier work [3]. A subsequent phase system was designed to resolubilize the PEG precipitate, eliminate associated contaminants, concentrate the virus on a density cushion in an overnight ultracentrifugation, and yield preparations ready-to-use for different purposes.

#### 4.2.2. Rationalization of Density Barrier Phases

Iodixanol was selected as the density medium due to its high density, low viscosity, and biocompatible, inert, and uncharged characteristics [329]. Employing iodixanol, our primary objectives were to achieve two major improvements over the previous PEG solubility gradient-based isolation method: (i) simplify the gradient architecture to reduce the workload, and (ii) obtain a more concentrated yet clean and undegraded virus sample. We simplified the concept of ‘sample-on-top-of-phases’, with a number of increasingly dense phases from top to bottom [327,328], to a three-layer density barrier phase system. This rationalization to only three phases was possible by designing the phases’ densities according to the buoyant density of the viruses, which had to be determined first within the iodixanol compound. On this basis, specific functionalities and dimensions could be assigned to the three phases of the barrier system: the upper one was adjusted to a density slightly above that of the PEG-precipitated, resuspended sample to act as a loading support, filler, and pre-purification zone, which facilitates sample application and retains mainly very low-density constituents. The middle phase served as a retention phase, primarily for constituents with a buoyant density lower than that of the virus and other contaminants with low sedimentation velocity. The lower phase acted as a cushion, retaining and concentrating the virus without pelleting but allowing passage/removal of constituents with a higher density than the virus particles. Although all three phases were set up in the same buffer in our application, it is conceivable to prepare every phase with a different buffer constitution, imparting the entire phase system with advanced washing and buffer exchange capabilities. Even zones of considerably distinct composition and function might be added by placing them into the phase system via their density adjusted by iodixanol, as needed.

### 4.3. Results

#### 4.3.1. Buoyant Densities of TMV, TVCV, and TVCV_PA_ in Iodixanol and Phase Design

**Figure 3:**
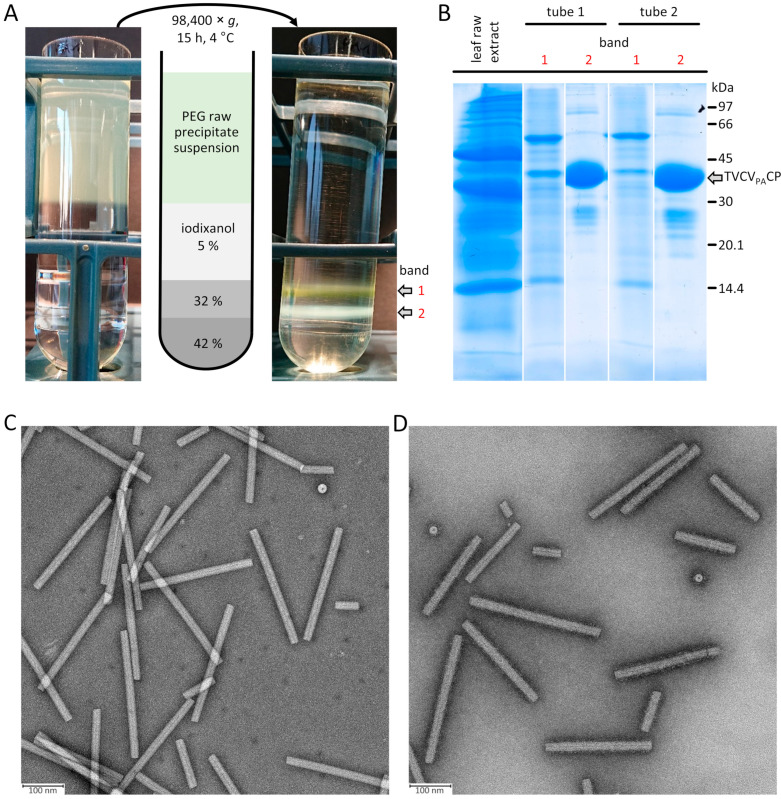
Isolation of TVCV and TVCV_PA_ using a novel three-layer density barrier phase system (5/32/42% (*w*/*v*) iodixanol) for virion purification from PEG precipitates of raw extracts. (**A**) Photograph and scheme (middle) depicting a three-layer density barrier phase system loaded with a resuspended PEG precipitate (17.5 mL TVCV_PA_-containing sample per SW 32 Ti ultracentrifugation tube). One of the two corresponding tubes (tube 1) was photographed before (left) and after (right) centrifugation (4 °C, 15 h, 24,000 rpm [rmax 98,381× *g*]). Visible bands formed upon centrifugation in the 32% iodixanol-containing retention phase (arrows 1 and 2) were sampled by puncturing the tube’s wall with a syringe and needle. Band 1 (arrows 1) was partially aspirated (300 µL) for later analysis by SDS-PAGE (sample 1 in subfigure (**B**)), and band 2 (arrows 2) was taken completely (1.3 mL per twin tube, sample 2 in subfigure (**B**)). (**B**) SDS-PAGE (15% polyacrylamide) of samples taken from leaf raw extract before PEG precipitation, and samples 1 and 2 taken from tube 1 (shown in (**A**)) and twin tube 2. 9 µL of samples 1, 9 µL of raw lysate, and 2 µL of samples 2 were applied to the gel. Staining: Coomassie Blue-G-250. (**C**,**D**) Purified wildtype TVCV (**C**) and TVCV_PA_ (**D**). Particles (0.1 mg/mL) were negatively stained with 1% uranyl acetate on glow-discharged carbon-coated copper grids. Grids were examined with a TEM (Tecnai G2 Spirit BioTwin, Thermo Fisher Scientific FEI Deutschland GmbH, Dreieich, Germany) operated at 120 kV and equipped with a TemCam XF-416 (TVIPS GmbH, Gilching, Germany).

To ascertain appropriate iodixanol concentrations for the system’s phases, as schematically depicted in Figure 3A (middle), it was imperative to first determine the buoyant densities of TVCV and TVCV_PA_. The buoyant density of TMV in iodixanol was also determined. Isopycnic centrifugation (Figure S1) revealed a buoyant density of 1.205 g/mL (equivalent to 38% (*w*/*v*) iodixanol) for both TVCV and TVCV_PA_. Notably, the significant structural differences did not impact the buoyant densities of these viruses discernibly. TMV exhibited a buoyant density of 1.215 g/mL (equivalent to 40% (*w*/*v*) iodixanol) in the iodixanol gradient, considerably differing from the reported value of 1.32 g/mL in isopycnic CsCl gradients [16,279].

In accordance with these determined buoyant densities, the cushion designed to retain TVCV_(PA)_ was established at a concentration of 42% (*w*/*v*) iodixanol. The retention phase was initially set to 34% (*w*/*v*) iodixanol in the first runs, as estimated from our former experiences. However, under these conditions, the white opaque TVCV_(PA)_ band formed in the retention phase had migrated only a few millimeters down, with low separation from a greenish band held back on top of the retention phase after centrifugation (13.5 h, 25,000 rpm [106,464× *g*]), making it challenging to aspirate the virus band without contamination. Thus, the iodixanol concentration in the retention phase was reduced to 32% (*w*/*v*), which resulted in a well-defined, distinct, white, opaque band on top of the cushion (Figure 3A). SDS-PAGE analysis confirmed that this band was rich in TVCV_PA_ CP and low in proteinaceous contaminants (Figure 3B, sample 2 of twin tubes 1 and 2). In contrast, the greenish band (Figure 3A, arrow 1) contained a large amount of contaminants and only a small amount of CP. Although contaminants could penetrate deeper into the retention phase containing 32% (*w*/*v*) iodixanol, the ample space between the white virus-containing and the greenish, contaminant-rich band facilitated convenient sample collection. Further adjustments, such as increasing the volume of the retention phase and fine-tuning centrifugation speed and time, might potentially improve separation and facilitate further yielding of the virus. For researchers working with viruses or VLPs exhibiting distinct characteristics, this method may be appropriately adapted by exploring the buoyant density of their viral particle of interest, and adjusting the density barrier phases accordingly. Additional purification, if necessary, could probably be accomplished by subjecting the iodixanol-containing virus preparation directly to subsequent isopycnic isolation again in iodixanol, without any intermediate sample treatment.

#### 4.3.2. Preparation Quality and Yield

Our density barrier phase systems of the aforementioned composition yielded 1–2 mg of high-purity TVCV_PA_, or 1–4 g TVCV kg^−1^ symptomatic *N. benthamiana* leaves. This range aligns with the observations for TVCV_PA_ isolated via stepwise enrichment through butanol extraction, two-fold PEG precipitation, and ultracentrifugation. Virion purity, as indicated by SDS-PAGE analysis (Figure 3B), and particle integrity, as observed in TEM images (Figure 3C,D), were both high. The virion length distribution was comparable to that of viruses isolated via PEG solubility gradients [3], with 36% of the virions above 270 nm in length (corresponding to the most abundant length range; Figure S2). Importantly, the storage stability of TVCV_PA_ in iodixanol-containing solutions aspirated from the ultracentrifugation tubes was substantially superior to that of TVCV_PA_ obtained via previously described methods. Those virions resuspended in phosphate buffers experienced a significant decrease in the TVCV_PA_-CP size within several days, which was not preventable by protease inhibitors ([181] and our own experience). Upon storage in iodixanol-supplemented media, however, we did not observe such a size shift in SDS-PAGE after more than five months of storage at 4 °C without any preservative additives.

Iodixanol thus has emerged in this work as a convincing candidate for tuning densities of purification phases in gradients, which also eliminated the necessity for a removal in the downstream applications tested. For example, TVCV_PA_ diluted from the resulting stock solutions of approximately 32% (*w*/*v*) iodixanol was successfully immobilized in 96-well plates directly and retained its IgG-binding capabilities, as activity measurements of HRP immobilized on TVCV_PA_ via IgGs demonstrated (Figure S3). Immobilization at high surface densities on polyelectrolyte-(polyallylamine hydrochloride, PAH)-coated Ta_2_PO_5_ chips for potential biosensor applications was possible, similar to previous observations for TVCV_PA_ isolated via PEG sucrose solubility gradients [3]. The residual iodixanol did not affect TEM image quality either (Figure 3C,D). A minor constraint is the high UV-absorbance of iodixanol, which precludes direct spectrometric determination of TVCV_(PA)_-concentrations, so indirect methods via protein quantification, or comparative band intensity analyses on SDS-PA gels are needed.

### 4.4. Conclusion and Perspectives

As engineered tobamoviruses gain prominence in nanobiotechnology, optimized isolation protocols are becoming increasingly crucial. Our method offers a customizable and scalable approach to isolating TVCV_(PA)_ and probably other viruses with a fair yield and good quality, ensuring compatibility with downstream applications. This method provides an alternative route to establish optimized protocols for challenging virus mutants, paving the way for advancements in nanobiotechnology and biotechnological applications [197].

## 5. An Attempt at a Concluding Outlook

This review has highlighted significant advancements in tobamovirus purification, demonstrating a wide array of techniques and their combinations, among which PEG-mediated virion precipitation is notable for its cost-effectiveness and ability to yield highly pure virus preparations—a method in use for over 60 years [244]. Despite the vast potential applications of tobamoviruses in fields ranging from medicine to energy storage, the prospects for their commercialization remain elusive and will depend on both regulatory issues and the industrial maturity of individual approaches. Many patents have been granted in the area of tobamovirus-assisted technologies, clinical trials are promising, and the urgent worldwide need for ‘green’, sustainable processes and novel biodegradable materials has increased the efforts for repurposing plant viruses as tools and biogenic building blocks further, with budding prospects [130,330,331,332]. For biomedical applications, the development of glycosylation-engineered ‘humanized’ *N. benthamiana* lines is an important additional milestone achieved in recent years [333].

All novel uses and market success of plant-harvested viruses will be affected by their specific purification history, which determines particle quality and availability, underscoring the importance of this comprehensive review of methods. For industrial-scale production, scalability becomes paramount. Historical approaches like continuous ultracentrifugation, capable of producing up to 15 g of four-fold precipitated virus from plant juice within 10 h [89], are now seen as difficult to scale and maintenance-intensive. Potential alternative, extensively scalable methods might include solvent extraction with countercurrent flow for clarification, straightforward large-scale PEG precipitation using tangential flow filtration, and subsequent purification by membrane or monolithic column chromatography (see Section 3.2). Preservation and formulation techniques such as spray drying or spray-freeze-drying may also be employed. Particularly for biomedical approaches, the high quality of the product generated with cleanable and sterilizable instruments is crucial for the selection of a purification method and can only be achieved with constant-quality starting materials and controllable process parameters. In the case of pharmaceuticals production, Good Manufacturing Practice (GMP) and guidelines of regulatory authorities such as the US Food and Drug Administration (FDA) or the European Medicines Agency (EMA) must be adhered to. If standards for tobamovirus purification will be established, lessons learned and methods developed for commercial viral vector production can serve as a guide and may be adapted [334,335]. The preparation of viral vectors typically includes well-defined and documented processes for cell lysis, harvest and clarification, chromatography, ultrafiltration/diafiltration, and sterile filtration, with the removal of empty viral particles being particularly challenging. An elimination of virus-like particles without RNA, or such with packaged heterologous plant RNA, from ‘standardized’ tobamovirus preparations would be similarly demanding if requested by legal authorities in analogy to regulations for mammalian viruses. In turn, plant-infectious virus formulations need to be disarmed due to plant protection regulations, e.g., by appropriate inactivation treatments or by in planta expression or in vitro reconstitution of non-pathogenic particles. The marketing of genetically modified, RNA-containing virions for, e.g., biomedical uses is not compatible, at least with European law, at present anyway. In conclusion, plant virus-specific advantages and limitations have to be taken into account thoroughly upon filing commercial applications in order to counteract prolonged or unsuccessful admission procedures. This will become a major challenge, despite the many convincing properties of tobamoviral derivatives in practically relevant application areas. In any case, biopharmaceutical purification methods that are automated, continuous, utilize single-use equipment, and employ process analytical tools are preferred for approvable commercial components and thus will be of primary importance to pave routes towards the implementation of tobamovirus-based particles in fabrication and formulation processes [336].

Taken together, the methods described in this review can provide solid foundations for the development of large-scale purification processes once commercially viable products are identified. Beyond technical feasibility and product quality, cost-effectiveness remains crucial. An initial calculation suggests surprisingly low production costs for conventional TMV purification at the pilot scale, sufficient for various potential real-world applications of functionalized virions, e.g., in biosensors or for medical drug delivery [118]. Re-visiting the continuously expanded field of options for customized large-scale processes might thus lay the groundwork for economically competitive uses of tobamoviral particles.

## Data Availability

All relevant data are available in the manuscript.

## Supplementary Materials

The following supporting information can be downloaded at: https://www.mdpi.com/article/10.3390/v16060884/s1, Supplementary Table S1. Historical overview of the first purification methods described for virions in different tobamovirus species; references within Table S1. Supplementary Materials S2 presenting additional original data and methods to Section 4.3: The Proven and the New—A Case Study. These include Figure S1: Determination of the buoyant densities of TMV, TVCV, and TVCV_PA_ by isopycnic centrifugation, Figure S2: TEM images of TVCV_PA_ with length class-specific color labels, Figure S3: Assay of TVCV_PA_ functionality after isolation via an iodixanol density barrier phase system and immobilization directly from the iodixanol-containing medium, Figure S4: Illustration of the procedure for setting up three-phased density barrier systems of 5%/32%/42% iodixanol in an ultracentrifugation tube, Table S2: Preparation of stock solutions for a 5%/32%/42% (*w*/*v*) iodixanol density barrier phase system.

## References

[1] Clark F. (1970). Polyethylene glycol solubility gradients, a new and rapid technique for investigations of plant viruses. Virology.

[2] Clark M.F., Lister R.M. (1971). The application of polyethylene glycol solubility-concentration gradients in plant virus research. Virology.

[3] Wendlandt T., Koch C., Britz B., Liedek A., Schmidt N., Werner S., Gleba Y., Vahidpour F., Welden M., Poghossian A. (2023). Facile purification and use of tobamoviral nanocarriers for antibody-mediated display of a two-enzyme system. Viruses.

[4] Altintoprak K., Seidenstücker A., Krolla-Sidenstein P., Plettl A., Jeske H., Gliemann H., Wege C. (2017). RNA-stabilized protein nanorings: High-precision adapters for biohybrid design. Bioinspired Biomim. Nanobiomater..

[5] Schenk A., Eiben S., Goll M., Reith L., Kulak A.N., Meldrum F., Jeske H., Wege C., Ludwigs S. (2017). Virus-directed formation of electrocatalytically active nanoparticle-based Co_3_O_4_ tubes. Nanoscale.

[6] Schneider A., Eber F.J., Wenz N.L., Altintoprak K., Jeske H., Eiben S., Wege C. (2016). Dynamic DNA-controlled “stop-and-go” assembly of well-defined protein domains on RNA-scaffolded TMV-like nanotubes. Nanoscale.

[7] Wenz N., Piasecka S., Kalinowski M., Schneider A., Richert C., Wege C. (2018). Building expanded structures from tetrahedral DNA branching elements, RNA and TMV protein. Nanoscale.

[8] Eber F.J., Eiben S., Jeske H., Wege C. (2015). RNA-controlled assembly of tobacco mosaic virus-derived complex structures: From nanoboomerangs to tetrapods. Nanoscale.

[9] Eber F.J., Eiben S., Jeske H., Wege C. (2013). Bottom-up-assembled nanostar colloids of gold cores and tubes derived from tobacco mosaic virus. Angew. Chem. Int. Ed..

[10] Eiben S., Stitz N., Eber F., Wagner J., Atanasova P., Bill J., Wege C., Jeske H. (2014). Tailoring the surface properties of tobacco mosaic virions by the integration of bacterially expressed mutant coat protein. Virus Res..

[11] Koch C., Eber F.J., Azucena C., Foerste A., Walheim S., Schimmel T., Bittner A., Jeske H., Gliemann H., Eiben S. (2016). Novel roles for well-known players: From tobacco mosaic virus pests to enzymatically active assemblies. Beilstein J. Nanotechnol..

[12] Altintoprak K., Seidenstücker A., Welle A., Eiben S., Atanasova P., Stitz N., Plettl A., Bill J., Gliemann H., Jeske H. (2015). Peptide-equipped tobacco mosaic virus templates for selective and controllable biomineral deposition. Beilstein J. Nanotechnol..

[13] Geiger F.C., Eber F.J., Eiben S., Mueller A., Jeske H., Spatz J.P., Wege C. (2013). TMV nanorods with programmed longitudinal domains of differently addressable coat proteins. Nanoscale.

[14] Atanasova P., Rothenstein D., Schneider J.J., Hoffmann R.C., Dilfer S., Eiben S., Wege C., Jeske H., Bill J. (2011). Virus-templated synthesis of ZnO nanostructures and formation of field-effect transistors. Adv. Mater..

[15] Mueller A., Eber F.J., Azucena C., Petershans A., Bittner A.M., Gliemann H., Jeske H., Wege C. (2011). Inducible site-selective bottom-up assembly of virus-derived nanotube arrays on RNA-equipped wafers. ACS Nano.

[16] Kadri A., Maiss E., Amsharov N., Bittner A.M., Balci S., Kern K., Jeske H., Wege C. (2011). Engineered Tobacco mosaic virus mutants with distinct physical characteristics in planta and enhanced metallization properties. Virus Res..

[17] Mueller A., Kadri A., Jeske H., Wege C. (2010). In vitro assembly of Tobacco mosaic virus coat protein variants derived from fission yeast expression clones or plants. J. Virol. Methods.

[18] Balci S., Bittner A.M., Schirra M., Thonke K., Sauer R., Hahn K., Kadri A., Wege C., Jeske H., Kern K. (2009). Catalytic coating of virus particles with zinc oxide. Electrochim. Acta.

[19] Balci S., Leinberger D.M., Knez M., Bittner A.M., Boes F., Kadri A., Wege C., Jeske H., Kern K. (2008). Printing and aligning mesoscale patterns of tobacco mosaic viruses on surfaces. Adv. Mater..

[20] Balci S., Noda K., Bittner A.M., Kadri A., Wege C., Jeske H., Kern K. (2007). Self-assembly of metal-virus nanodumbbells. Angew. Chem. Int. Ed..

[21] Knez M., Bittner A.M., Boes F., Wege C., Jeske H., Maiss E., Kern K. (2003). Biotemplate synthesis of 3-nm nickel and cobalt nanowires. Nano Lett..

[22] Knez M., Sumser M., Bittner A.M., Wege C., Jeske H., Kooi S., Burghard M., Kern K. (2002). Electrochemical modification of individual nano-objects. J. Electroanal. Chem..

[23] Steere R.L., Smith K.M., Lauffer M.A. (1959). The purification of plant viruses. Advances in Virus Research.

[24] Stanley W.M. (1937). Chemical studies on the virus of tobacco mosaic: VIII. The isolation of a crystalline protein possessing the properties of aucuba mosaic virus. J. Biol. Chem..

[25] Kreibig U., Wetter C. (1980). Light diffraction of in vitro crystals of six tobacco mosaic viruses. Z. Naturforsch. Sect. C.

[26] Bos L. (1999). Beijerinck’s work on tobacco mosaic virus: Historical context and legacy. Philos. Trans. R. Soc. London Ser. B Biol. Sci..

[27] Denyer S.P., Hodges N.A. (2008). 12.4 Filtration sterilization. Russell, Hugo & Ayliffe’s Principles and Practice of Disinfection, Preservation and Sterilization.

[28] Beijerinck M.W. (1898). Über ein contagium vivum fluidum als Ursache der Fleckenkrankheit der Tabaksblätter. Verhandel. Koninkl. Akad. Wettenschappen Amst..

[29] Ivanowski D. (1892). Ueber die Mosaikkrankheit der Tabakpflanze/Concerning the mosaic disease of the tobacco plant (translation). Bull. L’académie Impériale Sci. Saint-Pétersbourg/Transl. Phytopathol. Class.

[30] Lecoq H. (2001). Découverte du premier virus, le virus de la mosaïque du tabac: 1892 ou 1898?. Comptes Rendus L’académie Sci.-Ser. III-Sci. Vie.

[31] Lustig A., Levine A.J. (1992). One hundred years of virology. J. Virol..

[32] Scholthof K.B. (2004). Tobacco mosaic virus: A model system for plant biology. Annu. Rev. Phytopathol..

[33] Creager A.N.H. (2022). Tobacco mosaic virus and the history of molecular biology. Annu. Rev. Virol..

[34] Scholthof K.-B.G., Shaw J.G., Zaitlin M. (1999). Tobacco Mosaic Virus: One Hundred Years of Contributions to Virology.

[35] Bos L. (2000). 100 years of virology: From vitalism via molecular biology to genetic engineering. Trends Microbiol..

[36] van der Want J.P.H., Dijkstra J. (2006). A history of plant virology. Arch. Virol..

[37] Zerbini F.M., Kitajima E.W. (2022). From Contagium vivum fluidum to Riboviria: A tobacco mosaic virus-centric history of virus taxonomy. Biomolecules.

[38] Ivanowski D. (1903). Ueber die Mosaikkrankheit der Tabakpflanze. Z. Pflanzenkrankh..

[39] Baur E. (1906). Über die infektiöse Chlorose der Malvaceen. Sitzber. Kgl. Preuss. Akad. Wiss..

[40] Wege C., Gotthardt R.D., Frischmuth T., Jeske H. (2000). Fulfilling Koch’s postulates for Abutilon mosaic virus. Arch. Virol..

[41] Smith E.F. (1894). Ueber die Mosaikkrankheit des Tabaks by Adolf Mayer. J. Mycol..

[42] Koning C.J. (1899). Die Flecken- oder Mosaikkrankheit des holländischen Tabaks. Z. Pflanzenkrankh..

[43] Bechhold H. (1907). Die Gallertfiltration (Ultrafiltration.). Z. Chem. Ind. Kolloide.

[44] Holmes F.O. (1929). Local lesions in tobacco mosaic. Bot. Gaz..

[45] Purdy H.A. (1929). Immunologic reactions with tobacco mosaic virus. J. Exp. Med..

[46] Zaitlin M., Palukaitis P. (2000). Advances in understanding plant viruses and virus diseases. Annu. Rev. Phytopathol..

[47] Vinson C. (1934). Possible chemical nature of tobacco mosaic virus. Science.

[48] Stanley W.M., Loring H.S. (1936). The isolation of crystalline tobacco mosaic virus protein from diseased tomato plants. Science.

[49] Stanley W.M. (1935). Isolation of a crystalline protein possessing the properties of tobacco-mosaic virus. Science.

[50] Stanley W. (1938). Isolation and properties of tobacco mosaic and other virus proteins: Harvey lecture, March 17, 1938. Bull. N. Y. Acad. Med..

[51] Svedberg T., Rinde H. (1924). The ultra-centrifuge, a new instrument for the determination of size and distribution of size of particle in amicroscopic colloids. J. Am. Chem. Soc..

[52] Biscoe J., Pickels E.G., Wyckoff R.W. (1936). An air-driven ultracentrifuge for molecular sedimentation. J. Exp. Med..

[53] Eriksson-Quensel I.-B., Svedberg T. (1936). Sedimentation and electrophoresis of the tobacco mosaic virus protein. J. Am. Chem. Soc..

[54] Wyckoff R.W.G. (1937). The ultracentrifugal study of virus proteins. Proc. Am. Philos. Soc..

[55] Bawden F.C., Pirie N.W., Bernal J.D., Fankuchen I. (1936). Liquid crystalline substances from virus infected plants. Nature.

[56] Bawden F.C., Pirie N.W. (1937). The isolation and some properties of liquid crystalline substances from solanaceous plants infected with three strains of tobacco mosaic virus. Proc. R. Soc. London Ser. B Biol. Sci..

[57] Pennazio S., Roggero P. (2000). The discovery of the chemical nature of tobacco mosaic virus. Riv. Biol..

[58] Bernal J.D., Fankuchen I. (1937). Structure types of protein ‘crystals’ from virus infected plants. Nature.

[59] Kausche G., Guggisberg H., Wissler A. (1939). Quantitative Untersuchung der Strömungsdoppelbrechung von Tabakmosaik-und Kartoffel-X-Virus. Naturwissenschaften.

[60] Lauffer M.A. (1939). The electro-optical effect in certain viruses. J. Am. Chem. Soc..

[61] Robinson J. (1939). Shape of tobacco mosaic virus particles in solution. Nature.

[62] Robinson J. (1939). Studies in the viscosity of colloids. I. The anomalous viscosity of dilute suspensions of rigid anisometric particles. Proc. R. Soc. Lond. Ser. A Math. Phys. Sci..

[63] Bernal J.D., Fankuchen I. (1941). X-Ray and crystallographic studies of plant virus preparations: I. Introduction and preparation of specimens ii. Modes of aggregation of the virus particles. J. Gen. Physiol..

[64] Kausche G. (1938). Über die Charakterisierung von pflanzlichen Virussolen mit kolloidem Gold. Naturwissenschaften.

[65] Adams M., Fraden S. (1998). Phase behavior of mixtures of rods (tobacco mosaic virus) and spheres (polyethylene oxide, bovine serum albumin). Biophys. J..

[66] Liljeström V., Ora A., Hassinen J., Rekola H.T., Nonappa, Heilala M., Hynninen V., Joensuu J.J., Ras R.H.A., Törmä P. (2017). Cooperative colloidal self-assembly of metal-protein superlattice wires. Nat. Commun..

[67] Wu Z., Mueller A., Degenhard S., Ruff S.E., Geiger F., Bittner A., Wege C., Krill C. (2010). Enhancing the magnetoviscosity of ferrofluids by the addition of biological nanotubes. ACS Nano.

[68] Khan A.A., Fox E.K., Gorzny M.L., Nikulina E., Brougham D.F., Wege C., Bittner A.M. (2013). pH Control of the Electrostatic Binding of Gold and Iron Oxide Nanoparticles to Tobacco Mosaic Virus. Langmuir.

[69] Bawden F.C., Pirie N.W. (1943). Methods for the purification of tomato bushy stunt and tobacco mosaic viruses. Biochem. J..

[70] Pfankuch E., Hagenguth K. (1943). Isolierung einiger pflanzlicher Viren. Naturwissenschaften.

[71] Lauffer M.A. (1944). The size and shape of tobacco mosaic virus particles1. J. Am. Chem. Soc..

[72] Gelderblom H.R., Krüger D.H., Hawkes P.W. (2014). Helmut Ruska (1908–1973): His role in the evolution of electron microscopy in the life sciences, and especially virology. Advances in Imaging and Electron Physics.

[73] Kausche G.A., Pfankuch E., Ruska H. (1939). Die Sichtbarmachung von pflanzlichem Virus im Übermikroskop. Naturwissenschaften.

[74] Creager A.N. (2002). The Life of a Virus: Tobacco Mosaic Virus as an Experimental Model, 1930–1965.

[75] Creager A.N., Morgan G.J. (2008). After the double helix: Rosalind Franklin’s research on Tobacco mosaic virus. Isis.

[76] Franklin R.E., Klug A., Holmes K.C. (1957). X-ray diffraction studies of the structure and morphology of tobacco mosaic virus. Proceedings of the Ciba Foundation Symposium on the Nature of Viruses.

[77] Klein J. (1983). Max-Planck-Institut für Biologie Tübingen: Die Geschichte des Instituts von 1912 bis 1983.

[78] Schramm G. (1948). Zur Chemie des Mutationsvorganges beim Tabakmosaik-Virus. Z. Naturforschung B.

[79] Lewis J. (2004). From virus research to molecular biology: Tobacco mosaic virus in Germany, 1936–1956. J. Hist. Biol..

[80] Gierer A., Schramm G. (1956). Infectivity of ribonucleic acid from tobacco mosaic virus. Nature.

[81] Gierer A., Schramm G. (1956). Die Infektiosität der Nucleinsäure aus Tabakmosaikvirus. Z. Naturforschung B.

[82] Schramm G., Schumacher G., Zillig W. (1955). Über die Struktur des Tabakmosaikvirus: III. Mitt.: Der Zerfall in alkalischer Lösung. Z. Naturforsch. Sect. B.

[83] Fraenkel-Conrat H. (1956). The role of the nucleic acid in the reconstitution of active tobacco mosaic virus. J. Am. Chem. Soc..

[84] Fraenkel-Conrat H., Singer B., Williams R. (1957). Infectivity of viral nucleic acid. Biochim. Biophys. Acta.

[85] Watanabe I., Kawade Y. (1953). Purification and characterization of tobacco mosaic virus. Bull. Chem. Soc. Jpn..

[86] Fukushi T., Shikata E. (1955). Electron microscope studies on plant viruses I. J. Fac. Agric. Hokkaido Univ..

[87] Ryjkoff V., Smirnova M.V. (1941). Liquid crystals of the virus of the tobacco mosaic (Nicotiana virus 1 Allard). Dokl. Akad. Nauk. SSSR.

[88] Ryzkov V., Tarasevic L., Loïdina G. (1950). The effect of strong solutions of sodium nucleinate on the virus of the mosaic disease of tobacco (VTM) and on albumen. Dokl. Akad. Nauk. SSSR.

[89] Stanley W.M. (1942). The concentration and purification of tobacco mosaic virus by means of the sharples super-centrifuge. J. Am. Chem. Soc..

[90] Carr J.P., Talbot N.J. (2004). Tobacco mosaic virus. Plant-Pathogen Interactions.

[91] Hamilton R.I., Edwardson J.R., Francki R.I.B., Hsu H.T., Hull R., Koenig R., Milne R.G. (1981). Guidelines for the identification and characterization of plant viruses. J. Gen. Virol..

[92] Van Regenmortel M.H.V. (1975). Antigenic relationships between strains of tobacco mosaic virus. Virology.

[93] Van Regenmortel M.H.V., Van Regenmortel M.H.V., Fraenkel-Conrat H. (1986). Tobacco mosaic virus antigenic structure. The Plant Viruses: The Rod-Shaped Plant Viruses.

[94] Smith K.M. (1931). On the composite nature of certain potato virus diseases of the mosaic group as revealed by the use of plant indicators and selective methods of transmission. Proc. R. Soc. Lond. Ser. B Contain. Pap. A Biol. Character.

[95] Mehetre G.T., Leo V.V., Singh G., Sorokan A., Maksimov I., Yadav M.K., Upadhyaya K., Hashem A., Alsaleh A.N., Dawoud T.M. (2021). Current developments and challenges in plant viral diagnostics: A systematic review. Viruses.

[96] Bernabé-Orts J.M., Torre C., Méndez-López E., Hernando Y., Aranda M.A. (2021). New resources for the specific and sensitive detection of the emerging tomato brown rugose fruit virus. Viruses.

[97] Mandal S., Mandal B., Haq Q., Varma A. (2008). Properties, diagnosis and management of Cucumber green mottle mosaic virus. Plant Viruses.

[98] Wilkins M., Stokes A., Seeds W., Oster G. (1950). Tobacco mosaic virus crystals and three-dimensional microscopic vision. Nature.

[99] Klug A. (1999). The tobacco mosaic virus particle: Structure and assembly. Philos. Trans. R. Soc. Lond. Ser. B Biol. Sci..

[100] Boedtker H., Simmons N.S. (1958). The preparation and characterization of essentially uniform tobacco mosaic virus particles. J. Am. Chem. Soc..

[101] Kendall A., Stubbs G. (2016). Fibre diffraction in the analysis of filamentous virus structure. Crystallogr. Rev..

[102] Gebhardt R., Teulon J.-M., Pellequer J.-L., Burghammer M., Colletier J.-P., Riekel C. (2014). Virus particle assembly into crystalline domains enabled by the coffee ring effect. Soft Matter.

[103] Wang H., Planchart A., Allen D., Pattanayek R., Stubbs G. (1993). Preliminary X-ray diffraction studies of ribgrass mosaic virus. J. Mol. Biol..

[104] Fromm S.A., Bharat T.A., Jakobi A.J., Hagen W.J., Sachse C. (2015). Seeing tobacco mosaic virus through direct electron detectors. J. Struct. Biol..

[105] Song B., Lenhart J., Flegler V.J., Makbul C., Rasmussen T., Böttcher B. (2019). Capabilities of the Falcon III detector for single-particle structure determination. Ultramicroscopy.

[106] Lewis J.K., Bendahmane M., Smith T.J., Beachy R.N., Siuzdak G. (1998). Identification of viral mutants by mass spectrometry. Proc. Natl. Acad. Sci. USA.

[107] Lumata J.L., Ball D., Shahrivarkevishahi A., Luzuriaga M.A., Herbert F.C., Brohlin O., Lee H., Hagge L.M., D’Arcy S., Gassensmith J.J. (2021). Identification and physical characterization of a spontaneous mutation of the tobacco mosaic virus in the laboratory environment. Sci. Rep..

[108] Fraile A., Escriu F., Aranda M.A., Malpica J.M., Gibbs A.J., García-Arenal F. (1997). A century of tobamovirus evolution in an Australian population of *Nicotiana glauca*. J. Virol..

[109] Gibbs A. (1999). Evolution and origins of tobamoviruses. Philos. Trans. R. Soc. Lond. Ser. B Biol. Sci..

[110] Gibbs A.J., Wood J., Garcia-Arenal F., Ohshima K., Armstrong J.S. (2015). Tobamoviruses have probably co-diverged with their eudicotyledonous hosts for at least 110 million years. Virus Evol..

[111] Wilson T.M.A. (1989). Plant viruses: A tool-box for genetic engineering and crop protection. Bioessays.

[112] Haynes J.R., Cunningham J., von Seefried A., Lennick M., Garvin R.T., Shen S.-H. (1986). Development of a genetically–engineered, candidate polio vaccine employing the self–assembling properties of the tobacco mosaic virus coat protein. Biotechnology.

[113] Lomonossoff G.P., Wege C., Palukaitis P., Roossinck M.J. (2018). Chapter Six-TMV particles: The journey from fundamental studies to bionanotechnology applications. Advances in Virus Research.

[114] Wen A.M., Lee K.L., Steinmetz N.F., Norris P.M., Friedersdorf L.E. (2020). Plant Virus-Based Nanotechnologies. Women in Nanotechnology: Contributions from the Atomic Level and Up.

[115] Zhang J., He H., Zeng F., Du M., Huang D., Chen G., Wang Q. (2024). Advances of structural design and biomedical applications of tobacco mosaic virus coat protein. Adv. Nanobiomed Res..

[116] Fan X.Z., Pomerantseva E., Gnerlich M., Brown A., Gerasopoulos K., McCarthy M., Culver J., Ghodssi R. (2013). Tobacco mosaic virus: A biological building block for micro/nano/biosystems. J. Vac. Sci. Technol. A.

[117] Culver J.N., Brown A.D., Zang F., Gnerlich M., Gerasopoulos K., Ghodssi R. (2015). Plant virus directed fabrication of nanoscale materials and devices. Virology.

[118] Wege C., Koch C. (2020). From stars to stripes: RNA-directed shaping of plant viral protein templates—Structural synthetic virology for smart biohybrid nanostructures. WIREs Nanomed. Nanobiotechnol..

[119] Dragnea B. (2017). Virus-based devices: Prospects for allopoiesis. ACS Nano.

[120] Steele J.F.C., Peyret H., Saunders K., Castells-Graells R., Marsian J., Meshcheriakova Y., Lomonossoff G.P. (2017). Synthetic plant virology for nanobiotechnology and nanomedicine. Wiley Interdiscip. Rev. Nanomed. Nanobiotechnol..

[121] Lin B., Ratna B. (2014). Virus Hybrids as Nanomaterials: Methods and Protocols.

[122] Nkanga C.I., Steinmetz N.F. (2021). The pharmacology of plant virus nanoparticles. Virology.

[123] Czapar A.E., Steinmetz N.F. (2017). Plant viruses and bacteriophages for drug delivery in medicine and biotechnology. Curr. Opin. Chem. Biol..

[124] Khudyakov Y., Pumpens P. (2016). Viral Nanotechnology.

[125] Arul S.S., Balakrishnan B., Handanahal S.S., Venkataraman S. (2024). Viral nanoparticles: Current advances in design and development. Biochimie.

[126] Eiben S., Koch C., Altintoprak K., Southan A., Tovar G., Laschat S., Weiss I., Wege C. (2019). Plant virus-based materials for biomedical applications: Trends and prospects. Adv. Drug Rev..

[127] Chariou P.L., Ortega-Rivera O.A., Steinmetz N.F. (2020). Nanocarriers for the Delivery of Medical, Veterinary, and Agricultural Active Ingredients. ACS Nano.

[128] Takeyama N., Kiyono H., Yuki Y. (2015). Plant-based vaccines for animals and humans: Recent advances in technology and clinical trials. Ther. Adv. Vaccines.

[129] Kolotilin I., Topp E., Cox E., Devriendt B., Conrad U., Joensuu J., Stoger E., Warzecha H., McAllister T., Potter A. (2014). Plant-based solutions for veterinary immunotherapeutics and prophylactics. Vet. Res..

[130] Chung Y.H., Church D., Koellhoffer E.C., Osota E., Shukla S., Rybicki E.P., Pokorski J.K., Steinmetz N.F. (2021). Integrating plant molecular farming and materials research for next-generation vaccines. Nat. Rev. Mater..

[131] Crisci E., Bárcena J., Montoya M. (2012). Virus-like particles: The new frontier of vaccines for animal viral infections. Vet. Immunol. Immunopathol..

[132] Nikitin N., Vasiliev Y., Kovalenko A., Ryabchevskaya E., Kondakova O., Evtushenko E., Karpova O. (2023). Plant Viruses as Adjuvants for Next-Generation Vaccines and Immunotherapy. Vaccines.

[133] Williams L., Jurado S., Llorente F., Romualdo A., González S., Saconne A., Bronchalo I., Martínez-Cortes M., Pérez-Gómez B., Ponz F. (2021). The C-Terminal Half of SARS-CoV-2 Nucleocapsid Protein, Industrially Produced in Plants, Is Valid as Antigen in COVID-19 Serological Tests. Front. Plant Sci..

[134] Azizi M., Shahgolzari M., Fathi-Karkan S., Ghasemi M., Samadian H. (2023). Multifunctional plant virus nanoparticles: An emerging strategy for therapy of cancer. WIREs Nanomed. Nanobiotechnol..

[135] Venkataraman S., Hefferon K. (2021). Application of plant viruses in biotechnology, medicine, and human health. Viruses.

[136] Chariou P.L., Ma Y., Hensley M., Rosskopf E.N., Hong J.C., Charudattan R., Steinmetz N.F. (2021). Inactivated plant viruses as an agrochemical delivery platform. ACS Agric. Sci. Technol..

[137] Charudattan R. (2024). Use of plant viruses as bioherbicides: The first virus-based bioherbicide and future opportunities. Pest. Manag. Sci..

[138] Schlick T.L., Ding Z.B., Kovacs E.W., Francis M.B. (2005). Dual-surface modification of the tobacco mosaic virus. J. Am. Chem. Soc..

[139] González-Gamboa I., Caparco A.A., McCaskill J., Fuenlabrada-Velázquez P., Hays S.S., Jin Z., Jokerst J.V., Pokorski J.K., Steinmetz N.F. (2024). Inter-coat protein loading of active ingredients into Tobacco mild green mosaic virus through partial dissociation and reassembly of the virion. Sci. Rep..

[140] Gulati N.M., Pitek A.S., Steinmetz N.F., Stewart P.L. (2017). Cryo-electron tomography investigation of serum albumin-camouflaged tobacco mosaic virus nanoparticles. Nanoscale.

[141] Dickmeis C., Kauth L., Commandeur U. (2021). From infection to healing: The use of plant viruses in bioactive hydrogels. WIREs Nanomed. Nanobiotechnol..

[142] Maturavongsadit P., Luckanagul J.A., Metavarayuth K., Zhao X., Chen L.M., Lin Y., Wang Q. (2016). Promotion of in vitro chondrogenesis of mesenchymal stem cells using in situ hyaluronic hydrogel functionalized with rod-like viral nanoparticles. Biomacromolecules.

[143] Schuphan J., Stojanović N., Lin Y.-Y., Buhl E.M., Aveic S., Commandeur U., Schillberg S., Fischer H. (2024). A combination of flexible modified plant virus nanoparticles enables additive effects resulting in improved osteogenesis. Adv. Healthc. Mater..

[144] Alonso J.M., Górzny M.Ł., Bittner A.M. (2013). The physics of tobacco mosaic virus and virus-based devices in biotechnology. Trends Biotechnol..

[145] Riekel C., Burghammer M., Snigirev I., Rosenthal M. (2018). Microstructural metrology of tobacco mosaic virus nanorods during radial compression and heating. Soft Matter.

[146] Wu L.Y., Zang J.F., Lee L.A., Niu Z.W., Horvatha G.C., Braxtona V., Wibowo A.C., Bruckman M.A., Ghoshroy S., zur Loye H.C. (2011). Electrospinning fabrication, structural and mechanical characterization of rod-like virus-based composite nanofibers. J. Mater. Chem..

[147] Rahman M.M., Ölçeroğlu E., McCarthy M. (2014). Biotemplates: Scalable nanomanufacturing of virus-templated coatings for enhanced boiling. Adv. Mater. Interfaces.

[148] Poghossian A., Jablonski M., Koch C., Bronder T.S., Rolka D., Wege C., Schöning M.J. (2018). Field-effect biosensor using virus particles as scaffolds for enzyme immobilization. Biosens. Bioelectron..

[149] Koch C., Poghossian A., Schoening M.J., Wege C. (2018). Penicillin detection by tobacco mosaic virus-assisted colorimetric biosensors. Nanotheranostics.

[150] Poghossian A., Jablonski M., Molinnus D., Wege C., Schoning M.J. (2020). Field-effect sensors for virus detection: From Ebola to SARS-CoV-2 and plant viral enhancers. Front. Plant Sci..

[151] Douglas T., Young M. (1999). Virus particles as templates for materials synthesis. Adv. Mater..

[152] Shenton W., Douglas T., Young M., Stubbs G., Mann S. (1999). Inorganic-organic nanotube composites from template mineralization of tobacco mosaic virus. Adv. Mater..

[153] Young M., Willits D., Uchida M., Douglas T. (2008). Plant viruses as biotemplates for materials and their use in nanotechnology. Annu. Rev. Phytopathol..

[154] Bittner A.M., Alonso J.M., Górzny M.L., Wege C., Mateu M.G., Harris J.R. (2013). Nanoscale science and technology with plant viruses and bacteriophages. Structure and Physics of Viruses: An Integrated Textbook.

[155] Fan X.Z., Naves L., Siwak N.P., Brown A., Culver J., Ghodssi R. (2015). Integration of genetically modified virus-like-particles with an optical resonator for selective bio-detection. Nanotechnology.

[156] Soto C.M., Ratna B.R. (2010). Virus hybrids as nanomaterials for biotechnology. Curr. Opin. Biotechnol..

[157] Chen Z., Li N., Li S., Dharmarwardana M., Schlimme A., Gassensmith J.J. (2016). Viral chemistry: The chemical functionalization of viral architectures to create new technology. Wiley Interdiscip. Rev. Nanomed. Nanobiotechnol..

[158] Chu S., Brown A.D., Culver J.N., Ghodssi R. (2018). Tobacco mosaic virus as a versatile platform for molecular assembly and device fabrication. Biotechnol. J..

[159] Balci S., Hahn K., Kopold P., Kadri A., Wege C., Kern K., Bittner A.M. (2012). Electroless synthesis of 3 nm wide alloy nanowires inside Tobacco mosaic virus. Nanotechnology.

[160] Bittner A.M., Wege C., Lomonossoff G.P. (2018). TMV-Templated Formation of Metal and Polymer Nanotubes. Virus-Derived Nanoparticles for Advanced Technologies: Methods and Protocols.

[161] Shah S.N., Heddle J.G., Evans D.J., Lomonossoff G.P. (2023). Production of Metallic Alloy Nanowires and Particles Templated Using Tomato Mosaic Virus (ToMV). Nanomaterials.

[162] Miller R.A., Stephanopoulos N., McFarland J.M., Rosko A.S., Geissler P.L., Francis M.B. (2010). Impact of assembly state on the defect tolerance of TMV-based light harvesting arrays. J. Am. Chem. Soc..

[163] Rong J.H., Oberbeck F., Wang X.N., Li X.D., Oxsher J., Niu Z.W., Wang Q. (2009). Tobacco mosaic virus templated synthesis of one dimensional inorganic-polymer hybrid fibres. J. Mater. Chem..

[164] Dönmez Güngüneş Ç., Başçeken S., Elçin A.E., Elçin Y.M. (2022). Fabrication and molecular modeling of navette-shaped fullerene nanorods using tobacco mosaic virus as a nanotemplate. Mol. Biotechnol..

[165] Riccò R., Liang W., Li S., Gassensmith J.J., Caruso F., Doonan C., Falcaro P. (2018). Metal–organic frameworks for cell and virus biology: A perspective. ACS Nano.

[166] Moreira Da Silva C., Ortiz-Peña N., Boubekeur-Lecaque L., Dušek J., Moravec T., Alloyeau D., Ha-Duong N.-T. (2023). In situ insights into the nucleation and growth mechanisms of gold nanoparticles on tobacco mosaic virus. Nano Lett..

[167] Gerasopoulos K., McCarthy M., Royston E., Culver J.N., Ghodssi R. (2008). Nanostructured nickel electrodes using the Tobacco mosaic virus for microbattery applications. J. Micromech. Microeng..

[168] Royston E., Ghosh A., Kofinas P., Harris M.T., Culver J.N. (2008). Self-assembly of virus-structured high surface area nanomaterials and their application as battery electrodes. Langmuir.

[169] Tseng R.J., Tsai C., Ma L.P., Ouyang J., Ozkan C.S., Yang Y. (2006). Digital memory device based on tobacco mosaic virus conjugated with nanoparticles. Nat. Nanotechnol..

[170] Yang C.X., Choi C.H., Lee C.S., Yi H.M. (2013). A facile synthesis-fabrication strategy for integration of catalytically active viral-palladium nanostructures into polymeric hydrogel microparticles via replica molding. ACS Nano.

[171] Chiang C.-Y., Epstein J., Brown A., Munday J.N., Culver J.N., Ehrman S. (2012). Biological templates for antireflective current collectors for photoelectrochemical cell applications. Nano Lett..

[172] Lee K.Z., Basnayake Pussepitiyalage V., Lee Y.-H., Loesch-Fries L.S., Harris M.T., Hemmati S., Solomon K.V. (2021). Engineering tobacco mosaic virus and its virus-like-particles for synthesis of biotemplated nanomaterials. Biotechnol. J..

[173] Zhou Q., Liu X., Tian Y., Wu M., Niu Z. (2017). Mussel-inspired polydopamine coating on tobacco mosaic virus: One-dimensional hybrid nanofibers for gold nanoparticle growth. Langmuir.

[174] Love A.J., Makarov V., Yaminsky I., Kalinina N.O., Taliansky M.E. (2014). The use of tobacco mosaic virus and cowpea mosaic virus for the production of novel metal nanomaterials. Virology.

[175] Hou C., Xu H., Jiang X., Li Y., Deng S., Zang M., Xu J., Liu J. (2021). Virus-based supramolecular structure and materials: Concept and prospects. ACS Appl. Bio Mater..

[176] Love A.J., Makarov V.V., Sinitsyna O.V., Shaw J., Yaminsky I.V., Kalinina N.O., Taliansky M. (2015). A genetically modified tobacco mosaic virus that can produce gold nanoparticles from a metal salt precursor. Front. Plant Sci..

[177] Zakeri B., Fierer J.O., Celik E., Chittock E.C., Schwarz-Linek U., Moy V.T., Howarth M. (2012). Peptide tag forming a rapid covalent bond to a protein, through engineering a bacterial adhesin. Proc. Natl. Acad. Sci. USA.

[178] Schuphan J., Commandeur U. (2021). Analysis of engineered tobacco mosaic virus and potato virus X nanoparticles as carriers for biocatalysts. Front. Plant Sci..

[179] Röder J., Fischer R., Commandeur U. (2017). Adoption of the 2A ribosomal skip principle to tobacco mosaic virus for peptide display. Front. Plant Sci..

[180] Frolova O.Y., Petrunia I.V., Komarova T.V., Kosorukov V.S., Sheval E.V., Gleba Y.Y., Dorokhov Y.L. (2010). Trastuzumab-binding peptide display by Tobacco mosaic virus. Virology.

[181] Werner S., Marillonnet S., Hause G., Klimyuk V., Gleba Y. (2006). Immunoabsorbent nanoparticles based on a tobamovirus displaying protein A. Proc. Natl. Acad. Sci. USA.

[182] Bäcker M., Koch C., Eiben S., Geiger F., Eber F.J., Gliemann H., Poghossian A., Wege C., Schöning M.J. (2017). Tobacco mosaic virus as enzyme nanocarrier for electrochemical biosensors. Sens. Actuators B.

[183] Welden M., Poghossian A., Vahidpour F., Wendlandt T., Keusgen M., Christina W., Schöning M.J. (2023). Capacitive field-effect biosensor modified with a stacked bilayer of weak polyelectrolyte and plant virus particles as enzyme nanocarriers. Bioelectrochemistry.

[184] Welden M., Poghossian A., Vahidpour F., Wendlandt T., Keusgen M., Wege C., Schöning M.J. (2022). Towards multi-analyte detection with field-effect capacitors modified with tobacco mosaic virus bioparticles as enzyme nanocarriers. Biosensors.

[185] Welden M., Severins R., Poghossian A., Wege C., Bongaerts J., Siegert P., Keusgen M., Schoning M.J. (2022). Detection of acetoin and diacetyl by a tobacco mosaic virus-assisted field-effect biosensor. Chemosensors.

[186] Koch C., Wabbel K., Eber F.J., Krolla-Sidenstein P., Azucena C., Gliemann H., Eiben S., Geiger F., Wege C. (2015). Modified TMV particles as beneficial scaffolds to present sensor enzymes. Front. Plant Sci..

[187] Zang F., Gerasopoulos K., Fan X.Z., Brown A.D., Culver J.N., Ghodssi R. (2016). Real-time monitoring of macromolecular biosensing probe self-assembly and on-chip ELISA using impedimetric microsensors. Biosens. Bioelectron..

[188] Zang F., Gerasopoulos K., Brown A.D., Culver J.N., Ghodssi R. (2017). Capillary microfluidics-assembled virus-like particle bionanoreceptor interfaces for label-free biosensing. ACS Appl. Mater. Interfaces.

[189] Grübel J., Wendlandt T., Urban D., Jauch C.O., Wege C., Tovar G.E.M., Southan A. (2023). Soft sub-structured multi-material biosensor hydrogels with enzymes retained by plant viral scaffolds. Macromol. Biosci..

[190] Paiva T.O., Schneider A., Bataille L., Chovin A., Anne A., Michon T., Wege C., Demaille C. (2022). Enzymatic activity of individual bioelectrocatalytic viral nanoparticles: Dependence of catalysis on the viral scaffold and its length. Nanoscale.

[191] Yi H., Rubloff G.W., Culver J.N. (2007). TMV microarrays:  Hybridization-based assembly of DNA-programmed viral nanotemplates. Langmuir.

[192] Bruckman M.A., Steinmetz N.F., Lin B., Ratna B. (2014). Chemical Modification of the Inner and Outer Surfaces of Tobacco Mosaic Virus (TMV). Virus Hybrids as Nanomaterials: Methods and Protocols.

[193] Niu Z., Bruckman M.A., Li S., Lee L.A., Lee B., Pingali S.V., Thiyagarajan P., Wang Q. (2007). Assembly of tobacco mosaic virus into fibrous and macroscopic bundled arrays mediated by surface aniline polymerization. Langmuir.

[194] Mukerrem D., Michael H.B.S. (2002). A chemoselective biomolecular template for assembling diverse nanotubular materials. Nanotechnology.

[195] Dickmeis C., Altintoprak K., van Rijn P., Wege C., Commandeur U., Wege C., Lomonossoff G.P., Walker J.M. (2018). Bioinspired silica mineralization on viral templates. Virus-Derived Nanoparticles for Advanced Technologies.

[196] Sacco A., Barzan G., Matić S., Giovannozzi A.M., Rossi A.M., D’Errico C., Vallino M., Ciuffo M., Noris E., Portesi C. (2023). Raman-dielectrophoresis goes viral: Towards a rapid and label-free platform for plant virus characterization. Front. Microbiol..

[197] Saunders K., Thuenemann E.C., Shah S.N., Peyret H., Kristianingsih R., Lopez S.G., Richardson J., Lomonossoff G.P. (2022). The use of a replicating virus vector for in planta generation of tobacco mosaic virus nanorods suitable for metallization. Front. Bioeng. Biotechnol..

[198] Negrouk V., Eisner G., Midha S., Lee H.-i., Bascomb N., Gleba Y. (2004). Affinity purification of streptavidin using tobacco mosaic virus particles as purification tags. Anal. Biochem..

[199] Dobrov E.N., Nikitin N.A., Trifonova E.A., Parshina E.Y., Makarov V.V., Maksimov G.V., Karpova O.V., Atabekov J.G. (2014). β-structure of the coat protein subunits in spherical particles generated by tobacco mosaic virus thermal denaturation. J. Biomol. Struct. Dyn..

[200] Atabekov J., Nikitin N., Arkhipenko M., Chirkov S., Karpova O. (2011). Thermal transition of native tobacco mosaic virus and RNA-free viral proteins into spherical nanoparticles. J. Gen. Virol..

[201] Ambrico M., Ambrico P.F., Minafra A., De Stradis A., Vona D., Cicco S.R., Palumbo F., Favia P., Ligonzo T. (2016). Highly sensitive and practical detection of plant viruses via electrical impedance of droplets on textured silicon-based devices. Sensors.

[202] Sachse C., Chen J.Z., Coureux P.D., Stroupe M.E., Fandrich M., Grigorieff N. (2007). High-resolution electron microscopy of helical specimens: A fresh look at tobacco mosaic virus. J. Mol. Biol..

[203] Gregory J., Holmes K.C. (1965). Methods of preparing orientated tobacco mosaic virus sols for X-ray diffraction. J. Mol. Biol..

[204] Weis F., Beckers M., von der Hocht I., Sachse C. (2019). Elucidation of the viral disassembly switch of tobacco mosaic virus. EMBO Rep..

[205] Gooding G.V., Hebert T.T. (1967). A simple technique for purification of tobacco mosaic virus in large quantities. Phytopathology.

[206] Chapman S.N., Foster G.D., Taylor S.C. (1998). Tobamovirus Isolation and RNA Extraction. Plant Virology Protocols: From Virus Isolation to Transgenic Resistance.

[207] Martelli G.P., Russo M., Lauffer M.A., Bang F.B., Maramorosch K., Smith K.M. (1977). Plant virus inclusion bodies. Advances in Virus Research.

[208] Dierking I., Al-Zangana S. (2017). Lyotropic Liquid Crystal Phases from Anisotropic Nanomaterials. Nanomaterials.

[209] Bawden F.C., Sheffield F.M.L. (1939). The intracellular inclusions of some plant virus diseases. Ann. Appl. Biol..

[210] Wehrmeyer W. (1957). Darstellung und Strukturordnung eines Tabakmosalkvirus-Einschlußkörpers in der Zelle. Naturwissenschaften.

[211] Adams M.J., Adkins S., Bragard C., Gilmer D., Li D., MacFarlane S.A., Wong S.-M., Melcher U., Ratti C., Ryu K.H. (2017). ICTV virus taxonomy profile: Virgaviridae. J. Gen. Virol..

[212] Gibbs A.J. (1977). Tobamovirus group CMI/AAB descriptions of plant viruses. Assoc. Appl. Biol..

[213] Van Regenmortel M.H., Fraenkel-Conrat H., Van Regenmortel M.H., Fraenkel-Conrat H. (1986). The Plant Viruses: The Rod-Shaped Plant Viruses.

[214] Melcher U., Lewandowski D.J., Dawson W.O., Bamford D.H., Zuckerman M. (2021). Tobamoviruses (Virgaviridae). Encyclopedia of Virology.

[215] Higgins T.J., Goodwin P.B., Whitfeld P.R. (1976). Occurrence of short particles in beans infected with the cowpea strain of TMV. II. Evidence that short particles contain the cistron for coat-protein. Virology.

[216] Beachy R.N., Zaitlin M. (1977). Characterization and in vitro translation of the RNAs from less-than-full-length, virus-related, nucleoprotein rods present in tobacco mosaic virus preparations. Virology.

[217] Fukuda M., Meshi T., Okada Y., Otsuki Y., Takebe I. (1981). Correlation between particle multiplicity and location on virion RNA of the assembly initiation site for viruses of the tobacco mosaic virus group. Proc. Natl. Acad. Sci. USA.

[218] Kim S.-M., Lee J.-M., Yim K.-O., Oh M.-H., Park J.-W., Kim K.-H. (2003). Nucleotide sequences of two korean isolates of cucumber green mottle mosaic virus. Mol. Cells.

[219] Finch J.T. (1972). The hand of the helix of tobacco mosaic virus. J. Mol. Biol..

[220] Namba K., Pattanayek R., Stubbs G. (1989). Visualization of protein-nucleic acid interactions in a virus. Refined structure of intact tobacco mosaic virus at 2.9 A resolution by X-ray fiber diffraction. J. Mol. Biol..

[221] Ilyas R., Rohde M.J., Richert-Pöggeler K.R., Ziebell H. (2022). To be seen or not to be seen: Latent infection by tobamoviruses. Plants.

[222] Stubbs G. (1999). Tobacco mosaic virus particle structure and the initiation of disassembly. Philos. Trans. R. Soc. Lond. B.

[223] Culver J.N. (2002). Tobacco mosaic virus assembly and disassembly: Determinants in pathogenicity and resistance. Annu. Rev. Phytopathol..

[224] Wilson T.M.A., Perham R.N., Finch J.T., Butler P.J.G. (1976). Polarity of the RNA in the tobacco mosaic virus particle and the direction of protein stripping in sodium dodecyl sulphate. FEBS Lett..

[225] Goelet P., Lomonossoff G.P., Butler P.J., Akam M.E., Gait M.J., Karn J. (1982). Nucleotide sequence of tobacco mosaic virus RNA. Proc. Natl. Acad. Sci. USA.

[226] Wetter C., Conti M., Altschuh D., Tabillion R., Van Regenmortel M. (1984). Pepper mild mottle virus, a tobamovirus infecting pepper cultivars in Sicily. Phytopathology.

[227] Silber G., Burk L.G. (1965). Infectivity of tobacco mosaic virus stored for fifty years in extracted, ‘unpreserved’ plant juice. Nature.

[228] Fraile A., García-Arenal F., Palukaitis P., Roossinck M.J. (2018). Chapter four-Tobamoviruses as models for the study of virus evolution. Advances in Virus Research.

[229] Lartey R.T., Voss T.C., Melcher U. (1996). Tobamovirus evolution: Gene overlaps, recombination, and taxonomic implications. Mol. Biol. Evol..

[230] Min B.E., Chung B.N., Kim M.J., Ha J.H., Lee B.Y., Ryu K.H. (2006). Cactus mild mottle virus is a new cactus-infecting tobamovirus. Arch. Virol..

[231] Yamanaka T., Komatani H., Meshi T., Naito S., Ishikawa M., Ohno T. (1998). Complete nucleotide sequence of the genomic RNA of tobacco mosaic virus strain Cg. Virus Genes.

[232] Dombrovsky A., Smith E., Jimenez-Lopez J.C. (2017). Seed transmission of tobamoviruses: Aspects of global disease distribution. Advances in Seed Biology.

[233] Dombrovsky A., Tran-Nguyen L.T.T., Jones R.A.C. (2017). Cucumber green mottle mosaic virus: Rapidly increasing global distribution, etiology, epidemiology, and management. Annu. Rev. Phytopathol..

[234] Ishibashi K., Kubota K., Kano A., Ishikawa M. (2023). Tobamoviruses: Old and new threats to tomato cultivation. J. Gen. Plant Pathol..

[235] Anses (2020). Expert Committee on “Biological risks for plant health” ToBRFV Working Group Request No. 2019-SA-0080 ToBRFV Express pest risk analysis of tomato brown rugose fruit virus for France. Opin. Collect. Expert Apprais. Rep..

[236] Zhang S., Griffiths J.S., Marchand G., Bernards M.A., Wang A. (2022). Tomato brown rugose fruit virus: An emerging and rapidly spreading plant RNA virus that threatens tomato production worldwide. Mol. Plant Pathol..

[237] Salem N.M., Jewehan A., Aranda M.A., Fox A. (2023). Tomato brown rugose fruit virus pandemic. Annu. Rev. Phytopathol..

[238] Sui X., Li R., Shamimuzzaman M., Wu Z., Ling K.-S. (2019). Understanding the transmissibility of cucumber green mottle mosaic virus in watermelon seeds and seed health assays. Plant Dis..

[239] Liu H.W., Luo L.X., Li J.Q., Liu P.F., Chen X.Y., Hao J.J. (2014). Pollen and seed transmission of cucumber green mottle mosaic virus in cucumber. Plant Pathol..

[240] Matthews R.E. (1954). Effects of some purine analogues on tobacco mosaic virus. J. Gen. Microbiol..

[241] Commoner B., Shearer G.B., Yamada M. (1962). Linear biosynthesis of tobacco mosaic virus: Changes in rod length during the course of infection. Proc. Natl. Acad. Sci. USA.

[242] Kaper J.M., Siberg R.A. (1969). The effect of freezing on the structure of turnip yellow mosaic virus and a number of other simple plant viruses: An ultracentrifugal analysis. Cryobiology.

[243] Mundry K.-W. (1957). Die Abhängigkeit des Auftretens neuer Virusstämme von der Kulturtemperatur der Wirtspflanzen. Z. Indukt. Abstamm.-Vererbungslehre.

[244] Hebert T.T. (1963). Precipitation of plant viruses by polyethylene glycol. Phytopathology.

[245] Wetter C. (1985). Die Flüssigkristalle des Tabakmosaikvirus. Biol. Unserer Zeit.

[246] Zaitlin M. (2000). Tobacco mosaic virus CMI/AAB descriptions of plant viruses. Assoc. Appl. Biol..

[247] Bawden F., Pirie N. (1945). The separation and properties of tobacco mosaic virus in different states of aggregation. Br. J. Exp. Pathol..

[248] McNulty M.J., Schwartz A., Delzio J., Karuppanan K., Jacobson A., Hart O., Dandekar A., Giritch A., Nandi S., Gleba Y. (2022). Affinity sedimentation and magnetic separation with plant-made immunosorbent nanoparticles for therapeutic protein purification. Front. Bioeng. Biotechnol..

[249] Devash Y., Hauschner A., Sela I., Chakraburtty K. (1981). The antiviral factor (AVF) from virus-infected plants induces discharge of histidinyl-TMV-RNA. Virology.

[250] Zaitlin M., Israel H.W. (1975). Tobacco Mosaic Virus (Type Strain). AAB Descr. Plant Viruses.

[251] Stanley W.M. (1936). Chemical studies on the virus of tobacco mosaic. VI. The isolation from diseased Turkish tobacco plants of a crystalline protein possessing the properties of tobacco mosaic virus. Phytopathology.

[252] Leberman R. (1966). The isolation of plant viruses by means of “simple” coacervates. Virology.

[253] Yamamoto K.R., Alberts B.M., Benzinger R., Lawhorne L., Treiber G. (1970). Rapid bacteriophage sedimentation in the presence of polyethylene glycol and its application to large-scale virus purification. Virology.

[254] Juckles I.R.M. (1971). Fractionation of proteins and viruses with polyethylene glycol. Biochim. Biophys. Acta (BBA)-Protein Struct..

[255] Wyckoff R.W.G., Biscoe J., Stanley W.M. (1937). An ultracentrifugal analysis of the crystalline virus proteins isolated from plants diseased with different strains of tobacco mosaic virus. J. Biol. Chem..

[256] Mächtle W., Börger L. (2006). Analytical Ultracentrifugation of Polymers and Nanoparticles.

[257] McKinney H. (1927). Quantitative and purification methods in virus studies. J. Agric. Res..

[258] Wyckoff R.W.G., Corey R.B. (1936). The ultracentrifugal crystallization of tobacco mosaic virus protein. Science.

[259] Stanley W.M. (1937). Chemical studies on the virus of tobacco mosaic: X. The activity and yield of virus protein from plants diseased for different periods of time. J. Biol. Chem..

[260] Stanley W.M. (1937). Chemical studies on the virus of tobacco mosaic: IX. Correlation of virus activity and protein on centrifugation of protein from solution under various conditions. J. Biol. Chem..

[261] Stanley W.M., Wyckoff R.W.G. (1937). The isolation of tobacco ring spot and other virus proteins by ultracentrifugation. Science.

[262] Wyckoff R.W.G. (1937). An ultracentrifugal study of the pH stability of tobacco mosaic virus protein. J. Biol. Chem..

[263] Lauffer M.A. (1940). Ultracentrifugation studies on tobacco mosaic and bushy stunt viruses. J. Phys. Chem..

[264] Schachman H.K., Kauzmann W.J. (1949). Viscosity and sedimentation studies on tobacco mosaic virus. J. Phys. Colloid. Chem..

[265] Williams R.C., Steere R.L. (1951). Electron microscopic observations on the unit of length of the particles of tobacco mosaic virus. J. Am. Chem. Soc..

[266] Francki R., McLean G. (1968). Purification of potato virus X and preparation of infectious ribonucleic acid by degradation with lithium chloride. Aust. J. Biol. Sci..

[267] Bruening G., Beachy R.N., Scalla R., Zaitlin M. (1976). In vitro and in vivo translation of the ribonucleic acids of a cowpea strain of tobacco mosaic virus. Virology.

[268] Gardner R.C., Shepherd R.J. (1980). A procedure for rapid isolation and analysis of cauliflower mosaic virus DNA. Virology.

[269] Jensen S.M., Nguyen C.T., Jewett J.C. (2016). A gradient-free method for the purification of infective dengue virus for protein-level investigations. J. Virol. Methods.

[270] McAleer W.J., Hurni W., Wasmuth E., Hilleman M.R. (1979). High-resolution flow-zonal centrifuge system. Biotechnol. Bioeng..

[271] Brakke M.K. (1961). Density gradient centrifugation and its application to plant viruses. Advances in Virus Research.

[272] Brakke M.K. (1951). Density gradient centrifugation: A new separation technique. J. Am. Chem. Soc..

[273] Hills G.J. (1959). Partial crystallization of tobacco mosaic virus. Virology.

[274] Brakke M.K. (1964). Nonideal sedimentation and the capacity of sucrose gradient columns for virus in density-gradient centrifugation. Arch. Biochem. Biophys..

[275] Brakke M.K., Van Pelt N. (1970). Linear-log sucrose gradients for estimating sedimentation coefficients of plant viruses and nucleic acids. Anal. Biochem..

[276] Saunders K., Thuenemann E.C., Peyret H., Lomonossoff G.P. (2022). The tobacco mosaic virus origin of assembly sequence is dispensable for specific viral rna encapsidation but necessary for initiating assembly at a single site. J. Mol. Biol..

[277] Liu X., Wu F., Tian Y., Wu M., Zhou Q., Jiang S., Niu Z. (2016). Size Dependent Cellular Uptake of Rod-like Bionanoparticles with Different Aspect Ratios. Sci. Rep..

[278] Perham R.N. (1969). Sucrose density-gradient analysis of the alkaline degradation of tobacco mosaic virus. J. Mol. Biol..

[279] Siegel A., Hudson W. (1959). Equilibrium centrifugation of two strains of tobacco mosaic virus in density gradients. Biochim. Biophys. Acta.

[280] Gugerli P. (1984). Isopycnic centrifugation of plant viruses in Nycodenz^®^ density gradients. J. Virol. Methods.

[281] Skotnicki A., Scotti P.D., Gibbs A. (1976). On the nature of the difference in the densities of the particles of two tobamoviruses. Intervirology.

[282] Rickwood D., Ford T., Graham J. (1982). Nycodenz: A new nonionic iodinated gradient medium. Anal. Biochem..

[283] Rickwood D., Birnie G. (1975). Metrizamide, a new density-gradient medium. FEBS Lett..

[284] Pertoft H., Philipson L., Oxelfelt P., Höglund S. (1967). Gradient centrifugation of viruses in colloidal silica. Virology.

[285] Pertoft H. (2000). Fractionation of cells and subcellular particles with Percoll. J. Biochem. Biophys. Methods.

[286] Prucha M.J., Tanner F.W. (1920). Time saving bacteriological apparatus. J. Bacteriol..

[287] Arrua R.D., Strumia M.C., Alvarez Igarzabal C.I. (2009). Macroporous Monolithic Polymers: Preparation and Applications. Materials.

[288] Turpeinen D.G., Joshi P.U., Kriz S.A., Kaur S., Nold N.M., O’Hagan D., Nikam S., Masoud H., Heldt C.L. (2021). Continuous purification of an enveloped and non-enveloped viral particle using an aqueous two-phase system. Sep. Purif. Technol..

[289] McLean G.D., Francki R.I.B. (1967). Purification of lettuce necrotic yellows virus by column chromatography on calcium phosphate gel. Virology.

[290] Mahy B.W.J., Mahy B.W.J. (1985). Virology: A Practical Approach.

[291] Francki R., Zaitlin M., Grivell C. (1971). An unusual strain of tobacco mosaic virus from *Plumeria acutifolia*. Aust. J. Biol. Sci..

[292] Cochran G. (1947). A chromatographic method for the detection of tobacco-mosaic virus in juice from diseased Turkish tobacco plants. Phytopathology.

[293] Gray R.A. (1952). The electrophoresis and chromatography of plant viruses on filter paper. Arch. Biochem. Biophys..

[294] Barton R.J. (1977). An examination of permeation chromatography on columns of controlled pore glass for routine purification of plant viruses. J. Gen. Virol..

[295] Čech M., Jelinkova M., Čoupek J. (1977). High-pressure chromatography of tobacco mosaic virus on Spheron gels. J. Chromatogr..

[296] Brunt A., Phillips S., Jones R., Kenten R. (1982). Viruses detected in *Ullucus tuberosus* (Basellaceae) from Peru and Bolivia. Ann. Appl. Biol..

[297] Orita H., Sakai J.-I., Kubota K., Okuda M., Tanaka Y., Hanada K., Imamura Y., Nishiguchi M., Karasev A.V., Miyata S.-I. (2007). Molecular and serological characterization of cucumber mottle virus, a new cucurbit-infecting tobamo-like virus. Plant Dis..

[298] Cochran G.W., Chidester J.L., Stocks D.L. (1957). Chromatography of tobacco mosaic virus on a cellulose cation exchange adsorbent. Nature.

[299] Commoner B., Lippincott J.A., Shearer G.B., Richman E.E., Wu J.-H. (1956). Reconstitution of tobacco mosaic virus components. Nature.

[300] Levin Ö. (1958). Chromatography of tobacco mosaic virus and potato virus X. Arch. Biochem. Biophys..

[301] Ruščić J., Gutiérrez-Aguirre I., Tušek Žnidarič M., Kolundžija S., Slana A., Barut M., Ravnikar M., Krajačić M. (2015). A new application of monolithic supports: The separation of viruses from one another. J. Chromatogr..

[302] Krajacic M., Ravnikar M., Štrancar A., Gutiérrez-Aguirre I. (2017). Application of monolithic chromatographic supports in virus research. Electrophoresis.

[303] Kramberger P., Petrovič N., Štrancar A., Ravnikar M. (2004). Concentration of plant viruses using monolithic chromatographic supports. J. Virol. Methods.

[304] Kramberger P., Peterka M., Boben J., Ravnikar M., Štrancar A. (2007). Short monolithic columns-A breakthrough in purification and fast quantification of tomato mosaic virus. J. Chromatogr..

[305] Guilley H., Stussi C., Thouvenel J.C., Pfeiffer P., Hirth L. (1972). Hydroxyapatite column chromatography of reconstitution products of TMV: Some properties of the isolated fractions. Virology.

[306] Townsley P.M. (1961). Chromatography of tobacco mosaic virus (TMV) on chitin columns. Nature.

[307] Kubo S., Tomaru K., Nitta T., Shiroya T., Hidaka Z. (1965). Chromatography of tobacco mosaic virus, its constituents, and nucleic acids extracted from infected tobacco leaf tissues. Virology.

[308] Fraser D., Johnson F. (1949). The influence of buffer composition, pH and aggregation on the thermal denaturation of tobacco mosaic virus. Arch. Biochem..

[309] Desjardins P.R., French J.V. (1969). Storage of purified plant viruses in the unfrozen state free of microbial contamination. Experientia.

[310] Best R.J. (1948). Longevity of tobacco mosaic virus: Part I. in vitro life of the pure virus in buffer solution at pH 4. Aust. J. Exp. Biol. Med. Sci..

[311] Byrne N., Rodoni B., Constable F., Varghese S., Davis J.H. (2012). Enhanced stabilization of the tobacco mosaic virus using protic ionic liquids. Phys. Chem. Chem. Phys..

[312] Rice R.V., Kaesberg P., Stahmann M.A. (1956). Further studies concerning the breaking of tobacco mosaic virus. Biochim. Biophys. Acta.

[313] Donev T., Yordanova A., Stoimenova E., Damjanova S. (1996). Determination of parameters for tobacco mosaic virus cryogenic treatment and freeze-drying. Biotechnol. Tech..

[314] Wacker W.E., Gordon M.P., Huff J.W. (1963). Metal content of tobacco mosaic virus and tobacco mosaic virus RNA. Biochemistry.

[315] Hulett H.R., Loring H.S. (1965). Effect of particle length distribution on infectivity of tobacco mosaic virus. Virology.

[316] Loring H.S., Fujimoto Y., Tu A.T. (1962). Tobacco mosaic virus—A calcium-magnesium coordination complex. Virology.

[317] Ladipo J.L., Koenig R., Lesemann D.E. (2003). Nigerian tobacco latent virus: A new tobamovirus from tobacco in Nigeria. Eur. J. Plant Pathol..

[318] Whitfeld P.R., Higgins T.J. (1976). Occurrence of short particles in beans infected with the cowpea strain of TMV. I. Purification and characterization of short particles. Virology.

[319] Eskelin K., Poranen M.M., Oksanen H.M. (2019). Asymmetrical flow field-flow fractionation on virus and virus-like particle applications. Microorganisms.

[320] Lampi M., Oksanen H.M., Meier F., Moldenhauer E., Poranen M.M., Bamford D.H., Eskelin K. (2018). Asymmetrical flow field-flow fractionation in purification of an enveloped bacteriophage ϕ6. J. Chromatogr. B.

[321] Albertsson P.A., Frick G. (1960). Partition of virus particles in a liquid two-phase system. Biochim. Biophys. Acta.

[322] Teepakorn C., Fiaty K., Charcosset C. (2016). Comparison of Membrane Chromatography and Monolith Chromatography for Lactoferrin and Bovine Serum Albumin Separation. Processes.

[323] Yang Z., Xu X., Silva C.A.T., Farnos O., Venereo-Sanchez A., Toussaint C., Dash S., González-Domínguez I., Bernier A., Henry O. (2022). Membrane chromatography-based downstream processing for cell-culture produced influenza vaccines. Vaccines.

[324] Chen J., Yu B., Cong H., Shen Y. (2023). Recent development and application of membrane chromatography. Anal. Bioanal. Chem..

[325] Sartorius (2021). Resins, monoliths, or membranes. Which chromatographic method should you use?. Sartorius AG Sci.-Snippets.

[326] Thuenemann E.C., Le D.H.T., Lomonossoff G.P., Steinmetz N.F. (2021). Bluetongue virus particles as nanoreactors for enzyme delivery and cancer therapy. Mol. Pharmacol..

[327] Thuenemann E.C., Meyers A.E., Verwey J., Rybicki E.P., Lomonossoff G.P. (2013). A method for rapid production of heteromultimeric protein complexes in plants: Assembly of protective bluetongue virus-like particles. Plant Biotechnol. J..

[328] Dennis S.J., Meyers A.E., Guthrie A.J., Hitzeroth I.I., Rybicki E.P. (2018). Immunogenicity of plant-produced African horse sickness virus-like particles: Implications for a novel vaccine. Plant Biotechnol. J..

[329] Ford T., Graham J., Rickwood D. (1994). Iodixanol: A nonionic iso-osmotic centrifugation medium for the formation of self-generated gradients. Anal. Biochem..

[330] Rybicki E.P. (2020). Plant molecular farming of virus-like nanoparticles as vaccines and reagents. WIREs Nanomed. Nanobiotechnol..

[331] Marsian J., Lomonossoff G.P. (2016). Molecular pharming—VLPs made in plants. Curr. Opin. Biotechnol..

[332] Zahmanova G., Aljabali A.A., Takova K., Toneva V., Tambuwala M.M., Andonov A.P., Lukov G.L., Minkov I. (2023). The Plant Viruses and Molecular Farming: How Beneficial They Might Be for Human and Animal Health?. Int. J. Mol. Sci..

[333] Margolin E., Allen J.D., Verbeek M., Van Diepen M., Ximba P., Chapman R., Meyers A., Williamson A.-L., Crispin M., Rybicki E. (2021). Site-specific glycosylation of recombinant viral glycoproteins produced in Nicotiana benthamiana. Front. Plant Sci..

[334] Srivastava A., Mallela K.M.G., Deorkar N., Brophy G. (2021). Manufacturing challenges and rational formulation development for AAV viral vectors. J. Pharm. Sci..

[335] Moleirinho M.G., Silva R.J.S., Alves P.M., Carrondo M.J.T., Peixoto C. (2020). Current challenges in biotherapeutic particles manufacturing. Expert Opin. Biol. Ther..

[336] Gilleskie G., Rutter C., McCuen B. (2021). Biopharmaceutical Manufacturing: Principles, Processes, and Practices.

