# Supplementary material for: Getting Hold of the Tobamovirus Particle—Why and How? Purification Routes over Time and a New Customizable Approach
Source: Viruses. 2024 May 30;16(6):884. doi: 10.3390/v16060884 (PMC11209083; doi:10.3390/v16060884)
Supplement: Supplementary file 1 [file viruses-16-00884-s001.zip › viruses-2968107-supplementary.pdf]

viruses

MDPI

Supplementary Materials

Getting Hold of the Tobamovirus Particle - Why and How? Purification Routes Over Time and a New Customizable Approach

Tim Wendlandt, Beate Britz, Tatjana Kleinow [a], Katharina Hipp [a], Fabian Eber [a] and Christina Wege *

[a] These authors contributed equally to the work. They have the right to exchange the order of their names freely within the range of the current positions in their scientific CV.

* Correspondence: Christina Wege, Research Unit Molecular and Synthetic Plant Virology, Institute of Biomaterials and Biomolecular Systems, University of Stuttgart, 70569 Stuttgart, Germany, email: christina.wege@bio.uni-stuttgart.de

Supplementary Materials:

Supplementary Table S1.
Historical overview of the first purification methods described for virions in different tobamovirus species.
References within Table S1.

Supplementary Materials S2 Corresponding to Section 4.3: The Established and the New - A Case Study. Additional Original Data and Methods.

S2.1 Buoyant Density of Virus Particles in Iodixanol-Containing Media

S2.2. Lengths of Virions Isolated via Three-Layer Density Barrier Phase Systems

S2.3. IgG-Binding of $TVCV_{PA}$ Adsorbed from Iodixanol Media to Solid Supports

S2.4. Isolation of $TVCV_{(PA)}$ via Iodixanol Density Barrier Phase Systems - Detailed Method

Reference in Supplementary Materials S2.

Supplementary Table S1. Historical overview of the first* purification methods described for virions in different tobamovirus species. Note that particles of numerous species can be purified using various procedures, including ultracentrifugation, exclusion chromatography, as well as precipitation methods employing salt, polyethylene glycol (PEG), pH according to isoelectric point, or solvents. References refer to list below.

**Purification method** (sorted by subsequent steps for concentration & further purification)			**Viruses**	**Abbreviation**	**Reference First particle purification of species**
**Reference Method**	**Initial steps clarification non-viral component removal**	**Subsequent steps concentration further purification**			
**Salt precipitation**					
[1]	Salt precipitation $(NH_4)_2SO_4$<br>flow-through chromatography (Celite)	Salt precipitation $(NH_4)_2SO_4$<br>Acid precipitation	Tobacco mosaic virus	TMV	[2]<br>[3]<br>[4]
[5]	Butanol-chloroform emulsion<br>Low-speed centrifugation	Salt precipitation $(NH_4)_2SO_4$	Tomato mosaic virus<br>Ribgrass mosaic virus<br>Sunn-hemp mosaic virus<br>Tobacco mild green mosaic virus [1]<br>Cucumber green mottle mosaic virus [2]	ToMV<br>RMV<br>SHMV<br>TMGMV<br>CGMMV	[5]
**Centrifugation**					
[5] modified according to [6]	Freeze-thaw coagulation<br>flow-through chromatography (Celite)<br>Low-speed centrifugation	3 cycles of low- and high-speed centrifugation	Tomato mosaic virus<br>Ribgrass mosaic virus<br>Sunn-hemp mosaic virus<br>Tobacco mild green mosaic virus [1]<br>Cucumber green mottle mosaic virus [2]	ToMV<br>RMV<br>SHMV<br>TMGMV<br>CGMMV	[5]
[7]<br>[8]	Charcoal & DEAE-cellulose<br>flow-through chromatography (Celite)<br>Centrifugation 16,000 x *g*	Centrifugation 44,000 x *g*<br>2 cycles of:<br>Chloroform emulsion<br>Low speed centrifugation<br>Centrifugation 160,000 x *g*	Frangipani mosaic virus	FrMV	[9]<br>[10]<br>[11]

[3]<br>[12]	Butanol emulsion<br>Low speed centrifugation	Differential centrifugation<br>Density gradient centrifugation (Sucrose or CsCl)<br>alternatively Chromatography (controlled-pore glass beads)	Ullucus mild mottle virus	UMMV	[10]<br>[11]<br>[13]
[14]	Butanol-chloroform emulsion<br>Low speed centrifugation	Differential centrifugation	Odontoglossum ringspot virus	ORSV	[15]
[16]	Filtration (Muslin)<br>Centrifugation	Differential centrifugation	Sunn-hemp mosaic virus	SHMV	[17]
[18]	Chloroform emulsion<br>Low speed centrifugation	Differential centrifugation	Cucumber green mottle mosaic virus	CGMMV	[18]
[19]	Differential centrifugation<br>Butanol-chloroform emulsion	Differential centrifugation	Cucumber fruit mottle mosaic virus	CFMMV	[20]
[21]	Freeze-thaw coagulation<br>Filtration<br>Low speed centrifugation<br>Triton X-100 treatment	Differential centrifugation (Sucrose cushion)<br>Density gradient centrifugation (Sucrose)	Tobacco mild green mosaic virus	TMGMV	[22]
[23] modified according to [24]	Triton X-100 treatment<br>Low speed centrifugation	High-speed centrifugation (Sucrose cushion)	Turnip vein-clearing virus	TVCV	[25]<br>[26]
[27]	Low speed centrifugation<br>Butanol-chloroform emulsion	Density gradient centrifugation (CsCl)	Hibiscus latent Singapore virus	HLSV	[27]
		**Polyethylenglycol (PEG) precipitation**			
[28]	Low speed centrifugation<br>Heat coagulation	PEG / NaCl precipitation & centrifugation	Hibiscus latent Fort Pierce virus	HLFPV	[29]<br>[30]
[31]	Filtration (Cheesecloth)<br>Butanol emulsion<br>Low speed centrifugation	PEG / NaCl precipitation & centrifugation<br>Clarifying centrifugation 10,000 x *g*	Watermelon green mottle mosaic virus [3]	WGMMV	[32]
See [31]			Rehmannia mosaic virus	RheMV	[33]

See [31]		Modified clarifying centrifugation 78,000 x *g*	Odontoglossum ringspot virus	ORSV	[34]<br>[35]
See [31]		Modified clarifying centrifugation 105,000 x *g*	Brugmansia mild mottle virus	BrMMV	[36]
[5]	Butanol-chloroform emulsion<br>Low speed centrifugation	PEG / NaCl precipitation & centrifugation	Tomato mosaic virus<br>Ribgrass mosaic virus<br>Sunn-hemp mosaic virus<br>Tobacco mild green mosaic virus [1]<br>Cucumber green mottle mosaic virus [2]	ToMV<br>RMV<br>SHMV<br>TMGMV<br>CGMMV	[5]
See [5]			Tropical soda apple mosaic virus	TSAMV	[37]<br>[38]
See [5]			Hoya chlorotic spot virus [3]	HoCSV	[39]<br>[38]
[40]	Filtration (Miracloth)<br>Butanol emulsion<br>Low speed centrifugation	PEG / NaCl precipitation & centrifugation<br>10,000 x *g* (2 cycles)<br>Clarifying centrifugation 13,000 x *g*	Zucchini green mottle mosaic virus	ZGMMV	[41]
See [40]			Kyuri green mottle mosaic virus	KGMMV	[42]
See [40]			Cactus mild mottle virus	CMMoV	[43]
See [40]			Passion fruit mosaic virus	PFMV	[44]
		**Combinations**			
[45]<br>[28]	Low speed centrifugation<br>Heat coagulation	PEG / NaCl precipitation & centrifugation<br>Differential centrifugation (2-3 cycles)	Bell pepper mottle virus	BPMV	[45]
[46] [4]	Filtration (Miracloth)<br>Butanol emulsion<br>Low speed centrifugation<br><br>according to [19]	PEG / NaCl precipitation & centrifugation<br>Clarify centrifugation 10,000 x *g*<br>Chromatography (controlled-pore glass beads)<br>Ethanol precipitation	Cucumber mottle virus	CMoV	[47]

[48]	Not specified	PEG / NaCl precipitation & centrifugation<br>Differential centrifugation (2 cycles)	Obuda pepper virus	ObPV	[49]
See [48]			Wasabi mottle virus	WMoV	[50]
[51] modified	Butanol-chloroform emulsion<br>Low speed centrifugation	PEG / NaCl precipitation & centrifugation<br>Differential centrifugation (2 cycles)	Maracuja mosaic virus	MarMV	[52]
[53]	Filtration (Cheesecloth)<br>Butanol emulsion<br>Low-speed centrifugation (2 cycles with intermediate storage o/n at 4°C)	PEG / NaCl precipitation & centrifugation<br>Clarifying centrifugation 10,000 x *g* (2 cycles)<br>Triton X-100 treatment<br>Differential centrifugation<br>Density gradient centrifugation (Sucrose)	Streptocarpus flower break virus	SFBV	[54]
See [53]			Pepper mild mottle virus	PMMoV	[53] [5]
[55]<br>[24]	$AgNO_3$ treatment<br>Chloroform emulsion<br>Low speed centrifugation	PEG / NaCl precipitation & centrifugation<br>Triton X-100 treatment<br>Differential centrifugation (1-2 cycles)<br>Density gradient centrifugation (Sucrose)	Tobacco latent virus	TLV1	[56]
[57]<br>[58]	Chloroform emulsion<br>Low speed centrifugation	PEG / NaCl precipitation & centrifugation<br>Differential centrifugation<br>Density gradient centrifugation (Sucrose) (2 cycles)	Paprika mild mottle virus	PaMMV	[59]

[*] The articles referenced as first reports of purification protocols for virions in various tobamovirus species were selected to the best of our knowledge.
[1] here not yet considered as TMGMV (para TMV strain); [2] here not yet considered as CGMMV (cucumber 4 TMV strain); [3] tentative species related but yet unclassified: https://ictv.global/report/chapter/virgaviridae/virgaviridae/tobamovirus; [4] reference gives two different protocols; [5] not yet considered here as PMMoV (Paprika strain of TMV), the name PMMoV was later proposed by Wetter et al [60], in [60] the virus particles were purified according to modified PEG precipitation protocol [6].

References within Table S1.

1. Stanley, W.M. Isolation of a crystalline protein possessing the properties of tobacco mosaic virus. *Science* **1935**, *81*, 644-645, doi:10.1126/science.81.2113.644.
2. Stanley, W.M. Chemical studies on the virus of tobacco mosaic. VI. The isolation from diseased Turkish tobacco plants of a crystalline protein possessing the properties of tobacco mosaic virus. *Phytopathology* **1936**, *26*, 305-320.
3. Steere, R.L. The purification of plant viruses. In *Adv. Virus Res.*, Smith, K.M., Lauffer, M.A., Eds.; Academic Press: 1959; Volume 6, pp. 1-73.
4. Kay, L.E. W. M. Stanley's crystallization of the tobacco mosaic virus, 1930-1940. *Isis* **1986**, *77*, 450-472, doi:10.1086/354205.
5. Kreibig, U.; Wetter, C. Light diffraction of in vitro crystals of six tobacco mosaic viruses. *Z. Naturforsch. Sect. C* **1980**, *35*, 750-762, doi:10.1515/znc-1980-9-1017.
6. Boedtker, H.; Simmons, N.S. The preparation and characterization of essentially uniform tobacco mosaic virus particles. *J. Am. Chem. Soc.* **1958**, *80*, 2550-2556, doi:10.1021/ja01543a049.
7. Francki, R.; McLean, G. Purification of potato virus X and preparation of infectious ribonucleic acid by degradation with lithium chloride. *Australian J. biol. Sci.* **1968**, *21*, 1311-1318, doi:10.1071/bi9681311.
8. McLean, G.D.; Francki, R.I.B. Purification of lettuce necrotic yellows virus by column chromatography on calcium phosphate gel. *Virology* **1967**, *31*, 585-591, doi:10.1016/0042-6822(67)90186-9.
9. Francki, R.; Zaitlin, M.; Grivell, C. An unusual strain of tobacco mosaic virus from *Plumeria acutifolia*. *Australian J. biol. Sci.* **1971**, *24*, 811-814, doi:10.1071/BI9710815.
10. Varma, A.; Gibbs, A.J. Frangipani mosaic virus CMI/AAB descriptions of plant viruses. *Association of Applied Biologicts* **1978**, *196*.
11. Brunt, A.A. Miscellaneous tobamoviruses. In *The plant viruses: The rod-shaped plant viruses*, Van Regenmortel, M.H.V., Fraenkel-Conrat, H., Eds.; Springer US: Boston, MA, 1986; pp. 283-302.
12. Brunt, A.; Phillips, S.; Jones, R.; Kenten, R. Viruses detected in *Ullucus tuberosus* (Basellaceae) from Peru and Bolivia. *Ann. Appl. Biol.* **1982**, *101*, 65-71, doi:10.1111/j.1744-7348.1982.tb00801.x.
13. Offei, S.K.; Ibidapo, O.; Brunt, A.A.; Coutts, R.H.A. Further evidence for the recognition of Ullucus mild mottle virus as a distinct tobamovirus. *J. Phytopathol.* **1995**, *143*, 543-545, doi:10.1111/j.1439-0434.1995.tb00660.x.
14. Paul, H.L.; Wetter, C.; Wittmann, H.G.; Brandes, J. Untersuchungen am Odontoglossum ringspot Virus, einem Verwandten des Tabakmosaik-Virus. *Z. Vererbungsl.* **1965**, *97*, 186-203, doi:10.1007/BF00897495.
15. Paul, H.L. Odontoglossum ringspot virus CMI/AAB descriptions of plant viruses. *Association of Applied Biologicts* **1975**, *155*.
16. Kassanis, B.; McCarthy, D. The quality of virus as affected by the ambient temperature. *J. Gen. Virol.* **1967**, *1*, 425-440, doi:10.1099/0022-1317-1-4-425.
17. Kassanis, B.; Vama, A. Sunn-hemp mosaic virus CMI/AAB descriptions of plant viruses. *Association of Applied Biologicts* **1975**, *153*.
18. Nozu, Y.; Tochihara, H.; Komuro, Y.; Okada, Y. Chemical and immunological characterization of cucumber green mottle mosaic virus (watermelon strain) protein. *Virology* **1971**, *45*, 577-585, doi:10.1016/0042-6822(71)90173-5.
19. Tung, J.-S.; Knight, C.A. The coat protein subunits of cucumber viruses 3 and 4 and a comparison of methods for determining their molecular weights. *Virology* **1972**, *48*, 574-581, doi:10.1016/0042-6822(72)90068-2.
20. Antignus, Y.; Wang, Y.; Pearlsman, M.; Lachman, O.; Lavi, N.; Gal-On, A. Biological and molecular characterization of a new cucurbit-infecting tobamovirus. *Phytopathology* **2001**, *91*, 565-571, doi:10.1094/phyto.2001.91.6.565.
21. Bruening, G.; Beachy, R.N.; Scalla, R.; Zaitlin, M. In vitro and in vivo translation of the ribonucleic acids of a cowpea strain of tobacco mosaic virus. *Virology* **1976**, *71*, 498-517, doi:10.1016/0042-6822(76)90377-9.

22. Fraile, A.; Escriu, F.; Aranda, M.A.; Malpica, J.M.; Gibbs, A.J.; García-Arenal, F. A century of tobamovirus evolution in an Australian population of *Nicotiana glauca*. *J. Virol.* **1997**, *71*, 8316-8320, doi:10.1128/jvi.71.11.8316-8320.1997.
23. Gardner, R.C.; Shepherd, R.J. A procedure for rapid isolation and analysis of cauliflower mosaic virus DNA. *Virology* **1980**, *106*, 159-161, doi:10.1016/0042-6822(80)90234-2.
24. Hull, R.; Shepherd, R.J.; Harvey, J.D. Cauliflower mosaic virus: an improved purification procedure and some properties of the virus particles. *J. Gen. Virol.* **1976**, *31*, 93-100, doi:10.1099/0022-1317-31-1-93.
25. Lartey, R.T.; Hartson, S.D.; Pennington, R.E.; Sherwood, J.L.; Melcher, U. Occurrence of a vein-clearing tobamovirus in turnip. *Plant Dis.* **1993**, *77*, 21-24, doi:10.1094/Pd-77-0021.
26. Lartey, R.T.; Lane, L.C.; Melcher, U. Electron microscopic and molecular characterization of turnip vein-clearing virus. *Arch. Virol.* **1994**, *138*, 287-298, doi:10.1007/BF01379132.
27. Srinivasan, K.G.; Narendrakumar, R.; Wong, S.M. Hibiscus virus S is a new subgroup II tobamovirus: evidence from its unique coat protein and movement protein sequences. *Arch. Virol.* **2002**, *147*, 1585-1598, doi:10.1007/s00705-002-0829-z.
28. Hebert, T.T. Precipitation of plant viruses by polyethylene glycol. *Phytopathology* **1963**, *53*, 362.
29. Yoshida, T.; Kitazawa, Y.; Komatsu, K.; Neriya, Y.; Ishikawa, K.; Fujita, N.; Hashimoto, M.; Maejima, K.; Yamaji, Y.; Namba, S. Complete nucleotide sequence and genome structure of a Japanese isolate of hibiscus latent Fort Pierce virus, a unique tobamovirus that contains an internal poly(A) region in its 3′ end. *Arch. Virol.* **2014**, *159*, 3161-3165, doi:10.1007/s00705-014-2175-3.
30. Watanabe, T.; Honda, A.; Iwata, A.; Ueda, S.; Hibi, T.; Ishihama, A. Isolation from tobacco mosaic virus-infected tobacco of a solubilized template-specific RNA-dependent RNA polymerase containing a 126K/183K protein heterodimer. *J. Virol.* **1999**, *73*, 2633-2640, doi:10.1128/jvi.73.4.2633-2640.1999.
31. Gooding, G.V., Jr.; Hebert, T.T. A simple technique for purification of tobacco mosaic virus in large quantities. *Phytopathology* **1967**, *57*, 1285.
32. Cheng, Y.H.; Huang, C.H.; Chang, C.J.; Jan, F.J. Identification and characterisation of watermelon green mottle mosaic virus as a new cucurbit-infecting tobamovirus. *Ann. Appl. Biol.* **2019**, *174*, 31-39, doi:10.1111/aab.12467.
33. Zhang, Z.C.; Lei, C.Y.; Zhang, L.F.; Yang, X.X.; Chen, R.; Zhang, D.S. The complete nucleotide sequence of a novel tobamovirus, Rehmannia mosaic virus. *Arch. Virol.* **2008**, *153*, 595-599, doi:10.1007/s00705-007-0002-9.
34. Ryu, K.H.; Choi, C.W.; Choi, J.K.; Park, W.M. Cloning of the 3′-terminal region encoding movement and coat proteins of a Korean isolate of Odontoglossum ringspot virus. *Arch. Virol.* **1995**, *140*, 481-490, doi:10.1007/BF01718425.
35. Park, W.; Yoon, K.; Chung, S.; Ryu, K. Purification and serological detection of Odontoglossum ringspot virus isolated from *Cymbidium goeringii* in Korea. *Korean J. Plant Pathol.* **1990**, *6*, 474-481.
36. Ilmberger, N.; Willingmann, P.; Adam, G.; Heinze, C. A subgroup 1 tobamovirus isolated from *Brugmansia sp*. and its detection by RT-PCR. *J. Phytopathol.* **2007**, *155*, 326-332, doi:10.1111/j.1439-0434.2007.01235.x.
37. Adkins, S.; Kamenova, I.; Rosskopf, E.N.; Lewandowski, D.J. Identification and characterization of a novel tobamovirus from tropical soda apple in Florida. *Plant Dis.* **2007**, *91*, 287-293, doi:10.1094/PDIS-91-3-0287.
38. Wetter, C.; Conti, M. Pepper mild mottle virus CMI/AAB descriptions of plant viruses. *Association of Applied Biologicts* **1988**, *330*.
39. Adkins, S.; D′Elia, T.; Fillmer, K.; Pongam, P.; Baker, C.A. Biological and genomic characterization of a novel tobamovirus infecting *Hoya spp*. *Plant Dis.* **2018**, *102*, 2571-2577, doi:10.1094/pdis-04-18-0667-re.
40. Chapman, S.N. Tobamovirus isolation and RNA extraction. In *Plant Virology Protocols: From Virus Isolation to Transgenic Resistance*, Foster, G.D., Taylor, S.C., Eds.; Humana Press: Totowa, NJ, 1998; pp. 123-129.

41. Ryu, K.H.; Min, B.E.; Choi, G.S.; Choi, S.H.; Kwon, S.B.; Noh, G.M.; Yoon, J.Y.; Choi, Y.M.; Jang, S.H.; Lee, G.P.; et al. Zucchini green mottle mosaic virus is a new tobamovirus; comparison of its coat protein gene with that of Kyuri green mottle mosaic virus. *Arch. Virol.* **2000**, *145*, 2325-2333, doi:10.1007/s007050070023.
42. Yoon, J.Y.; Min, B.E.; Choi, S.H.; Ryu, K.H. Completion of nucleotide sequence and generation of highly infectious transcripts to cucurbits from full-length cDNA clone of Kyuri green mottle mosaic virus. *Arch. Virol.* **2001**, *146*, 2085-2096, doi:10.1007/s007050170022.
43. Min, B.E.; Chung, B.N.; Kim, M.J.; Ha, J.H.; Lee, B.Y.; Ryu, K.H. Cactus mild mottle virus is a new cactus-infecting tobamovirus. *Arch. Virol.* **2006**, *151*, 13-21, doi:10.1007/s00705-005-0617-7.
44. Song, Y.S.; Ryu, K.H. The complete genome sequence and genome structure of passion fruit mosaic virus. *Arch. Virol.* **2011**, *156*, 1093-1095, doi:10.1007/s00705-011-0961-8.
45. Wetter, C.; Dore, I.; Bernard, M. Bell pepper mottle virus, a distinct tobamovirus infecting pepper. *J. Phytopathol.* **1987**, *119*, 333-344, doi:10.1111/j.1439-0434.1987.tb04404.x.
46. Hollings, M.; Komuro, Y.; Tochihara, H. Cucumber green mottle mosaic virus CMI/AAB descriptions of plant viruses. *Association of Applied Biologicts* **1975**, *154*.
47. Orita, H.; Sakai, J.-I.; Kubota, K.; Okuda, M.; Tanaka, Y.; Hanada, K.; Imamura, Y.; Nishiguchi, M.; Karasev, A.V.; Miyata, S.-I. Molecular and serological characterization of cucumber mottle virus, a new cucurbit-infecting tobamo-like virus. *Plant Dis.* **2007**, *91*, 1574-1578, doi:10.1094/PDIS-91-12-1574.
48. Otsuki, Y.; Takebe, I.; Ohno, T.; Fukuda, M.; Okada, Y. Reconstitution of tobacco mosaic virus rods occurs bidirectionally from an internal initiation region: Demonstration by electron microscopic serology. *Proc. Natl. Acad. Sci. USA* **1977**, *74*, 1913-1917, doi:10.1073/pnas.74.5.1913.
49. Ikeda, R.; Watanabe, E.; Watanabe, Y.; Okada, Y. Nucleotide sequence of tobamovirus Ob which can spread systemically in N gene tobacco. *J. Gen. Virol.* **1993**, *74*, 1939-1944, doi:10.1099/0022-1317-74-9-1939.
50. Shimamoto, I.; Sonoda, S.; Vazquez, P.; Minaka, N.; Nishiguchi, M. Nucleotide sequence analysis of the 3′ terminal region of a wasabi strain of crucifer tobamovirus genomic RNA: subgrouping of crucifer tobamoviruses. *Arch. Virol.* **1998**, *143*, 1801-1813, doi:10.1007/s007050050418.
51. Fribourg, C.; Nakashima, J. Characterization of a new potyvirus from potato. *Phytopathology* **1984**, *74*, 1363-1369, doi:10.1094/Phyto-74-1363.
52. Fribourg, C.; Koenig, R.; Lesemann, D. A new tobamovirus from *Passiflora edulis* in Peru. *Phytopathology* **1987**, *77*, 486-491, doi:10.1094/Phyto-77-486.
53. Tóbiás, I.; Rast, A.T.B.; Maat, D.Z. Tobamoviruses of pepper, eggplant and tobacco: Comparative host reactions and serological relationships. *Neth. J. Pl. Path.* **1982**, *88*, 257-268, doi:10.1007/BF02000131.
54. Verhoeven, J.T.J.; Bouwen, I.; Roenhorst, J.W. A new flower breaking tobamovirus of Streptocarpus. *Eur. J. Plant Pathol.* **1995**, *101*, 311-318, doi:10.1007/BF01874787.
55. Koenig, R.; Lesemann, D.-E.; Lockhart, B.; Betzold, J.A.; Weidemann, H.L. Natural occurrence of Helenium virus S in *Impatiens holstii*. *J. Phytopathol.* **1983**, *106*, 133-140, doi:10.1111/j.1439-0434.1983.tb00036.x.
56. Ladipo, J.L.; Koenig, R.; Lesemann, D.E. Nigerian tobacco latent virus: a new tobamovirus from tobacco in Nigeria. *Eur. J. Plant Pathol.* **2003**, *109*, 373-379, doi:10.1023/A:1023557723943.
57. Takeuchi, S.; Hikichi, Y.; Kawada, Y.; Okuno, T. Detection of tobamoviruses from soils by non-precoated indirect ELISA. *J. Gen. Plant Pathol.* **2000**, *66*, 153-158, doi:10.1007/PL00012938.
58. Takeuchi, S.; Hikichi, Y.; Kawada, Y.; Okuno, T. Direct immunostaining assay, a new simplified technique for detection of tobamoviruses from seeds of green pepper (*Capsicum annuum* L.). *Japanese J. Phytopathol.* **1999**, *65*, 189-191, doi:10.3186/jjphytopath.65.189.

59. Hamada, H.; Takeuchi, S.; Morita, Y.; Sawada, H.; Kiba, A.; Hikichi, Y. Characterization of paprika mild mottle virus first isolated in Japan. *J. Gen. Plant Pathol.* **2003**, *69*, 199-204, doi:10.1007/s10327-002-0028-5.
60. Wetter, C.; Conti, M.; Altschuh, D.; Tabillion, R.; Van Regenmortel, M. Pepper mild mottle virus, a tobamovirus infecting pepper cultivars in Sicily. *Phytopathology* **1984**, *74*, 405-410, doi:10.1094/Phyto-74-405.

Supplementary Materials S2 Corresponding to Section 4.3: The Established and the New - A Case Study. Additional Original Data and Methods

S2.1 Buoyant Density of Virus Particles in Iodixanol-Containing Media

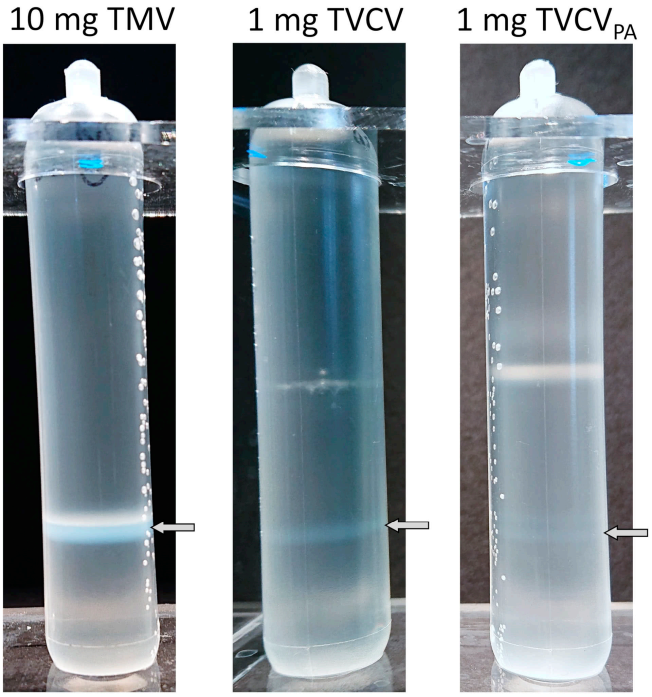

Figure S1. Determination of the buoyant densities of TMV, TVCV, and TVCV$_{PA}$ by isopycnic centrifugation. Centrifuge tubes (Seton Scientific VTi 65.1) were filled with 30% iodixanol solution and loaded with 10 mg TMV or 1 mg TVCV/TVCV$_{PA}$ per tube, as indicated. Gray arrows: virus bands following centrifugation (15 °C, 6 h, r_{max} 288,000 × *g*).

S2.2. Lengths of Virions Isolated via Three-Layer Density Barrier Phase Systems

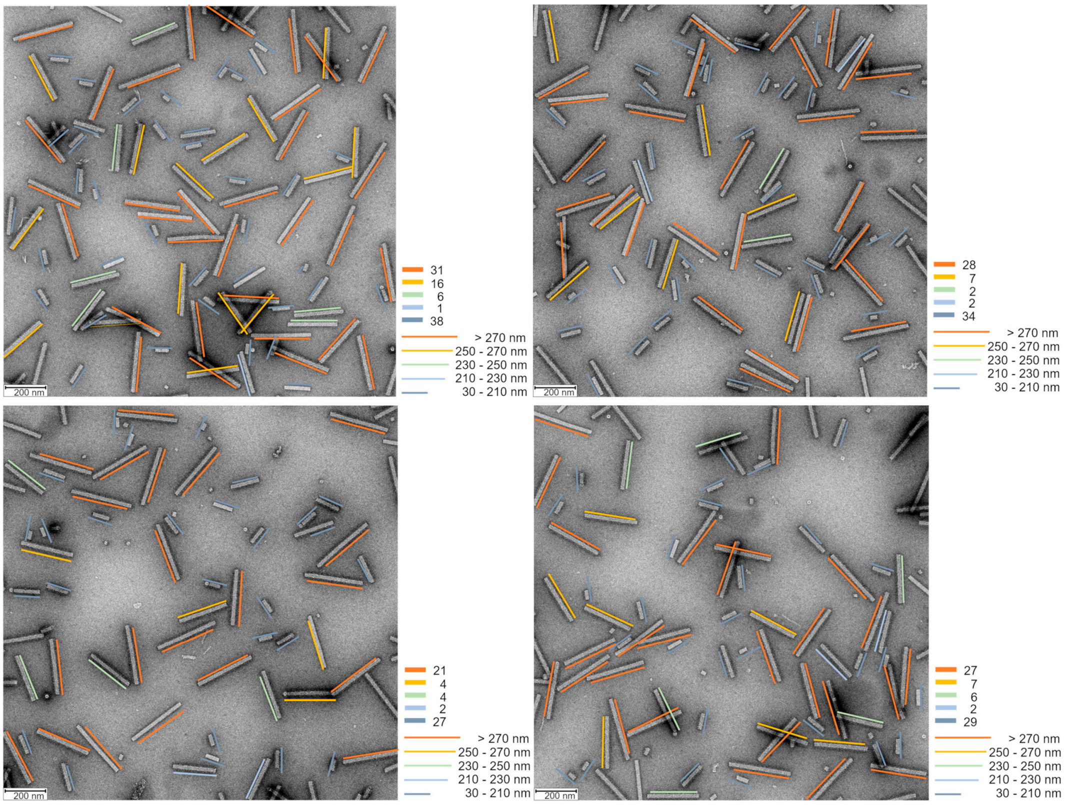

Figure S2. TEM images of TVCVPA with length class-specific color labels. A total of n=293 TVCVPA particles on randomly selected TEM grid areas were manually assigned to length classes, with all substantially shortened virions and VLPs (between 30 and 210 nm) in a single class (dark blue label). Classes and numbers of assigned particles are depicted next to each photograph. Circa 36% of the isolated virions were >270 nm in length, which is thus the most abundant length range observed. This distribution is comparable to that determined for virions isolated by a PEG solubility gradient-based method recently [1]. TVCVPA particles (0.1 mg/ml) purified from PEG raw precipitates via three-layer density barrier phase system (5/32/42% iodixanol) were negatively stained with 1% uranyl acetate on glow-discharged carbon-coated copper grids. Grids were examined with a TEM (Tecnai G2 Spirit BioTwin, Thermo Fisher Scientific) operated at 120 kV and equipped with a TemCam XF-416 (TVIPS GmbH).

S2.3. IgG-Binding of $TVCV_{PA}$ Adsorbed from Iodixanol Media to Solid Supports

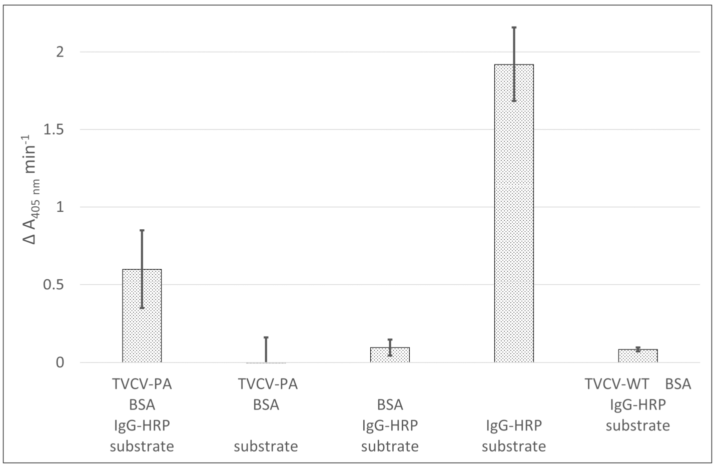

Figure S3. Assay of $TVCV_{PA}$ functionality after isolation via an iodixanol density barrier phase system and immobilization directly from the iodixanol-containing medium. $TVCV_{PA}$, or TVCV (5 µg each in 100 µl binding buffer [10 mM sodium-potassium phosphate, SPP, buffer with 137 mM NaCl, pH 7.8]), were immobilized on microtiter plate wells (overnight, 4 °C, high binding 96 well MaxiSorp clear F-bottom, Nunc). After three 5 min-washes at room temperature (RT) with 200 µl Tris-buffered saline (TBS: 0.05 M Tris, 0.15 M NaCl, pH 7.3) wells were blocked with 2% (w/v) bovine serum albumin (BSA, in TBS) for 1 h, RT. Washing was repeated as before. Wells were filled with 100 µl rabbit anti-mouse IgG-horseradish peroxidase conjugate (Sigma Aldrich A 9044, 10-20 mg/ml), diluted 1:100 in TBS and incubated for 1 h at RT. Unbound antibodies were removed by three washes (TBS, each 5 min, RT). The microtiter plate was transferred onto a precooled metal block (0 °C) and allowed to cool down before adding 100 µl of cold substrate (2.5 mM ABTS in 50 mM NaOAc, pH 5.0, 0.5 M H_2O_2). The plate was transferred to a microplate reader (Infinity M200 Pro, Tecan Trading AG, Swiss), the absorbance measured instantly at 405 nm, and absorbance changes recorded over 30 min time. TVCV, and blocked wells without TVCV were used as controls for nonspecific binding. All approaches were carried out as triplets.

S2.4. Isolation of $TVCV_{(PA)}$ via Iodixanol Density Barrier Phase Systems - Detailed Method

S2.4.1 Handling and Buffering Iodixanol

Iodixanol-containing solutions were diluted from a working solution (WS), which was prepared by adding one part of 3-(N-morpholino)propanesulfonic acid (MOPS)-buffer (150 mM MOPS-NaOH, 30 mM EDTA, pH 7.0; concentrated 6 × compared to the finally desired concentration as '6 × MOPS'), to five parts of the purchased 60% (w/v) iodixanol solution (OptiPrep, Serumwerk Bernburg AG, Germany), resulting in a buffered 50% (w/v) iodixanol solution. To set up the systems' density phases with the intended iodixanol concentrations, or media for isopycnic centrifugation, respectively, the WS was diluted with 1 × MOPS (i.e., 25 mM MOPS-NaOH, 5 mM EDTA, pH 7.0, prepared by diluting the 6 × MOPS concentrate with ultrapure water).

S2.4.2 Determination of TMV, TVCV and $TVCV_{PA}$ Buoyant Densities in Iodixanol

To determine the buoyant densities of TMV, TVCV and $TVCV_{PA}$ in iodixanol by isopycnic ultracentrifugation, two Re-Seal polyallomer centrifuge tubes (Seton Scientific, Part No. 9041; 13.5 ml volume) were filled with either 30% (w/v) (16.8 ml WS + 11.2 ml 1 × MOPS) or 40% (w/v) (22.4 ml WS + 5.6 ml 1 × MOPS) iodixanol solution using a syringe with a cannula leaving appropriate space for adding the virus sample. The sample (total virus quantity 1-10 mg, isolated according to Gooding and Hebert [102], or Wendlandt et al. [3], respectively) was added and tubes were filled up just below the beginning of the stem and balanced with the appropriate iodixanol-containing diluted solution, and sealed

(Tube Sealer PR 342428 Quick Seal, Beckman). Directly after centrifugation (vertical rotor VTi 65.1, Beckman; Optima LE-80K, Beckman; 15°C, 6 h, r_{max} 288,000 × *g* [RCF_{max}] = 55,000 rpm), the tubes were photographed (Figure S1). To determine the virions' buoyant densities, three samples per tube were taken by puncturing the tube's side wall with a needle and syringe (Sterican cannula 0.60 × 30 mm, Injekt F1 1 ml; Braun, Melsungen, Germany) and aspiring ~220 µl each: directly above the whitish-opaque virus band, directly from the band's center, and directly below the band, respectively. The refractive indices of the samples were measured by applying 20 µl to an Abbe-refractometer (Carl Zeiss AG, Oberkochen, Germany) in triplicates. The iodixanol concentration, corresponding to the refractive index, was determined using the iodixanol supplier's conversion table (Serumwerk Bernburg AG).

S2.4.3 Isolation of $TVCV_{PA}$ via Iodixanol Density Barrier Phase Systems

Sample preparation

For two SW 32 Ti tubes (Ultra-Clear Part No. 344058, Beckman Coulter) to be used, 40-60 g of symptomatic *Nicotiana benthamiana* leaves (inoculated mechanically with homogenate of $TVCV_{PA}$-infected leaves ground with 10 mM sodium potassium phosphate (SPP) buffer, pH 7.2 and carborundum dust; harvested at 18-21 days post inoculation [dpi]) were ground in a mortar in liquid nitrogen. The powder was suspended and thawed in 1.5 ml reducing SPP buffer (500 mM, pH 7.2, 1% 2-mercaptoethanol) per 1.0 g leaves, filtered through three layers of Miracloth (Millipore-Merck KGaA, Darmstadt, Germany) and clarified by centrifugation (4 °C, 10 min, 3000 × *g* [RCF_{max}] = 5233 rpm, Fiberlite F15-6 × 100y (Thermo Scientific), Heraeus Megafuge 16R (Heraeus Thermo Scientific)). The supernatant was transferred to 50 ml Falcon tubes. PEG-6000 (7.5% (w/v) for $TVCV_{PA}$; 8% (w/v) for TVCV); and 1% NaCl (all final concentrations, f.c.) were added to the tubes, which were then roller-mixed at 4 °C for at least 2 h. During this time, iodixanol density barrier phase systems were prepared as described below. The PEG precipitates formed in the filtered raw homogenates were sedimented (4 °C, 15 min, 10,000 × *g* [RCF_{max}] = 9554 rpm; Fiberlite F15-6 × 100y rotor), resuspended in 1 × MOPS and diluted in the same buffer to 34 ml, and kept at 4 °C until being laid onto two prefabricated three-layer iodixanol gradients (17 ml per each SW 32 Ti tube), as described below and depicted in Figure S4. Corresponding SW 32 Ti-buckets were balanced by transferring sample from one twin tube to the other, or by adding 1 × MOPS.

Density barrier phase system preparation

The systems phases were prepared by diluting the iodixanol working solution with 1 × MOPS (Table S2) to final iodixanol concentrations of 5%, 32%, and 42% (w/v). Volumes of 10 ml, 5 ml, and 5 ml, respectively, were stacked into SW 32 Ti tubes by underlayering the previous, less dense phase using a long canula and syringe (Figure S4). Due to balanced loading of the centrifuge, two SW 32 Ti tubes per 40-60 g leaf sample were equipped with equal density barrier phase systems. As soon as the PEG-precipitates of leaf extracts were resuspended and diluted to a volume of 34 ml, they were stacked on top of twin tube pairs, as shown in Figure S4.

Table S2. Preparation of stock solutions for a 5%/32%/42% (w/v) iodixanol density barrier phase system. Composition of solutions for two SW 32 Ti tubes with required volumes of iodixanol working solution (50% iodixanol in 6 × MOPS), and of diluent (1 × MOPS). An excess volume of 10% is considered.

Concentration of iodixanol (w/v)	Working solution (50% iodixanol)	1 × MOPS	Total volume	Phase volume needed for gradient setup
5% (loading phase)	2.2 ml	19.8 ml	22 ml	2 × 10 ml
32% (retention phase)	7.04 ml	3.96 ml	11 ml	2 × 5 ml
42% (cushion phase)	9.24 ml	1.76 ml	11 ml	2 × 5 ml
**Total volume**	18.48 ml	25.52 ml	44 ml	2 × 20 ml

In brief, samples were applied onto the top of the upper phase, without penetrating it, by pipetting against the wall of the inclined tube to allow the liquid to run down slowly. The filled tubes were inserted into the SW 32 Ti rotor buckets, balanced, and centrifuged (4 °C, 13.5-15 h, r_{max} 98,381 × *g* [RCF_{max}] = 24,000 rpm, rotor SW 32 Ti (Beckman), Beckman Optima LE-80K). Following ultracentrifugation, samples were collected by puncturing the tube's walls with a cannula and aspirating the sample into the attached syringe. Alternatively, the tube's content was fractionated by pipetting desired volumes successively from top to bottom. Fractions were analyzed via SDS-PAGE for the presence of $TVCV_{(PA)}$ CP and contaminants. Typically, 1-2 mg $TVCV_{PA}$ or 1-4 mg TVCV per g fresh leaf tissue were yielded.

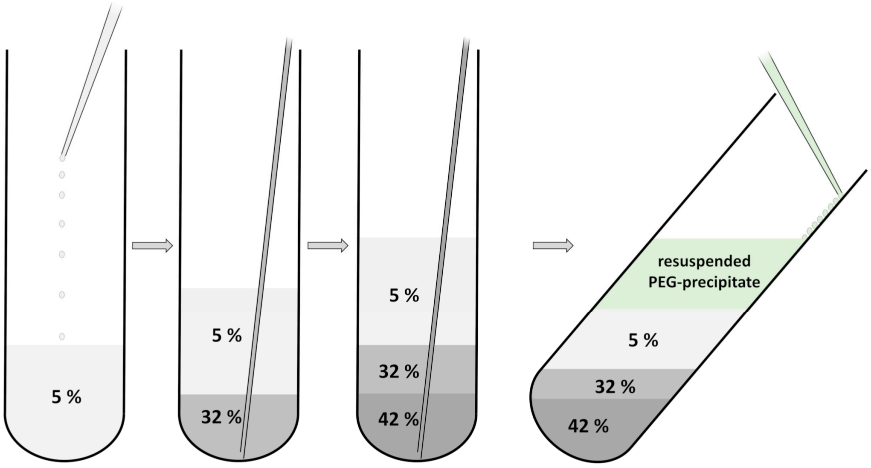

Figure S4. Illustration of the procedure for setting up three-phased density barrier systems of 5%/32%/42% iodixanol in an ultracentrifugation tube. First, the 5% phase is pipetted directly into the tube, before the 32% and 42% (w/v) phases are underlayered by slowly and smoothly expelling the appropriate solution from a syringe with a long cannula, ensuring a bubble-free process. Finally, the sample is carefully layered on top by tilting the tube gently upon slowly pipetting the sample to the tube's wall, allowing it to run down. The tube is erected gently after the sample has been applied.

Reference in Supplementary Materials S2.

1. Wendlandt, T.; Koch, C.; Britz, B.; Liedek, A.; Schmidt, N.; Werner, S.; Gleba, Y.; Vahidpour, F.; Welden, M.; Poghossian, A.; et al. Facile purification and use of tobamoviral nanocarriers for antibody-mediated display of a two-enzyme system. *Viruses* **2023**, *15*, doi:10.3390/v15091951.
